# Extraoral Detection of Biomarkers and Pathogens in Saliva: Comprehensive, Panoramic Review

**DOI:** 10.3390/bios16060345

**Published:** 2026-06-19

**Authors:** Aigerim Dyussupova, Aisha Ilyas, Aigerim Boranova, Yegor Shevchenko, Xeniya Terzapulo, Ansar Seitkali, Abduzhappar Gaipov, Olena Filchakova, Rostislav Bukasov

**Affiliations:** 1 Department of Chemistry, School of Sciences and Humanities, Nazarbayev University, 010000 Astana, Kazakhstan; aigerim.dyussupova@alumni.nu.edu.kz (A.D.); aisha.ilyas@alumni.nu.edu.kz (A.I.); aigerim.boranova@nu.edu.kz (A.B.); yegor.shevchenko@nu.edu.kz (Y.S.); xeniya.terzapulo@alumni.nu.edu.kz (X.T.); ansar.seitkali@nu.edu.kz (A.S.); 2 Department of Medicine, School of Medicine, Nazarbayev University, 010000 Astana, Kazakhstan; abduzhappar.gaipov@nu.edu.kz; 3 Department of Biology, School of Sciences and Humanities, Nazarbayev University, 010000 Astana, Kazakhstan; olena.filchakova@nu.edu.kz

**Keywords:** saliva, limit of detection (LOD), sensitivity, specificity, accuracy, cortisol, viruses, bacteria, cancer biomarkers

## Abstract

Human saliva is a heterogeneous bodily fluid with a complex composition, which contains antibodies, proteins, and viruses, making it applicable in clinical diagnosis. There are several advantages of the analysis of saliva samples over other biofluids, including a non-invasive and simple collection procedure for extraoral detection. Biomarker or pathogen detection in saliva can be performed with various methods: mass spectrometry, PCR, ELISA, electrochemical, and optical methods such as fluorescence, SPR, and SERS. The early detection of cancer and other disease biomarkers, as well as infectious agents, can be crucial for effective treatment and minimization of mortality from those diseases. The following paper reviews extraoral detection techniques to identify the most sensitive methods for diagnosing early and asymptomatic patients. The LODs collected and tabulated from 149 analytical papers, alongside the sensitivity, specificity, and sometimes the area under the curve (AUC) tabulated from 118 clinical studies, have all become parameters for the comparative quantitative analysis. Based on the limited but substantial number of analytical studies on the detection of cortisol in saliva (29), the electrochemical platforms demonstrated the highest sensitivity, with a geometric mean LOD of 11 pM. Within these methods, voltametric ones showed the best performance with 6 pM geometric mean LOD. Electrochemical techniques are then followed by immunoassay- and mass spectrometry-based platforms, with corresponding geometric average LOD values of 39.1 and 171 pM, respectively. However, clinical outcomes are at least as meaningful as LOD values. In terms of clinical analysis, ELISA and direct-SERS outperformed other methods, achieving balanced accuracy of approximately 87% and AUC values of 0.96 for direct SERS and 0.86 for ELISA. MS and PCR followed closely, with balanced accuracies around 84%. While the direct SERS is not yet widespread in clinical applications, its potential can be forged if the standardization issue is addressed.

## 1. Introduction

For the diagnosis of most complex diseases, tissue biopsy is often used [1]. However, this method is invasive, causes a lot of discomfort for patients, and for the analysis of the dynamic progression of diseases such as tumors or cancers, tissue biopsies have to be taken several times, which requires patients to endure the pain in the course of treatment, which is generally not feasible [1]. In addition, a single tissue biopsy can lead to unreliable results of biomarker expression and subsequently impact cancer type classification [2]. Moreover, tumor tissue biopsy provides only a snapshot of the disease, though it is considered a gold standard of cancer subtyping [3,4]. Depending on the location of the tumor, the accessibility of tumor tissue can also be problematic [5]. For these reasons, recent advancements in biomedical technology and cancer research have been focused on the development of new diagnostic tools through non-invasive sampling of bodily fluids, also known as liquid biopsy.

Liquid biopsy detects and analyzes different body fluids, such as urine or blood, instead of a specific tissue. It comprises various biological matrices, including nucleic acids, circulating tumor cells (CTCs), and exosomes. In addition to being non- or minimally invasive, it gives a better picture of disease heterogeneity and allows for real-time monitoring of the disease [1]. Among biofluids, saliva has attracted significant interest in disease diagnostics over the last few decades.

Saliva is a biofluid with a complex molecular composition. It consists of different antibodies, enzymes, hormones, proteins, and antimicrobial elements [6,7]. Saliva is mainly produced by three types of major salivary glands and diverse minor salivary glands in the oral cavity [8]. Compared to traditional diagnostic biofluids such as urine or blood, the collection of saliva samples is easy, non-invasive, and cheap. The history of saliva-based diagnostics dates back centuries. In ancient China, people used “The Rice Test” to detect if someone was guilty. The inability or difficulty in swallowing a mouthful of dry rice was indicative of someone’s “guilt” based on the idea that it results from anxiety, which inhibits the formation of a food bolus and swallowing [9]. The main pathophysiological idea behind this folktale is that people had an ancient understanding of the relationship between someone’s physical well-being and saliva production. The twentieth century is generally considered as a “modern age” of salivary diagnostics, utilizing saliva to detect different disease biomarkers [10]. Early attempts to use saliva as a diagnostic tool were based on the theoretical assumption that certain biomarkers that are present in blood might also be present in saliva. However, early attempts at saliva testing were not only limited by the knowledge of how blood biomarkers make their way to saliva or vice versa, but also by the lack of standardized methods of saliva collection and storage, as well as detection techniques [11]. Nowadays, the physiology of saliva is well understood, and there exist numerous sensitive detection techniques such as enzyme-linked immunosorbent assay (ELISA) and quantitative reverse transcriptase–polymerase chain reaction (qRT-PCR) [12,13].

Nowadays, high accessibility of saliva makes it highly attractive for biomarker research. Especially during the pandemic, saliva testing proved to be effective in terms of time and cost for the detection of COVID-19. Detection sensitivity of viral genome varies from <100 copies mL^−1^ to 0.38 copies µL^−1^, which is comparable to traditional polymerase chain reaction (PCR) [14,15]. Saliva testing also shows its diagnostic value in the detection of neurodegenerative diseases, immunodeficiency, cancer, diabetes, renal, and oral diseases [16]. Several studies claim that saliva testing is possible due to a positive correlation between biomarker concentration in blood and saliva. For example, several clinical studies demonstrated positive correlations between the concentration of free cortisol in saliva and blood, which is useful for the analysis of psychobiological states [17,18,19]. These positive correlations are due to the small size of cortisol and its lipophilicity, allowing its diffusion through glandular and epithelial cell membranes. However, these correlations are not always robust enough to substitute one collection method for another. These relationships differ by biomarker type [20].

Numerous studies demonstrated the application of saliva-based diagnosis of neurodegenerative diseases such as autism spectrum disorder (ASD), Parkinson’s, and Alzheimer’s disease (AD) [21,22,23,24,25]. For example, Hicks et al. investigated the potential of salivary-based miRNAs to serve as a diagnostic screening tool of autism spectrum disorder (ASD), a neurodevelopmental disorder that is commonly characterized by a shortfall in communication and social interaction [21,26]. The multivariate ROC analysis revealed 100% sensitivity, 95.6% specificity, and an area under the curve (AUC) of 0.974. Liang et al. applied faster ultra-performance liquid chromatography (FUPLC) mass spectrometry (MS) coupled with a multivariate statistical method to determine metabolic changes in the salivary metabolome in patients with Alzheimer’s disease [24]. Saliva testing is also widely used in cancer and tumor detection. Numerous studies demonstrate the potential of saliva-based detection of biomarkers for the early diagnosis of breast, lung, gastric, pancreatic, and ovarian cancer [27,28,29,30,31,32]. Overall, the complex molecular composition of saliva makes it a valuable biofluid for the analysis of different biomarkers, including miRNAs, DNAs, proteins, amino acids, bacteria, exosomes, and different metabolites [7,29,33,34,35,36].

Apart from the complex biological composition of saliva that enables the detection of various analytes, saliva can be analyzed using various methods such as different types of mass spectrometry (e.g., UPLC-MS [27], MALDI-MS [37], MALDI-TOF-MS [38]), ELISA [39], electrochemical methods (e.g., electrochemical impedance spectroscopy (EIS) [40,41], electrochemical immunosensors [42], enzyme biosensors [43]), surface-enhanced Raman scattering, and others. For example, ELISA, which is considered a gold standard of immunoassays nowadays, was first introduced in the 1970s as an alternative replacement for radioactive labels used in conventional radioimmunoassays [44]. ELISA was first used to detect the level of IgG in rabbit serum. Within the same year, researchers were able to quantify chorionic gonadotropin in urine by using horseradish peroxidase. Since that time, the ELISA method has been used universally in different routine laboratory research for different applications [45]. For example, the ELISA method was used for the detection of salivary C-reactive protein (CRP) for the diagnosis of neonatal pneumonia in the work of Omran et al. [46]. At the cut-off value of 3.8 ng L^−1^, salivary CRP demonstrated 91.4% sensitivity and 80.9% specificity.

In addition to ELISA, electrochemical-based immunoassays and biosensors are also widely used to detect and analyze different biomarkers present in the saliva [47,48]. For example, Adornetto et al. applied an electrochemical immunoassay method for the screening of celiac disease in saliva samples [35]. Celiac disease is a gluten-dependent autoimmune disease that is usually found in genetically susceptible individuals. In their work, they used magnetic beads with anti-transglutaminase (tTG), which react with anti-tTG IgA antibodies in positive saliva samples. In this approach, they conjugated anti-human IgA with alkaline phosphate (AP) enzyme and applied a strip of eight magnetized screen-printed electrodes as the electrochemical transducer. The clinical sensitivity and specificity reached 95% and 96%, respectively. The proposed method allowed Adornetto et al. to overcome problems related to high viscosity and low concentration of antigens present in the medium.

Mass spectrometry combined with liquid chromatography is also widely used in saliva analysis. This combination has a wide range of applications in the diagnosis of cancer [49], neurodegenerative disorders [50], and other diseases [28]. For example, Qun et al. applied ultra-performance liquid chromatography (FUPLC) mass spectrometry (MS) coupled with a multivariate statistical method to detect metabolic changes in the salivary metabolome of patients with Alzheimer’s disease. The analysis of 474 saliva samples (256 positive, 218 negative) resulted in 99.4% sensitivity and 98.2% accuracy. The proposed method demonstrated potential application of salivary metabolomics in a clinical setting for the early diagnosis of AD.

The research interest in saliva as a biological diagnostic fluid keeps growing. It can be seen from the number of review articles. Based on CAS SciFinder search results dated February 8, 2026, the number of review articles (with DOIs) on saliva-related topics published in 2025 was 138% higher than the number published in 2015 (490 vs. 206). Moreover, there is an increasing trend in publications discussing clinical applications of saliva in the diagnosis of various diseases and health conditions. For example, the review article by Huang et al. discusses clinical applications of salivary biomarkers in the diagnostics of cancer, periodontal, and systemic diseases [16]. There are also review articles that focus specifically on certain cases, such as in the work of Chai et al., where they discussed the saliva-based diagnostic methods for malaria detection [51]. In another review, Goldoni et al. focused on the study of various biosensors for the detection of aging-related neurodegenerative diseases [52]. The study comprises mostly analytical studies with a future goal to stimulate clinical applications of salivary biosensors. In addition, Meleti et al. presented a meta-analysis of studies about saliva-based detection of various diseases, including cancer, neurologic, and cardiovascular diseases [6], whereas a recent comprehensive review by Yulianto et al. discussed the use of carbon-based electrochemical biosensors for detecting salivary biomarkers in oral diseases and dental issues such as caries, periodontitis, and oral squamous cell carcinoma [53].

Compared to previously discussed reviews, herein we analyzed a broader scope of research articles about saliva-based detection, which include both analytical and clinical studies. Our analysis was based on the comparison of multiple performance parameters, such as limit of detection (LOD) in case of analytical studies, and sensitivity/specificity values in case of clinical publications. In early disease diagnostics, a low limit of detection (LOD) matters because the lower the LOD, the lower the false-negative rate and thus the higher the chance of determining infected people, especially at the early stage of disease development. For example, Arnout et al. reported that a 10-fold increase in LOD is likely to increase the rate of false-negative results by 13%, which equals missing one patient in eight infected patients. This result has a meaningful clinical and epidemiological consequence in COVID-19 testing [54]. Generally, it is essential to understand that the outcome of a specific treatment strategy depends on the stage of disease (e.g., cancer) at the time of diagnosis [55]. For instance, according to the Cancer Stat Fact Sheet of the National Institute of Health (USA), patients diagnosed with stage 3 or 4 prostate cancers have a 5-year survival rate of 28%, while for patients diagnosed with stage 1 and 2, the 5-year survival rate is 100% (Cancer Stat Fact Sheets, NIH) [56]. All previously reported numbers demonstrate the importance of low LOD in early-disease diagnostics and the need for developing dvanced screening and diagnostic techniques.

In addition, we discuss saliva-based detection of different biomarkers, including bacteria, toxins, viruses, cancer, and non-cancer biomarkers. Moreover, we cover clinical applications of saliva-based testing and review different methods, including mass spectrometry, electrochemical methods, immunoassays with optical detection (e.g., ELISA, lateral-flow immunoassays, SERS-based immunoassays), and other methods. The review does NOT include the emerging area of sensors, which are implanted or inserted in the mouth; however, it covers more extensive literature about undoubtedly non-invasive extraoral analysis of saliva. Taken together, we believe this review might be helpful to demonstrate recent developments in saliva-based clinical diagnostics and influence the development of non-invasive treatment strategies and therapies.

## 2. Detection of Bacteria and Viruses in Saliva

Infectious diseases, mostly caused by pathogenic microorganisms such as viruses and bacteria, are contributing to mortality worldwide [57]. Infectious diseases can be exponentially transmitted among people in a short period of time, and it is estimated that more than half of the world’s population is at risk of infectious diseases [58,59]. Due to their explosive impacts and absence of efficient control systems, infectious diseases were one of the most dangerous threats to humanity in the past. The Black Death in the 14th century killed about 25 million people in 5 years [60], smallpox accounted for about 10% of deaths in 18th-century Europe [61], the influenza pandemic of 1918 killed 50–100 million people [62]. Even with modern medicine, due to the present context of globalization, viral infections have an enormous negative impact on health and the economy, as demonstrated by the recent COVID-19 pandemic that resulted in the death of about 6.9 million people in about 3 years [63]. One of the most efficient strategies for the management of infectious diseases is the control of the infection source, which requires early detection, isolation, and treatment of infected individuals [64,65]. “Ultrasensitivity” is the key factor in achieving early detection and diagnosing asymptomatic patients [66,67,68].

Human saliva has been called a “mirror of the body’s health” [69]. Saliva comprises oral epithelial cells, salivary gland secretions, gingival crevicular fluid, viruses, fungi, and bacteria [70]. The collection of the saliva sample is simple, non-invasive, does not require skilled personnel or specialized instruments, has a minimal risk of cross-contamination, and the samples are easy to store [71,72]. Therefore, the saliva sample can be directly collected by the patient, which is useful for the prevention of the transmission of infectious diseases [73].

One of the widely used tools for the detection of viruses and bacteria is the polymerase chain reaction. Polymerase chain reaction is a laboratory technique used to amplify a specific segment of DNA from a complex pool. It was invented by Kary Mullis in 1983 and is now a widely used method in molecular biology and genetics [74].

The major components of each PCR cycle include template DNA, primers, DNA polymerase, and nucleotides. The DNA polymerase links nucleotides together to form a final PCR product. The four major bases—adenine (A), guanine (G), thymine (T), cytosine (C)—present in individual nucleotides constitute the backbone of the resultant PCR product. Primers are short DNA fragments that define the region in DNA that needs to be amplified during the PCR cycle. The PCR cycle consists of three major steps: (1) denaturation, (2) annealing, and (3) extension. In the denaturation step, double-stranded DNA is heated to a high temperature, around 95 °C. This leads to a breakage of hydrogen between strands, leading to two single strands of DNA. In the annealing step, short DNA primers that are complementary to the target DNA sequence are bound to single DNA strands. In the extension step, the DNA polymerase enzyme, along with deoxynucleotide triphosphates (dNTPs), is added to the mixture, and the DNA polymerase extends the primers by adding new nucleotides to the 3′ end of the primers, resulting in a new DNA strand. The amplified DNA fragments can be analyzed by various methods, such as gel electrophoresis or sequencing [74].

PCR has several advantages. It is a simple, sensitive method with the potential to produce millions to billions of copies of a desired DNA fragment. Moreover, PCR can be useful for both qualitative and quantitative analyses. For example, Min et al. used the PCR method not only for screening of deregulated miRNAs in saliva, but also for quantification for the early diagnosis of Hand, Foot and Mouth disease (HFMD) in pediatric patients [75].

Another nucleic acid amplification method widely used for the detection of pathogens is loop-mediated isothermal amplification (LAMP), introduced by Notomi et al. in 2000 [76]. Four designed primers and DNA polymerase are employed to amplify six different regions on the target DNA. Under isothermal conditions (63–65 °C), LAMP can amplify up to 10^9^ copies in less than an hour [76].

The analytical detection of viruses and bacteria in clinical or spiked saliva samples using techniques such as amplification-based (PCR, LAMP), electrochemical, surface-enhanced Raman spectroscopy, and chemiluminescence is presented in Table 1.

According to the World Health Organization, as of April 12, 2023, more than 750 million cases of COVID-19 and about 6.9 million COVID-19-related deaths were confirmed [63]. The symptoms of COVID-19 are non-specific and range from no symptoms to pneumonia; therefore, it is complicated to specifically differentiate SARS-CoV-2 infection from other respiratory viruses or the common cold [132]. However, the early detection of COVID-19 is highly significant in the prevention of further transmission and avoiding mortality [133,134,135]. Due to the tremendous impact of the COVID-19 pandemic on society, hundreds of SARS-CoV-2 detection methods were established, which are extensively reviewed in the literature [136]. Because of the minimum discomfort and the possibility of self-collection, some of the detection techniques were focused on the detection of SARS-CoV-2 in saliva. Hunt et al. developed a point-of-care lyophilized cell-free protein synthesis (CFPS) biosensor that generates a luminescent signal within 7–15 min after the addition of the SARS-CoV-2 saliva sample [122]. Chromatography paper was chosen as an optimal matrix since it facilitates rapid reaction rates. High protein synthesis and lyophilized CFPS reagents were embedded in chromatography paper and housed in a recyclable LDPE (low-density polyethylene) test cassette. The estimated cost of the following test is less than 0.50 USD, and the reagents retain their functionality for up to seven weeks at room temperature. Toehold switch riboregulators were engineered to express the bioluminescent (NanoLuc) reporter signal in response to viral RNA sequences. Protein expression of a native CFPS reaction was completely inhibited with the addition of >10% saliva *v*/*v*, and no signal in the presence of 40 nM SARS-CoV-2 RNA was obtained. However, the addition of the murine RNase inhibitor (mRI) to the reaction mixture restored the expression of the protein, and the LOD of the following test was around 10 nM of viral RNA. According to the authors, the fast response time (7–15 min) of the test is due to the small size of the NanoLuc reporter (510 base pairs), which can speed up the detection by rapid activation [122].

Another SARS-CoV-2 detection method with the limit of detection down to 0.8 copies per microliter and an assay time of two hours was developed by Najjar et al. [137]. A 3D-printed LOC platform with a multiplexed electrochemical sensor that can detect both viral RNA and protein, where the saliva is perfused through a single microfluidic channel, was established. To ensure the highly sensitive detection from the untreated saliva, the sample is mixed with proteinase K solution and heated to 55 °C for 15 min for purification. Then, to lyse the sample and inactivate the nuclease, the temperature is increased to 95 °C for 5 min using integrated high-power resistors. The nucleic acid is captured by the polyethersulfone membrane, and the reaction chamber is heated to 95 °C for 3–5 min to denature the potential reaction inhibitors. After RNA is amplified by LAMP incubation at 65 °C for 30 min, followed by incubation at 37 °C for 30 more minutes for the CRISPR reaction, the sample is pumped over the electrochemical sensor chip to incubate. LOD of 0.8 copies per microliter was obtained by optimizing the reaction by using 1 nM of reporter probe for CRISPR and 5 min incubation for signal production. In the case of antibody detection, the saliva is directly pumped over the electrochemical sensor chip and incubated. The chip is washed with phosphate-buffered saline with Tween 20 and polystreptavidin–horseradish peroxidase, a precipitating form of tetramethylbenzidine is added, and the chip is read using a potentiostat. Clinical testing of the following method was performed by using 19 PCR-positive and 11 negative SARS-CoV-2 saliva samples. As a result, 100% accurate diagnosis using Orf1ab RNA (via LAMP and CRISPR-based detection), Spike S1 receptor-binding-domain antigen (antibody-based detection) of SARS-CoV-2, and multiplex detection was observed. Despite the comparatively long assay time, the multiplex, highly sensitive, automated, and point-of-care detection of the virus was demonstrated by Najjar et al. [137].

**Figure 1:**
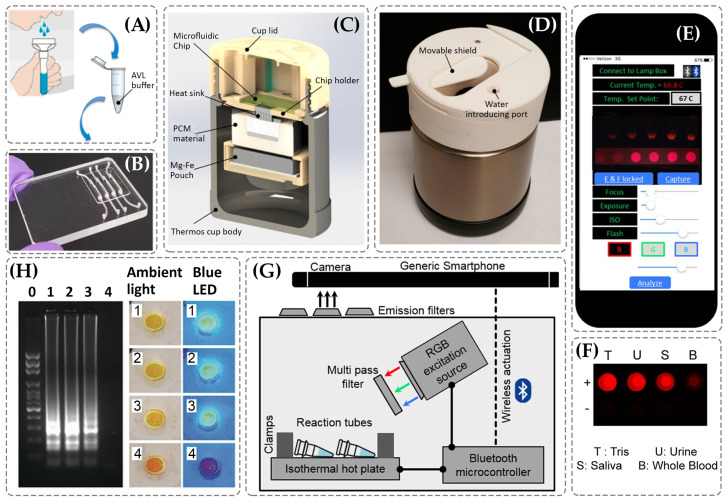
Detection of Zika virus using reverse transcriptase loop-mediated isothermal amplification (RT-LAMP). (**A**) Collection of saliva in a tube and lysis. (**B**) Nucleic acid extraction in the microfluidic cassette. (**C**,**D**) Chemically heated cup. Adapted with permission from Song et al. [93]. Copyright © 2016 American Chemical Society. (**E**) A smartphone application used to monitor and adjust the temperature of the heater and laser excitation. (**F**) Images of positive and negative Zika virus samples detected using RT-LAMP. (**G**) Scheme of the RT-LAMP-based detection using an LED excitation source and a Bluetooth microcontroller. Adapted from Priye et al. [96], licensed under CC BY 4.0. Copyright © 2017 Springer Nature. (**H**) Photographs of RT-LAMP detection results under ambient light and blue LED flashlight (images 1–3 contain Zika virus; image 4 shows the negative control). Gel electrophoresis of RT-LAMP amplicons is shown on the right. Adapted with permission from Jiang et al. [98]. Copyright © 2018 Wiley-VCH Verlag GmbH & Co. KGaA, Weinheim.

The point-of-care (POC) detection of the Zika virus using loop-mediated isothermal amplification (LAMP) is demonstrated in Figure 1 [93]. POC devices enable fast, early, and inexpensive testing near the patient. The disposable microfluidic cassette in a cup (the estimated cost of the test is 2 USD) was applied for the detection of Zika virus using reverse transcription LAMP by Song et al. At first, the saliva samples were collected and lysed in Qiagen binding/lysis (AVL) buffer (Figure 1A). After, the nucleic acid is extracted from the lysed sample through the isolation membrane of four independent, multifunctional, isothermal amplification reactors in the microfluidic cassette (Figure 1B). As shown in Figure 1C,D, the point-of-care device (chemically heated cup) consists of a thermos cup body, a cup lid, a chip holder, and a disposable Mg-Fe alloy pack that acts as a heat source when interacting with water. Phase-change material that melts at 68 °C was introduced to regulate the temperature in the amplification reactors. The aluminum heat sink was embedded into the phase-change material to enhance the heat transfer to the microfluidic cassette. Finally, after incubating for 40 min, the results detectable by the naked eye or cell phone were observed, and detection down to five plaque-forming units of Zika virus was achieved [93].

Jiang et al. applied a coffee mug to provide the isothermal conditions for RT-LAMP-based detection of the Zika virus from saliva samples [98]. Identification inside the platform consists of three parts: (1) a buffer part for the sample lysis, RNA enrichment, and purification using buffers; (2) a mixing unit; (3) a detection unit that contains a polycarbonate sheet with a well in the center, a double-sided adhesive tape, a laminated paper pad, and 25 mL of RT-LAMP buffer. After incubation for 25 min, the results can be observed by the naked eye (with pre-added SYBR green dye) or using a blue LED flashlight. (Figure 1H) Within the assay time of 50 min, the LOD of 0.5 plaque-forming unit (PFU) of the Zika virus was attained in saliva and urine samples, and the LOD of 0.1 PFU in water [98].

Multiplex detection of Zika, chikungunya, and dengue viruses by using a portable LAMP box and a smartphone was reported by Priye et al. [96]. The applied quenching of unincorporated amplification signal reporters (QUASR) technique offered very bright signals and reduced the number of false positives in comparison to other reporters. Furthermore, QUASR was compatible with complex sample matrices, and saliva or blood samples were added into the RT-LAMP reaction mixtures without pre-treatment, as pre-heating the sample had little impact on the LOD. The scheme of the following detection method is demonstrated in Figure 1G. The results can be visualized using smartphones with a novel algorithm utilizing chromaticity to analyze fluorescence signals; the discrimination of positive/negative signals was improved by five-fold using the smartphones when compared to traditional RGB intensity sensors or the naked eye. Also, as depicted in Figure 1E, a low-powered isothermal heating module, multicolor LED excitation can be controlled using the application on the smartphone via Bluetooth. The app analyzes the images taken through the phone camera and detects the multiplex QUASR assay signals. The assay time is about 40 min, and viruses could be detected in the Tris buffer, urine, saliva, and blood successfully (Figure 1F) [96].

Until the COVID-19 pandemic, tuberculosis (TB) was the leading cause of death worldwide from a single infectious agent. Without treatment, the death rate from tuberculosis disease is about 50%, and in 2021, there were an estimated 1.6 million TB deaths [138]. The accurate early diagnosis of TB is crucial in reducing the risk of transmitting the disease to others and in initiating an early anti-tubercular therapy [138]. The detection down to 10 fg mL^−1^ of *Mycobacterium tuberculosis* using immuno-PCR (ELISA with PCR) with gold nanoparticles (NP) was achieved by Singh et al. [83]. The specific binding of primary antibodies on the surface of AuNP to the secondary antibodies was confirmed using TEM, which led to the LOD 10^−12^ g L^−1^, less than the analogous ELISA. Furthermore, the LOD of the purified target by applying Magnetic bead–AuNP-immuno-PCR (liquid) was ten-fold less than the conventional solid immuno-PCR. The liquid format permits a thorough washing of the captured antigen and magnetic beads and AuNPs; thus, the unbound antigens/antibodies are removed. As a result, nonspecific binding is decreased, and the background signals are diminished.

*Pseudomonas aeruginosa* is an opportunistic pathogen that poses a threat to people with cystic fibrosis, severe burns, and immunocompromised patients with cancer [139]. The direct detection of *P. aeruginosa* is complicated because cells usually firmly anchor on the tissues with biofilm; therefore, *P. aeruginosa* is detected using the biomarker pyocyanin [140]. A novel whole-cell redox reactivation/cycling module based on the bioelectrocatalysis of electroactive bacteria (*Shewanella oneidensis* MR-1 cells) was employed to amplify the signals from pyocyanin [113]. As pyocyanin is a redox-active molecule, conventional cyclic voltammetry analysis can be used for detection. However, the oxidized pyocyanin cannot be immediately reduced during the single scanning process, which means that each pyocyanin is registered only once by the electrode. The study of Yang et al. to achieve more sensitive detection of *P. aeruginosa* employed the *Shewanella oneidensis* MR-1 cells as the bioelectric catalyst and lactate as the electron donor. This led to the regeneration of reductive pyocyanin from its oxidative state and enabled the electrode’s repeated registration of the target. The sensitivity of the following method was 1314 ± 27 nA nM^−1^ of pyocyanin, which is about 400 times more than the conventional cyclic voltammetry without *S. oneidensis*. Also, the obtained LOD of 47 pM is considerably lower than the concentration of pyocyanin in the patients infected with *P. aeruginosa*, meaning that the following method can be used for the early detection of the pathogen [113].

Table 2 shows the overall 55 clinical studies in the detection of viruses (44) and bacteria (11) in saliva using techniques such as electrochemical/electrochemiluminescence (11 studies), ELISA (7), PCR (24), LAMP (8), and Raman/fluorescence (6). The studies with a sample size of less than 20 were excluded from calculations of average and median sensitivity, specificity, and accuracy, and are marked with an asterisk (*).

Raman/SERS/fluorescence were revealed as one of the most accurate methods among the considered diagnostic studies, with an average sensitivity/specificity/accuracy of 99%/99%/99% (for six studies). The clinical methods with larger data sets, such as PCR and LAMP, had average diagnostic accuracy of 95.8% and 94.3%, respectively. The clinical studies based on the LAMP method demonstrated the high average specificity of 99.3%. Two electrochemical studies demonstrated a 100% detection for Norovirus ^52^ and SARS-CoV-2 [137]. Other studies that reported 100% accurate detection include the diagnosis of porcine epidemic diarrhea virus [152] and swine vesicular disease virus [153] in animals using ELISA. Both studies revealed that the genome detection in saliva (or oral fluid) was more sensitive than in serum (sensitivity of 100% vs. 87% for porcine epidemic diarrhea virus) [152]. Swine vesicular disease virus detection in oral fluids was possible even in groups with subclinical disease [153].

The highly sensitive, point-of-care, and multiplex detection of 11 viruses, including coronaviruses, influenza, respiratory, adenovirus, and metapneumoviruses, using SERS and Support Vector Machine (SVM) within 20 min was achieved by Yang et al. [183]. Accurate detection (100%) of seven out of 11 viruses was reported; the overall classification accuracy was around 99.95%. 15,600 viral and healthy saliva spectra were analyzed, 70% of the spectra were used for training the SVM [183].

Hernandez et al. evaluated the performance of Agena MassARRAY ^®^ in detecting SARS-CoV-2 in 60 saliva samples [89]. The method employs the extraction of the viral RNA from saliva on the automated chemagicTM 360, followed by the reverse transcriptase polymerase chain reaction using MS2 phageRNA internal control. The PCR products were desalted, transferred to a silicon chip with pre-spotted matrix crystals, and loaded into a matrix-assisted laser desorption/ionization time-of-flight (MALDI-TOF) mass spectrometer. The assay was designed to detect five viral targets: three in the nucleocapsid gene and two in the ORF1ab gene. If the internal control and more than two viral targets were detected, the results were interpreted as positive; if no internal control was found, the results were invalid, and the sample was subjected to rerun. The limit of detection of 1562 copies mL^−1^ and sensitivity and specificity of 97% and 100% (in comparison with conventional RT-PCR) were reported using the most sensitive nucleocapsid 2 gene [89].

Janikova et al. tested whether SARS-CoV-2 RNA can be directly (without extractions) detected in saliva [101]. However, whole saliva and saliva collected using Salivette (collection by passive drooling) interfered with the RT-LAMP analysis, even after the protease treatment and using Chelex-100. Despite this, the successful detection of the virus in saliva was possible after the addition of the RNase inhibitor, with the LOD of six copies per reaction in whole saliva, 12 copies per reaction in Salivette saliva samples, and 100% detection was achieved for 10 samples [101].

The comparative study of multiplex SARS-CoV-2, influenza A/B, and respiratory syncytial virus (RSV) in saliva and nasopharyngeal samples using TaqMan RT-LAMP was performed by Neopane et al. [92]. The limit of detection of respiratory viruses in saliva and nasopharyngeal swab was 500 and 100 copies/reaction for SARS-CoV-2 and influenza A/B, 100 and 10 copies/reaction for RSV. Despite the LOD in saliva being higher than in nasopharyngeal swabs, 100% accurate diagnosis in saliva was detected for 75 SARS-CoV-2 samples. Furthermore, 16 spiked saliva samples demonstrated 100% agreement with the expected result of co-infection for SARS-CoV-2, influenza, and RSV [92].

Liu et al. proposed a method based on PCR and G-quadruplex DNAzyme as a color label to detect *Helicobacter pylori* (the main cause of chronic gastritis) in saliva [160]. The PCR synthesizes a DNA aptamer of DNAzyme at the 3′ end of the PCR products. After the aptamer binding of hemin, the formed G-quadruplex DNAzyme could catalyze the H_2_O_2_-mediated oxidation of colorless 2,2′-azino-bis(3-ethylbenzthiazoline)-6-sulfonic acid (ABTS) to green-colored ABTS. The colorimetric result can be visualized by the naked eye (LOD of 100 pg/reaction) or UV–Vis spectroscopy. A 100% clinical agreement was obtained for 20 saliva samples tested by this method [160].

The detection of Norwalk virus (the most common cause of acute gastroenteritis) using two different methods, the Luminex fluorometric and Meso Scale Discovery (MSD) electrochemiluminescence immunoassays, was performed by Griffin et al. for 20 specimens [141]. The saliva samples were collected at the challenge, and 2 weeks and 40 days after the challenge, and as a result, both immunoassays achieved 100% sensitivity and specificity. The assay results were optimized by using IgG instead of less sensitive IgA, e.g., for MSD results, the sensitivity from IgG and IgA was 100% vs. 33%, respectively. The Luminex immunoassay results were optimized by a 1:4 dilution in the well, which was required because of the viscosity of the saliva. Lower saliva dilutions can clog filter-bottom microplates, causing a high proportion of unsuccessful spurious results due to increased non-specific binding. Furthermore, adjusting responses to the norovirus antigens for responses to the protein purification tag, glutathione-S-transferase (GST), improved the diagnosis using the Luminex immunoassay [141].

**Figure 2:**
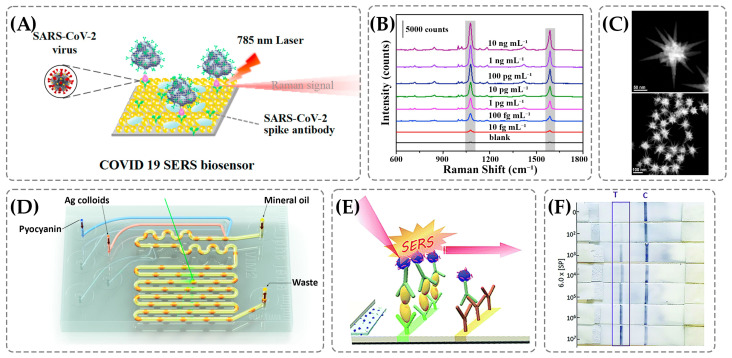
Surface-enhanced Raman spectroscopy (SERS) for the detection of viruses and bacteria in saliva. (**A**) Scheme of the SERS immunoassay. (**B**) Average SERS spectra of the sandwich immunoassay structure with SARS-CoV-2 spike protein in saliva. Adapted with permission from Zhang et al. [126]. Copyright © 2021 Elsevier B.V. (**C**) Transmission electron microscopy (TEM) images of single and multiple gold nanostars. Adapted from Atta et al. [127], licensed under CC BY 4.0. Copyright © 2021 American Chemical Society. (**D**) Droplet-based microfluidic chip used for SERS measurements. Adapted from Zukovskaja et al. [124], licensed under CC BY 4.0. Copyright © 2017 MDPI. (**E**) Group A streptococcus (SP) detection using SERS-based lateral flow immunoassay (LFIA) test strips (T: test line; C: control line). (**F**) Images of LFIA strips showing different test line (T) colors with increasing SP concentration in the range of 0–10^7^ CFU mL^−1^. Adapted with permission from Eryilmaz et al. [125]. Copyright © 2019 The Royal Society of Chemistry.

Surface-enhanced Raman spectroscopy enables the highly sensitive, multiplex, fast detection of the various analytes, including viruses and bacteria, in saliva, as shown in Figure 2. The detection of SARS-CoV-2 spike protein using SERS immunoassay is demonstrated in Figure 2A. The SERS substrate was constructed by using the novel oil/water/oil three-phase liquid–liquid interfaces self-assembly method. As a result, two layers of dense and uniform Au nanoparticle films were formed to ensure the reproducibility and sensitivity of the SERS immunoassay. Then, through the immunoreaction between the viral spike antibody-modified SERS substrate, spike antigen protein, and Ag nanoparticles labeled with Raman reporter, the analyte was identified. The LOD in phosphate-buffered saline was 0.77 fg mL^−1^. The detection in saliva was the best among other body fluids applied: 6.07 fg mL^−1^ in untreated saliva (Figure 2B), 7.6 fg mL^−1^ in serum, and 0.1 pg mL^−1^ in blood, respectively [126]. In the research article by Eryilmaz et al., a SERS-based lateral flow immunoassay was successfully applied to detect Group A streptococcus in saliva down to 0.2 CFU mL^−1^ (about one cell) within 30 min (Figure 2E,F) [125]. The SERS-based microfluidic LoC technique was established by Zukovskaja [124]. The LOD of 0.5 µM and the detection of pyocyanin (a metabolite specific to *Pseudomonas aeruginosa*) in three clinical saliva samples were obtained. The glass syringes (Figure 2D) were filled with different solutions and were connected to the chip by Teflon capillaries, and the solutions were delivered into the chip using the computer-controlled pump system, while the mineral oil was required to provide a segmented continuous flow. In order to obtain the most intensive and stable signals, the addition of salts should be avoided since the presence of KCl or other salts results in the over-aggregation of the nanoparticles, leading to the formation of large clusters that do not support high electromagnetic enhancements. Another optimization strategy involving the modification of the gold nanostar morphology (SERS substrate) by changing the concentration of AgNO_3_ was performed by Atta et al. [127]. The maximum SERS enhancement and LOD of 0.05 nM of pyocyanin in drinking water and 0.4 nM in saliva were obtained in gold nanostars with maximum spike lengths and sharp spikes (Figure 2C) that were produced by increasing the concentration of silver nitrate from 15 to 120 µM.

The average LOD for the detection methods in Table 1 was not calculated because the units for the limit of detection vary across studies (e.g., PFU mL^−1^, CFU mL^−1^, ng mL^−1^, cells mL^−1^, etc.). The molecular weight of viruses and bacteria can also fluctuate, so the accuracy of the average LODs estimated by the conversion to one unit can be limited. Nevertheless, the performance of the detection techniques in identifying viruses and bacteria can be compared by employing the clinical studies presented in Table 2. Viruses and bacteria in saliva are usually detected using nucleic acid amplification techniques. The most popular method is PCR, which accounts for 16 of 55 analytical articles available in Table 1 and 24 out of 54 clinical studies from Table 2. The average sensitivity/specificity/accuracy of PCR-based diagnosis was about 90/99/96%. PCR is a very sensitive assay, also considered a gold standard for the detection of viruses, including SARS-CoV-2 [185,186,187]. Despite its practical utility, PCR has several limitations. Primers can nonspecifically bind to similar sequences on the template DNA. Also, to design primers for PCR, some prior sequence information is required [74]. DNA polymerase enzyme, which is required for PCR, is susceptible to errors [74,188]. Consequently, PCR analysis requires a specialist throughout the whole detection procedure; moreover, PCR needs an expensive instrument, temperature management, and has a sample-to-result time of several hours [189,190], which poses a limit to the applicability of PCR in large-scale monitoring. An alternative to PCR in virus and bacteria detection is LAMP, which is the next widely applied technique (14 analytical and eight clinical studies). The diagnostic accuracy of LAMP is comparable to PCR, 94.3% vs. 95.8%. The advantages of LAMP in comparison to PCR include the faster detection time [191,192], simplicity of the detection procedure [193,194], and the same incubation temperature throughout the reaction, and hence more potential for portable device development [195]. Electrochemical-based methods, such as cyclic voltammetry [113], differential pulse voltammetry [114,117,118], square-wave voltammetry [119], electrochemical impedance spectroscopy [112,116], and chronoamperometry [110], are emerging as inexpensive detection techniques with point-of-care applications. The average diagnostic accuracy for eight electrochemical and electrochemiluminescence clinical studies was 94%. The clinical performance of electrochemical methods is lower than that of conventional virus and bacteria detection techniques, such as PCR and LAMP. Nevertheless, electrochemical-based methods can be regarded for the rapidity of the detection, simplicity of use (automation), low cost, and portability that can be useful in mass testing [196,197,198]. One of the issues with electrochemical sensors is the limited number of reported applications of multiplexing; for this reason, multiple biorecognition elements on a single electrode should be designed [198]. The average accuracy of the clinical diagnosis using ELISA and Raman/SERS was 95% and 99%, respectively. ELISA, electrochemical, Raman/SERS techniques are frequently used in the detection of cancer, non-cancer biomarkers, and toxins, and therefore, are more extensively discussed later in the review.

## 3. Cancer and Non-Cancer Biomarker Detection

Cancer is a group of diseases characterized by pathologically rapid and uncontrolled growth of atypical (mutated) cells, which spread through muscles and organs, eventually affecting the entire body. Cancer is caused by genetic mutations in DNA, which usually affect pro-oncogenes, tumor suppressor genes, and genes responsible for DNA repair. Currently, more than 100 types of cancer are known, and the most common among them are breast, prostate, lung, rectal, and colon cancers [199,200]. According to the World Health Organization (WHO), cancer is the second leading cause of death worldwide [201]. Despite the remarkable progress achieved in cancer treatment in the last decade, the decrease in the mortality rate due to cancer is insignificant [55]. For instance, the overall cancer death rate in the USA from 2001 to 2020 decreased by less than 2%, which is still a positive development, but there is definitely a place for improvement [56,202]. The cancer mortality rate worldwide was estimated at 100 deaths per 100,000 population [203]. According to global cancer statistics published in 2021, 19.29 million new cases of cancer and 9.96 million cancer deaths are registered annually [204]. The stage at which cancer is diagnosed is one of the most important predictors of survival. The 5-year survival rate decreases sharply in patients with advanced stages of cancer in comparison with those with localized disease [205]. For lung cancer, for example, the 5-year survival rates for localized, regional, and distant stages are 59%, 32%, and 6%, respectively [205]. Therefore, early detection of cancer is vital since it significantly affects the survivability of patients. For one of the strategies for early cancer detection, namely the mutation-based sequencing approach, the low limit of detection is particularly important. From the multiple processes, including DNA extraction, target enrichment, library preparation, sequencing, etc., a background noise can originate, which eventually leads to false positive results [206].

Although cancer has always been one of the leading causes of lethal outcomes all around the world, there is a non-cancer series of diseases that lead to a larger number of deaths annually than all types of cancer—cardiovascular diseases. According to the National Center for Health Statistics, heart disease was the leading cause of death in 2020 and 2021 in the United States, contributing to 168 and 174 deaths per 100,000 U.S. standard population for these two years, respectively. This is slightly higher than the contribution of cancer to U.S. mortality (144 and 147 in 2020 and 2021, respectively) [207]. Heart failure, which is considered one of the main representatives of this type of disease, is a sophisticated clinical condition that is characterized by a decline in the heart’s ability to fill or pump out blood caused by a wide set of cardiovascular disorders. Over 26 million people worldwide are affected by this disease. This number continues to grow with the aging of the population [208].

Alzheimer’s disease is a progressive, irreversible brain condition that is characterized by advancing memory degradation and deterioration of cognitive abilities [209]. As Alzheimer’s disease progresses, it eventually leads to dementia [210]. Around 37 million people worldwide suffer from dementia, and 17 million cases are caused by Alzheimer’s disease, meaning it is the most prevalent cause of dementia [209]. It is estimated that 6.7 million people aged 65 and older in the United States are diagnosed with Alzheimer’s disease [211]. A total of 121 thousand lethal outcomes from Alzheimer’s disease were registered in 2019 in the USA, and this brain disorder was the seventh leading cause of mortality in the USA in 2020 and 2021 [207,211]. The mortality rate is estimated at 32 and 31 deaths per 100,000 for 2020 and 2021, respectively [207]. These numbers are several times smaller than the corresponding statistics for heart failure and cancer; however, it is still quite substantial, especially because most of the affected are elderly people [207].

Biomarkers are substances or biological parameters that are used to monitor and determine changes in the human organism [212]. Biomarkers are usually present in different biofluids, such as serum, urine, and saliva. Serum is often used as a reliable medium for biomarker detection; however, it has a significant disadvantage of being invasive. Saliva is a complex biological fluid that consists of 98% water and 2% of other compounds [213]. Some of these compounds can be used as biomarkers for the detection of numerous diseases, including many types of cancer, cardiovascular diseases, Alzheimer’s disease, and other diseases. As was already mentioned, these diseases are very lethal with decreasing survivability as the illness progresses. Due to that, there is a need for a sensitive analytical method that is suitable for the early diagnosis of the above-mentioned clinical conditions. This section of the review focuses on the analysis and comparison of popular analytical techniques, such as ELISA, mass spectrometry, electrochemical methods, and SERS, for non-invasive detection of cancer and non-cancer biomarkers in saliva.

### 3.1. Mass Spectroscopy

Proteomics is a field that focuses on studying proteins, including their structures and functions, on a large scale. This area is evolving quickly by merging advanced analytical equipment with sophisticated computational techniques to achieve a complete understanding of the proteome of a biological system or organism. Mass spectrometry (MS) has become the preferred technique for identifying and characterizing proteins, leading to the development of high-throughput proteomics, which involves the large-scale investigation of protein expression and function [214].

Mass spectrometry has a long-standing history in protein analysis, with the initial application of MS to the analysis of proteins dating back to the 1950s. One significant development in this field was in 1959, when Fred W. McLafferty and his colleagues employed mass spectrometry to determine the molecular weight of the protein myoglobin [215]. Another major advancement occurred in the 1970s when Franz Hillenkamp and Michael Karas introduced matrix-assisted laser desorption/ionization (MALDI) for soft ionization of large biomolecules. This innovative technique enabled the study of intact proteins and paved the way for further developments in the field of MS-based proteomics [216]. In recent years, MS-based proteomics has become widely adopted in various laboratories and has facilitated numerous biological and clinical investigations [214].

Mass spectrometry-based proteomics is an expensive and time-consuming process that requires specialized equipment, trained personnel, and costly reagents and consumables [217]. For example, the price of mass spectrometers can range from $10,000 to $1 million [218]. Additionally, the analysis of large sample sets generates massive amounts of data that necessitate advanced computational resources for analysis and processing [217]. Also, mass spectrometry-based proteomics has some limitations, such as low sensitivity for low-abundance proteins [219]. Furthermore, the MS method faces difficulties in analyzing membrane proteins because of their hydrophobicity and low abundance [220]. Despite these drawbacks, it remains a powerful method for detecting and quantifying proteins and peptides. New mass spectrometers used in proteomics have excellent resolving power, mass measurement accuracy, and sequencing speed [221]. MS has allowed for the identification of numerous new proteins and their modifications after translation and has resulted in greater knowledge of protein–protein interactions, protein networks, and signaling pathways. The use of high-precision and high-resolution mass spectrometry has made it possible to identify and quantify proteins and their modifications [217,221].

In this section, we have provided examples of detecting cancer biomarkers in saliva using MS methods. Human saliva is a valuable biological fluid for diagnostic purposes due to the noninvasive sampling method [222]. High-throughput proteomic approaches have identified over 3000 proteins and peptides with differing expression levels in saliva [223]. Eleven analytical studies are summarized in Table 3. Analysis of the limit of detection (LOD) for each biomarker revealed that the immuno-MALDI-MS method achieved the lowest LOD of 2.6 × 10^−16^ M when detecting the oral cancer biomarker MMP1 [37]. Calculated geometric average and median of LOD for the reported in 14 impactful publications in the last decade were 7.05 × 10^−10^ M (mol L^−1^) and 1.18 × 10^−9^ M (mol L^−1^), respectively. However, the range of reported LOD scans a few orders of magnitude from 2.6 × 10^−16^ M to 1.79 × 10^−6^ M.

One of the major MS techniques in proteomics is tandem mass spectrometry (TANDEM MS or MS/MS), which has simplified the separation of biological compounds in various biomedical research experiments [234]. Tandem MS is a two-stage technique that can be carried out using either multiple mass spectrometers or one with multiple analyzers. It typically consists of two or three quadrupoles and a TOF analyzer and is particularly useful for analyzing complex mixtures. In the first stage, a specific set of ions is isolated and broken down into fragments by a chemical reaction. In the second stage, mass spectra are created for these fragments [235]. For instance, electrospray ionization in Liquid Chromatography with tandem MS (LC-MS/MS) allows for the online separation and identification of delicate molecules. LC-MS/MS offers superior selectivity and sensitivity in the quantification of targeted compounds due to its mass-specific detector, which is independent of a particular functional group [234]. Grau et al. developed a new methodology using LC-MS/MS to measure cortisol and cortisone in the M-SA-DSPE extract. The insight is to use M-SA-DSPE, a magnetic sorbent like in M-DSPE, and a disperser solvent like in SA-DSPE. The magnetic sorbent containing the target analytes is recovered with an external magnet, and the analytes are desorbed into an organic solvent for subsequent chromatographic analysis. M-SA-DSPE involves using CoFe_2_O_4_ magnetic nanoparticles embedded into a reversed-phase polymer as a sorbent, and LC-MS/MS is used to measure analytes in the extract. The method’s optimal conditions resulted in favorable analytical characteristics, such as low limits of detection for cortisol and cortisone at 0.029 ng mL^−1^ and 0.018 ng mL^−1^, respectively, and a repeatability of ≤ 10% as RSD. Relative recoveries were between 86 and 111%. This approach enables quick and non-invasive detection of cortisol and cortisone, utilizing only small quantities of sample, organic solvent, and sorbent. The sample preparation requirements are minimal, as the method does not require supporting equipment [229].

Selected reaction monitoring (SRM) is a targeted mass spectrometry technique that detects and quantifies specific proteins in a complex background. It is commonly used to study signaling pathways and involves selecting unique proteotypic peptides to represent the protein of interest. SRM is performed on a triple quadrupole mass spectrometer and has several advantages, including a lower limit of detection, reduced noise levels, near-absolute structural specificity for the target protein, and the ability to accurately quantify target proteins in complex mixtures using stable-isotope dilution [236]. For example, Kawahara et al. developed the technique of selected reaction monitoring (SRM) tandem mass spectrometry in their research. The study found that OSCC patients had higher levels of certain biomarkers, including C1R, LCN2, SLPI, FAM49B, TAGLN2, CFB, C3, C4B, LRG1, and SERPINA1. Moreover, their study found an association between CFB, C3, C4B, SERPINA1, and LRG1 biomarkers and the risk of developing oral squamous cell carcinoma (OSCC). Targeted proteomic approach is now recognized as a highly precise and quantitative method for analyzing peptides in complex biological samples [226].

**Figure 3:**
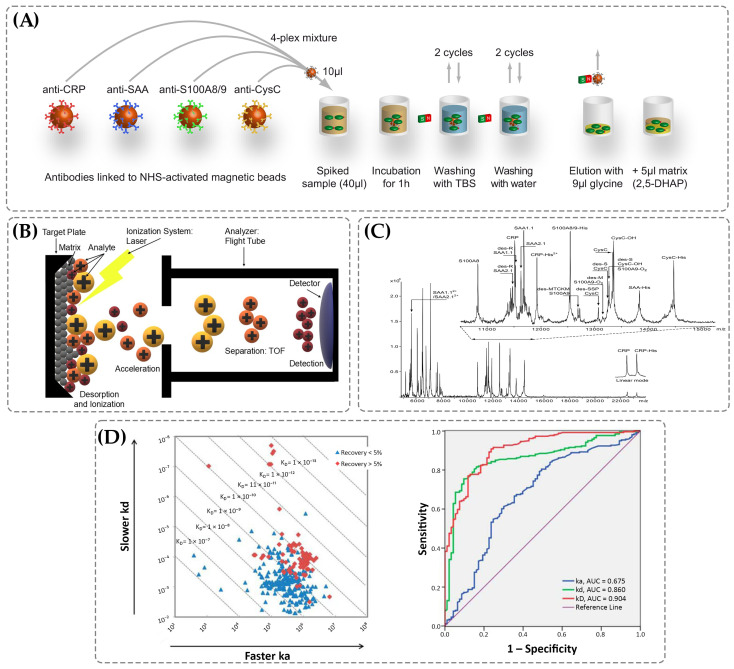
Study of cancer identification by immuno-matrix-assisted laser desorption/ionization time-of-flight (Immuno-MALDI-TOF) mass spectrometry from human biofluid, including saliva. (**A**) Immuno-MALDI-TOF MS assay workflow. Adapted with permission from Gao et al. [237] Copyright © 2018, American Chemical Society. (**B**) Illustration of the MALDI-TOF mass spectrometer. Adapted with permission from Bartusik-Aebisher et al. [238] Copyright ©2025 by the authors. Licensee MDPI, Basel, Switzerland. This article is an open-access article distributed under the terms and conditions of the Creative Commons Attribution (CC BY) license. (**C**) Mass spectra of the sample. Adapted with permission from Gao et al. [237] Copyright © 2018, American Chemical Society. (**D**) Statistical analysis. Adapted from Hsiao et al. [227], licensed under CC BY 4.0. Copyright © 2017 Elsevier B.V.

A technique called immuno-enrichment with matrix-assisted laser desorption/ionization (immuno-MALDI) based MS has been developed, which enables rapid and accurate analysis of many samples by combining high sensitivity and specificity. Hsiao et al. developed immuno-enrichment with matrix-assisted laser desorption/ionization time-of-flight MS (Immuno-MALDI-TOF-MS) technique to detect matrix metalloproteinase-1 (MMP1) in human saliva. The methodology showed good accuracy, with intraday and inter-day variations in less than 10%, and a low limit of detection (LOD) at 0.26 fmol, and a limit of quantification (LOQ) at 3.07 ng mL^−1^ [37]. To identify cancer using Immuno-MALDI-TOF mass spectrometry from human saliva, the initial step involves preparing the clinical saliva samples. After the appropriate preparation of samples, intact proteins were captured from native samples (Figure 3). The samples were spiked with internal standards and incubated with antibody-conjugated magnetic beads. Researchers used automated systems for working with magnetic particles to perform particle transfer, binding, washing, and elution. Magnetic probes are used to transfer particles with immobilized antibodies to plated with samples and internal incubation standards. After washing, the analytes were eluted with chemicals for proteins, mixed with matrix, spotted onto a plate, and analyzed using a MALDI-TOF mass spectrometer. This approach can be used for a variety of biological samples, including saliva, even though Gao et al. used plasma samples [237].

A schematic diagram of the MALDI-TOF mass spectrometer, which was used to quantify the concentration of the analyte, is shown in Figure 3B. The sample is ionized by a laser pulse, after which the ions are accelerated and separated in a time-of-flight tube based on their mass-to-charge ratio before detection [238]. This method yields a spectrum that identifies proteoforms by their mass-to-charge ratio and quantifies them by peak intensity (Figure 3C) [237]. The data were analyzed and presented using statistical analysis and ROC curves to validate the method and biomarker performance (Figure 3D) [227]. With the help of Hamilton automation technology, the immuno-MALDI analysis can be used for high-volume screening. An immuno-enrichment step before the MALDI-TOF analysis enables the selective capture of the analyte from complex samples, eliminates background interference, and improves detection sensitivity. By combining these two features, the workflow of such studies aims to enhance molecular diagnostic tests using biomarkers [37,237].

Using multiple reaction monitoring (MRM) mode with a triple quadrupole MS enables the selection of specific precursors and product ions that correspond to targeted compounds, thereby reducing noise and screening out other ions. As a result, MRM is widely utilized for accurate, rapid, sensitive, and multiplexed quantification of targeted compounds in complex mixtures [239]. For example, the multiplexed LC-MS/MS method offers several advantages over other methods, such as the ability to analyze numerous salivary proteins in a single run, enabling marker panel development, identifying all proteins in a single run, with SIS peptides providing more accurate quantitation data than immunoassays, and requiring only small sample volumes, making it useful for clinical samples with limited amounts. Chen et al. developed a highly sensitive and precise platform using LC-MRM/MS analysis to quantify 56 proteins in saliva. For this study, four protocols were used for the enhancement of tryptic digestion of proteins. Results of the analysis showed that the third procedure, with the addition of a surfactant, is a preparation technique with the best accuracy. Also, digestion efficiency is optimized by adding a surface-active agent. The authors optimized the analytical workflow, assessed technical and clinical performances, and determined LLOQ and LOD values for each protein. They discovered that several targeted proteins were elevated in OSCC saliva specimens, suggesting that they could be potential biomarkers for oral cancer screening. The LLOQ values for endogenous salivary proteins ranged from 2.6 amol µg^−1^ for APOB to 7.4 fmol µg^−1^ for ZA2G, while LOD values ranged from 0.1 amol µg^−1^ for KNG1 to 5628.0 amol µg^−1^ for A1AG1 [228].

As proteomics MS-based platforms become more selective, have higher resolution, and greater sensitivity in the future, it will be possible to conduct more detailed investigations on proteomes from diverse sources [223]. The application of quantitative mass spectrometry techniques, specifically MRM, has emerged as the future of salivary research. This approach allows for more accurate and efficient analysis of saliva samples and can be implemented in clinical settings for routine measurements and the development of new clinical tests [222].

Scientists are investigating the use of MS to identify cancer biomarkers in saliva, and some studies have found hopeful outcomes for different types of cancer, such as oral, breast, and lung cancer.

The advantages of the tandem MS technique over other analysis methods mentioned earlier have been described. For example, due to the distinctive fragmentation patterns of compounds, the LC-MS/MS method enables more precise and selective simultaneous analysis compared to the ELISA method [240]. Calculated median LOD of all biomarkers in saliva was 1.18 × 10^−9^ M (mol L^−1^). It is essential to attain a low LOD to precisely and sensitively quantify specific proteins present in saliva, particularly for clinical purposes like the identification of cancer biomarkers, early diagnosis of diseases, and treatment. Still, more research is needed to standardize the methods of collecting, processing, and analyzing samples, and to verify the identified biomarkers in a diverse population.

### 3.2. ELISA, Immunoassays

Immunoassay takes use of the specific interaction between a key protein reagent, the antibody, and a target molecule–originally an antigen, but also can be small organic compounds, or even a microorganism [241]. There are various detection techniques immunoassays are based on, and one of the most popular ones is the enzyme immunoassay and enzyme-linked immunosorbent assay (ELISA) introduced by Engvall and Perlmann [242], van Weeman and Schuurs [243] in 1971 [244,245].

In comparison to other immunoassays, ELISA can provide reliable, sensitive, and specific detection results. Moreover, ELISA is cost-effective, demonstrates good specificity and robustness, and can be applied for high-throughput analysis [246,247]. ELISA has been used for various applications, such as the detection of viruses [155], bacteria [154], food contaminants [248], toxins [249], biomarkers [250], cancer markers [39], etc. The detection of cancer and non-cancer biomarkers using ELISA and other immunoassay-based techniques is demonstrated in Table 4.

The multiplex detection of nine biothreat toxins is shown in Table 4 by using protein microarray technology, the miniaturized version of the traditional ELISA performed by Jenko et al. [249]. The average LOD in saliva was about one picomolar (median was 0.9 pM) with a 12 h assay time. LOD for toxins was in the same order of magnitude as other biofluids: serum, nasal, urine, and plasma samples in general. A little cross-reactivity of less than 2% was observed among all ten toxins, thus demonstrating the applicability of ELISA in the simultaneous screening of multiple antigens and other targets [249].

**Figure 4:**
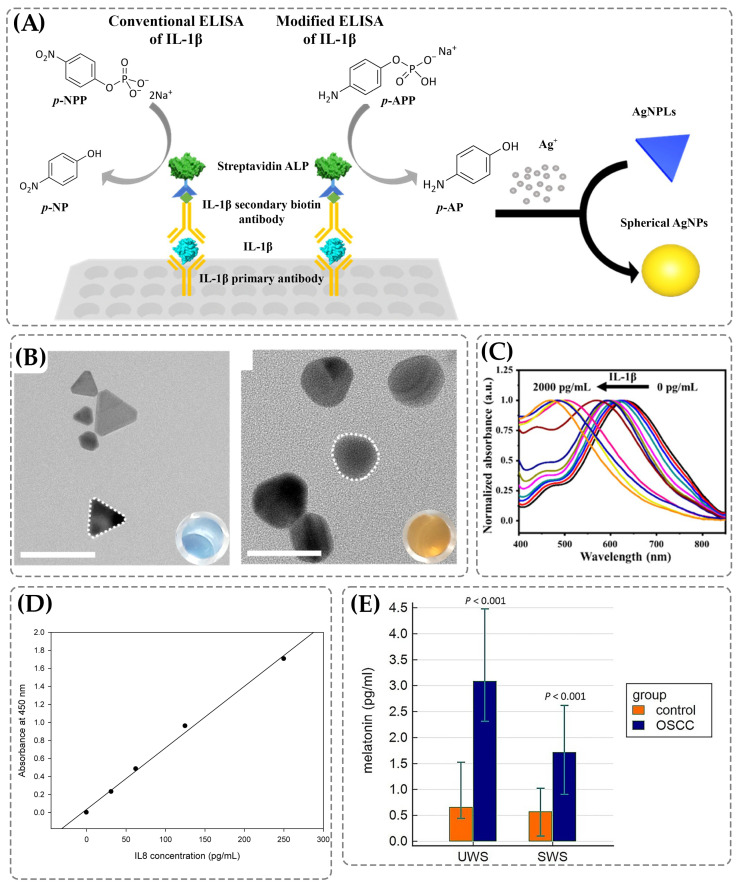
Detection of biomarkers in saliva using the enzyme-linked immunosorbent assay (ELISA) technique. (**A**) Illustration of conventional ELISA and silver triangular nanoplates (AgNPL)-integrated ELISA for interleukin-1β detection. (**B**) Transmission electron microscopy (TEM) images of silver triangular nanoplates functionalized with alkaline phosphatase (ALP) (concentration increased from 0 to 250 U L^−1^). (**C**) Ultraviolet–visible spectra of nanoplates at various concentrations of interleukin-1β in the modified ELISA. Adapted with permission from Kim et al. [257]. Copyright © 2023 American Chemical Society. (**D**) Calibration curve for IL-8 in buffer using a Duoset ELISA kit. Adapted from Haliru et al. [262] © 2026 The authors. Published by the American Chemical Society. This publication is licensed under CC-BY4.0. (**E**) Comparison of biomarker levels (pg mL^−1^) in saliva between the control group and the OSCC group. Adapted from Salaric et al. [39], licensed under CC BY 4.0. Copyright © 2021 Nature Publishing Group.

Kim et al. developed a modified ELISA to detect interleukin-1 beta (IL-1β) and alkaline phosphatase in saliva [257]. The scheme of modified ELISA in comparison with the traditional ELISA is provided in Figure 4A, where the multicolor silver triangular nanoplates (AgNPL) sensor was integrated with the traditional single-color ELISA technique. The scheme of modified ELISA in comparison with the traditional ELISA is provided in Figure 4A, where the multicolor silver triangular nanoplates (AgNPL) sensor was integrated with the traditional single-color ELISA technique. Silver nanoplates are structurally and chemically stable in biofluids and have high LSPR sensitivity and stronger plasmon interaction with light than other metal particles. Therefore, AgNPL was used as a biosensor and optical transducer. The enzymatic activity of alkaline phosphatase (ALP) was used to hydrolyze p-nitrophenol phosphate (p-NPP) to p-nitrophenol (p-NP). The localized SPR properties of AgNPL were changed by ALP. As a result, the concentration of Ag^+^ on the surface of AgNPL was reduced, and the controlling seed on the active boundary of the silver NPLs was increased. This leads to the shape transformation of nanoplates from triangular to spherical and color changing from blue to yellow, which allows to determine target concentrations of ALP. The growing concentration of ALP leads to the deposition of silver atoms on the AgNPL and the LSPR shift in AgNPL.

Figure 4B demonstrates TEM images of transformed ALP with two different concentrations. Kim et al. observed a progressive blue shift in the LSPR in UV–Vis spectra of AgNPL, approximately from λ_max_ 620 nm to λ_max_ 460 nm, with IL-1β levels ranging from 0 to 2 ng mL^−1^ (Figure 4C). Silver triangular nanoplates-integrated ELISA showed the lowest LOD for ELISA reported in this review, with 0.066 pg mL^−1^ for IL-1β in real human saliva (3.77 × 10^−15^ M) and 0.0011 U L^−1^ for ALP. In comparison, the conventional ELISA method achieved the LOD value of 3.8 pg mL^−1^ when detecting IL-1β [257].

Salaric et al. [39] and Chakraborty et al. [41] employed ELISA on saliva samples for the diagnosis of oral cancer. The incorporation of gold nanorods or gold nanospheres into the traditional ELISA resulted in improved sensitivity and lowered LOD in the study of Chakraborty et al. [41]. The LODs of 0.02 ng mL^−1^ (about 0.5 pM) with gold nanorods and 0.03 ng mL^−1^ (0.7 pM) with gold nanospheres were reported, while the LOD attained with the conventional ELISA was 0.14 ng mL^−1^. Calibration curves for protein IL-8 were obtained using the ELISA presented in Figure 4D. Measurements were performed in the saliva of oral lichen planus and healthy patients [262]. It shows a highly linear response (R^2^ = 0.99) in the range 0.313 ng mL^−1^ to 20 ng mL^−1^. The graph provided in Figure 4E shows a comparison of the levels of melatonin in saliva using ELISA kits, measured in pg mL^−1^, between the healthy group and the group diagnosed with oral squamous cell carcinoma [39].

The average and median detection limit for ELISA and other immunoassay studies provided in Table 4 are 1.20 pM and 0.86 pM, respectively; the range is from nano to femtomolar. ELISA, as a detection tool for saliva samples, demonstrated sensitivity down to the picomolar range and applicability in the detection of toxins and biomarkers for various disorders. Traditional ELISA may have a relatively low sensitivity [247,263]; nevertheless, the sensitivity of ELISA can be comparatively increased by employing gold or silver nanoparticles to amplify the signals [41,257]. Other limitations of the traditional 96-well ELISA include the long assay time of 4–12 h, high cost, large sample-to-reagent consumption, and the limited reported applications of lab-on-chip devices [264,265]. The enzyme label can lose catalytic activity due to heat, pH, or chemical-induced denaturation [266]. Furthermore, even the slightest denaturation of antibodies may expose hydrophobic regions, thereby resulting in non-specific binding [267].

### 3.3. Electrochemical Detection

Table 5 presents 38 studies based on the electrochemical methods of detection of the salivary cancer and non-cancer biomarkers. Among them, 14 are based on voltammetry, 14 on electrochemical impedance spectroscopy (EIS), 8 on amperometry techniques, and 2 on other electrochemical techniques. The median limit of detection (LOD) for all the above studies and techniques was 5.5 × 10^−13^ M. Analyzing the studies, electrochemical sensors that were used in electrochemical impedance spectroscopy are found to be more sensitive, with the smallest geometric mean and median values of LOD. They were, respectively, equal to 1.96 × 10^−14^ M and 2.35 × 10^−15^ M.

Electrochemical impedance spectroscopy (EIS) is a method used to measure alternating electric current that passes through materials and determine the dispersion of complex resistance [300]. EIS is used in studies of mass and charge transfer and diffusion. It also helps to investigate the factors that may affect the conductivity, resistance, or capacitance of the electrochemical system [300]. EIS is a very sensitive method, as it has a number of advantages. This method is non-destructive and operational, since it is possible to obtain data that depends on time. It can be used in the study of materials with high resistance and in service environments. As a result of the research, quantitative data is obtained, which is convenient to analyze and interpret [301].

Choudhary et al. presented the most sensitive electrochemical immunosensor with EIS [40]. This study was based on the detection of CD-59, and the LOD of this sensor was 1.0 × 10^−16^ M. In addition to the low detection limit, this method has good sensitivity (94%) and a detection time of 10 min. The most sensitive sensor, among all the research presented in Table 5, turned out to be an immunosensing device with voltammetry developed by Farzin et al. [47]. The LOD in this work was 8.4 × 10^−17^ M [47]. This sensor is designed for prostate-specific antigen (PSA). The amplified nanoplatform used in this study makes it possible to accelerate electron transfer, which in turn makes it possible to use this platform to analyze complex compounds of real samples. The median for the LOD of voltametric methods was 5.5 × 10^−13^ M. The median LOD in works with amperometry was 8.5 × 10^−13^ M. This method is more sensitive than voltammetry. In addition to the above categories, Table 5 presents methods based on photolithography and electrochemical deposition methods, and electrochemiluminescence (ECL) for the detection of salivary cancer biomarkers [298,299]. The average LOD value for these techniques was 4.4 × 10^−12^ M. It is important to note that the number of papers presented in each category differs, since in cancer biomarker studies, cyclic and differential potential voltammetry are more often used than EIS, amperometry, and other techniques. In this regard, for a full-fledged analysis, an equal number of studies presented in each category should be used. In addition to this LOD, response time also plays a significant role in evaluating analytical methods. Since there is a response time only in a limited number of works, it is impossible to rationally analyze techniques based on this factor. In addition, it can be noted that interleukin-8 (IL-8) and cytokeratin-21-1 fragment (CYFRA-21-1) are the most frequently found analytes among these methods.

**Figure 5:**
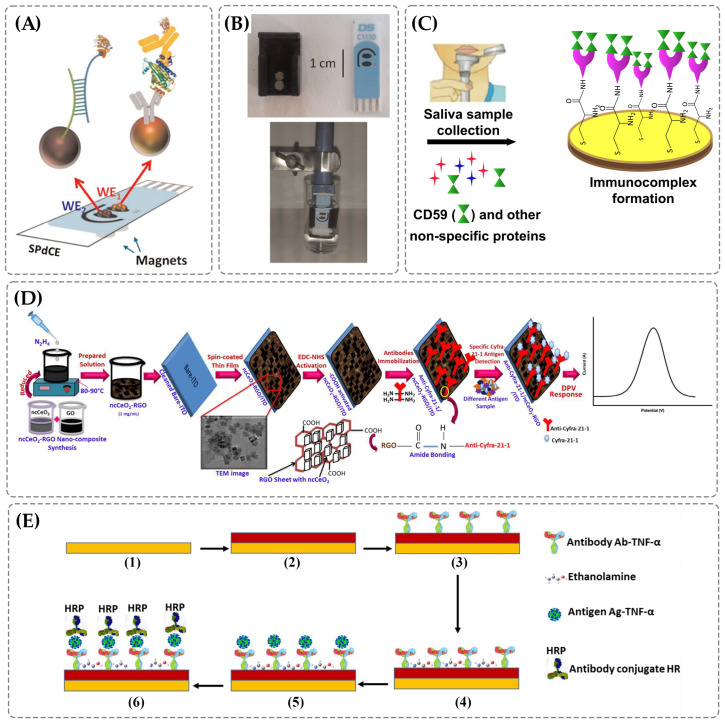
Detection of salivary cancer biomarkers using amperometry and electrochemical impedance spectroscopy (EIS) techniques. (**A**) Schematic representation of the development of a disposable dual magnetobiosensor for the determination of interleukin-8 (IL-8) protein and IL-8 mRNA. (**B**) Relative size of the components of the disposable dual magnetobiosensor for IL-8 protein and IL-8 mRNA detection. Adapted with permission from Torrente-Rodriguez et al. [291]. Copyright © 2016 Elsevier B.V. (**C**) Schematic representation of the interaction mechanism of a fabricated oral cancer biosensor for the detection of the CD-59 biomarker. Adapted with permission from Choudhary et al. [40]. Copyright © 2016 John Wiley & Sons. (**D**) Fabrication of BSA/Anti-Cyfra-21-1/ncCeO2-RGO/ITO electrochemical immunosensor. Reproduced with permission from Pachauri et al. [271]. Copyright © 2018 Royal Society of Chemistry. (**E**) Schematic fabrication of the chronoamperometric immunosensor. Reprinted with permission from Barhoumi et al. [292]. Copyright © 2018 Elsevier B.V.

Electrochemical Impedance Spectroscopy and amperometry were used due to their high sensitivity and accuracy in determining various cancer and non-cancer biomarkers in saliva, as shown in Figure 5. The fabrication of a disposable dual magnetobiosensor for the determination of IL-8 protein and IL-8 mRNA is presented in Figure 5A. Dual screen-printed carbon electrodes (SPdCE) presented by Torrente-Rodriguez et al. are constructed from a homemade magnet holding block in the upper part and the modified MBs placed on this block in the specific cable connector [291]. Sensors presented by Torrente-Rodriguez et al., used to detect both proteins and markers of genetic nature, can be used to determine not only cancer biomarkers, but also markers of other diseases. Figure 5B represents the relative size of the components of the SPdCE, which were drawn not on their real scale. The interaction of Au/Cys/Anti-CD 59 with the saliva sample is shown in Figure 5C. This sensor was fabricated using an Au electrode functionalized by L-Cysteine. This functionalization was made to provide carboxyl functional groups. Then, the anti-CD-59 antibodies were immobilized onto the electrode. After interaction with saliva samples, an impedimetric response for the detection of the CD-59 biomarker was performed [40]. Figure 5D demonstrates an electrochemical immunosensor built by Pachauri et al. on a cerium oxide nanocube–reduced graphene oxide (ncCeO_2_-RGO) nanocomposite for detecting Cyfra-21-1, an oral cancer biomarker in saliva. The nanocomposite was synthesized at low temperature and spin-coated onto ITO glass, with Anti-Cyfra-21-1 antibodies immobilized via EDC-NHS chemistry. Using differential pulse voltammetry, the sensor achieved an exceptionally low detection limit of 0.625 pg mL^−1^, a sensitivity of 14.54 µA ng^−1^ mL cm^−2^, and a wide detection range up to 15 ng mL^−1^, outperforming previously reported Cyfra-21-1 immunosensors [271]. Barhoumi et al. presented the fabrication of a chronoamperometric sensor illustrated in Figure 5E [292]. First of all, a bare gold working electrode (WE) characterized with cyclic voltammetry was presented. Voltammetry was also applied to activate 4-carboxymethylaniline (CMA), followed by CMA electrodeposition on gold WEs. Before the activation of carboxylic acid groups (COOH), the working electrode was stored in the electrochemical cells. Gold WE were washed with water and dried with nitrogen. COOH-groups inside CMA were activated by incubation in an ethanolic solution of EDC (0.4 M)/NHS (0.1 M) at room temperature for 1 h. To remove the excess of EDC/NHS, hydrochloric acid was used. Afterward, the working electrode was incubated in PBS containing Ab-TNF-α. Incubation in ethanolamine solution was performed to deactivate remained active carboxylic acid groups.

The incubation of the biosensor in a standard solution with different Ag-TNF-alpha concentrations was performed for the detection of cytokines. Incubation was carried out several times at 4 °C for 30 min. To finish the electrode fabrication, the immunosensor was washed using PBS solution and then incubated in 2 µg mL^−1^ of Ag-TNF-α-HRP using the same conditions as in the previous incubation [292]. The overall fabrication scheme is shown in Figure 5E. The biosensor fabrication proceeds through six sequential stages. Beginning with (1) the unmodified gold electrode as the base substrate, (2) a layer of 4-carboxymethylaniline (CMA) is then electrochemically grafted onto the surface, with its carboxyl groups subsequently activated for further conjugation. In stage (3), anti-TNF-α capture antibodies are immobilized onto this activated CMA layer, followed by (4) a blocking step using ethanolamine to passivate any remaining unreacted carboxyl groups and minimize nonspecific adsorption. The functionalized electrode is then (5) incubated with the target sample, allowing TNF-α antigen to bind selectively to the anchored antibodies. Finally, in (6), HRP-conjugated secondary anti-TNF-α antibodies are introduced and bind to the captured antigen, completing the sandwich immunocomplex.

Tumor necrosis factor-α (TNF-α) is a pro-inflammatory cytokine that acts as a central mediator of a wide range of biological activities. The improper balance of TNF-α may be an indicator of a certain immune or inflammatory-related disease. In other words, TNF-α is considered a biomarker for various diseases, such as heart failure, cancer, Alzheimer’s, etc. Heart failure is of special importance among these, since it contributes the most to mortality in Western countries [293]. Detection of TNF-α and similar heart failure biomarkers in saliva offers some substantial advantages in comparison to serum detection, i.e., it is non-invasive, painless, has a potential for real-time monitoring, etc. [280].

**Figure 6:**
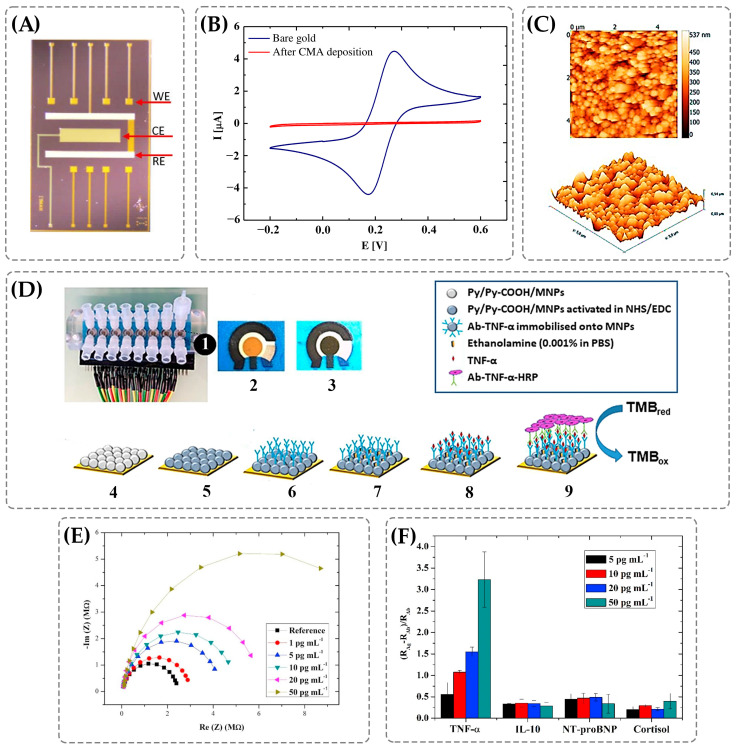
(**A**) Biosensor platform with eight gold working electrodes (WE), an Ag/AgCl reference electrode (RE), and a platinum counter electrode (CE). (**B**) Cyclic voltammograms of gold before (I) and after (II) CMA deposition. Reprinted with permission from Bellagambi et al. [280]. Copyright © 2017 Elsevier B.V. (**C**) Gold screen-printed electrode (SPEAu) after electrodeposition of Py/Py-COOH/MNPs in 2D (top) and 3D (bottom). (**D**) Schematic illustration of the preparation and detection approach of the reported immunosensor. Adapted from Barhoumi et al. [293], licensed under CC BY 4.0. Copyright © 2019 Elsevier B.V. (**E**) Representative Nyquist plots (obtained from fitting each spectrum to the proposed equivalent circuit model) for TNF-α standard solutions in PBS (1, 5, 10, 20, and 50 pg mL^−1^). (**F**) Results of the interference study obtained by analyzing standard solutions containing TNF-α or other heart failure (HF) biomarkers in the concentration range of 5–50 pg mL^−1^ using the ImmunoFET functionalized with mAbTNF-α. Adapted with permission from Ben Halima et al. [283]. Copyright © 2021 Elsevier B.V.

One of the most prevalent analytical techniques for the detection of TNF-α in saliva is different electrochemical methods, such as voltammetry, amperometry, and impedance spectroscopy. Bellagambi et al. in 2017 performed the detection of TNF-α in human and artificial saliva using electrochemical impedance spectroscopy (EIS) [280]. The bio-functionalization of a biosensor platform they used for the detection of TNF-α consisted of several steps. Firstly, they linked 4-carboxymethylaniline (CMA) molecules onto gold working electrodes at the biosensor platform (Figure 6A) through application of the cyclic voltammetry method. Figure 6B demonstrates the cyclic voltammogram for gold WE carried out in K_3_[Fe(CN)_6_]/K_4_[Fe(CN)_6_] (5 mM) in PBS (pH 7.4) before (blue) and after (red) deposition of CMA molecules. The CMA blocking layer has caused the weak electron transfer kinetics of K_3_[Fe(CN)_6_]/K_4_[Fe(CN)_6_], so that there are no oxido-reduction peaks in the cyclic voltammogram after CMA deposition. After the CMA deposition, the carboxylic acid groups of CMA were activated through incubation in EDC/NHS in ethanol solution. The excess EDC/NHS was removed by washing with HCl, after which the device was promptly treated with PBS solution containing anti-TNF-α antibodies. The deactivation of the remaining carboxylic acid groups was then performed by Bellagambi et al. [280] using ethanolamine solution, which is needed to minimize nonspecific binding during detection. Consequently, this biofunctionalized biosensor platform was used to detect TNF-α in PBS solution, in artificial saliva, and most importantly in real human saliva. As a result, Bellagambi et al. obtained the concentration of 3.1 pg mL^−1^ for TNF-α in real saliva, which can also be referred to as the limit of detection of their EIS measurements. This concentration is comparable to the level of TNF-α in the saliva of nominally healthy subjects, which means that this method is suitable for quantification of TNF-α in saliva for HF diagnosis. In addition to that, the method reported by Bellagambi et al. has shown a good selectivity in the presence of other cytokines, such as interleukin-1 (IL-1) and interleukin-8 (IL-8), which corresponds to an insignificant susceptibility to nonspecific binding.

In another research made in 2019, Barhoumi et al. used the method of chronoamperometry to detect TNF-α in artificial saliva with high sensitivity [293]. The immunosensor array that they used in their study was based on a screen-printed gold electrode (SPEAu). Barhoumi et al. applied two different SPEAu functionalization strategies, one with 2D-SPEAu, the functionalization of which was very similar to that used by Bellagambi et al. in 2017, and the other with 3D-SPEAu, which was functionalized by attaching magnetite magnetic microparticles (MMPs) coated with poly(pyrrole-co-pyrrole-2-carboxylic acid) (Py/Py-COOH) (AFM images of which are shown in Figure 6C) and consequent binding of anti-TNF-α antibodies (Ab-TNF-α) [280]. Figure 6D demonstrates the preparation process for the immunosensor used by Bellagambi et al.: flow cells for 8X format (Figure 6(D1)), stereo-microscopic image of bare SPEAu (Figure 6(D2)), and stereo-microscopic image of SPEAu modified by electrodeposition of Py/Py-COOH/MNPs (Figure 6(D3)). During the preparation of 2D-SPEAu, Py/Py-COOH/MMPs were first attached onto SPEAu via the method of pulsed chronoamperometry (Figure 6(D4)). Then the carboxylic acid groups at Py/Py-COOH/MMPs were incubated in an ethanolic solution of EDC/NHS (Figure 6(D5)). After that, Ab-TNF-α were immobilized onto the MMPs surface (Figure 6(D6)), and the SPEAu was then treated with the solution of ethanolamine to deactivate free carboxylic acid groups (Figure 6(D7)). Consequently, TNF-α was captured from one side by Ab-TNF-α (Figure 6(D8)) and from another side by antibody anti-TNF-α labeled with horseradish peroxidase (Ab-TNF-α-HRP) (Figure 6(D9)). Finally, the obtained 3D-SPEAu immunosensor array was used to detect TNF-α. Barhoumi et al. achieved an LOD value of 0.3 pg mL^−1^ for TNF-α in saliva for both 2D- and 3D-SPEAu devices, which is even lower than that reported by Bellagambi et al. in 2017 [280,293].

Label-free TNF-α detection in saliva using an ion-sensitive field effect transistor (ISFET) with a silicon nitride transducer was performed by Ben Halima et al. in 2021 [283]. The bio-functionalization step in their study was initiated by creating an active OH group on the silicon nitride surface by placing the initial ISFET device in a UV/O_3_ cleaner for 30 min. The activated ISFET surface was then treated with (11-triethoxysilyl) undecanal (TESUD), and the functionalized ISFET was incubated with monoclonal anti-TNF-α antibodies (mAb-TNF-α) afterwards. The solution of ethanolamine was finally applied to the obtained ImmunoFET in order to avoid possible nonspecific bindings. The Nyquist plot for different concentrations of TNF-α (1–50 pg mL^−1^) was obtained (Figure 6E) with the real part of impedance on the *X*-axis and imaginary part of impedance on the *Y*-axis. It was then proved that the charge transfer resistance (R_ct_) increased with an increase in TNF-α concentration, which was explained by the formation of an insulating layer as more TNF-α molecules were bound to ImmunoFET. Ben Halima et al. obtained the limit of detection of 1 pg mL^−1^ for salivary TNF-α with good recovery of 94% ± 6%, and the method used in their study has also demonstrated high selectivity against interleukin-10 (IL-10), N-terminal prohormone of brain natriuretic peptide (NT-proBNP), and cortisol (Figure 6F), which are also considered heart failure biomarkers [283].

Since saliva is found to be a complex biological matrix, it contains various biomolecules and proteins that may interfere with and affect the performance of the electrochemical sensor [302]. Biosensing in complex biological media can also lead to an increase in background signal, reduced reproducibility, and low signal-to-noise ratio [303]. Different methods of sample pre-processing, such as dilution, filtration, precipitation, and centrifugation, can be useful to reduce fouling, but the sensitivity of the sensor can be decreased as well [303]. Alternatively, coating is one of the methods used to avoid matrix effect and improve sensitivity. Protective and functionalized coatings are used to improve the functionality of the surface layer of the biosensor by the incorporation of functional additives [304]. For example, polymer coatings on bioelectrodes are found to be useful for impedance reduction and signal quality improvement by an increase in signal-to-noise ratio and in charge transfer due to conducting polymers [304]. Electrodes modified with carbon nanotubes are also used in biodetection since they provide high surface area, good chemical stability, and excellent electrical conductivity [305]. Therefore, surface and sample modifications should be applied to improve the performance of the electrochemical biosensors in terms of sensitivity and selectivity and to reduce the matrix effect while analyzing complex biological media.

### 3.4. Surface-Enhanced Raman Spectroscopy

Surface-enhanced Raman spectroscopy (SERS) is a highly sensitive analytical technique that has several significant advantages over other methods. Apart from high sensitivity, SERS offers Raman fingerprint specificity, non-destructiveness, label-free detection, and the ability for multiplexed detection of biomarkers [306,307,308,309]. The surface enhancement is achieved by two types of enhancement, electromagnetic and chemical, the combination of which produces a signal enhanced by a factor of at least 10^6^ [310]. This is the main reason for the exceptional sensitivity of surface-enhanced Raman spectroscopy. Despite the many positive traits of SERS as an analytical technique, it possesses several disadvantages that need to be addressed. One of them is that there should be a profound contact between the SERS surface and the analyte to obtain a proper enhancement. In addition to that, other examples, such as susceptibility towards non-specific binding, poor reusability of substrates, degradation of substrates with time, and poor reproducibility of the SERS signal, can be mentioned [311].

**Figure 7:**
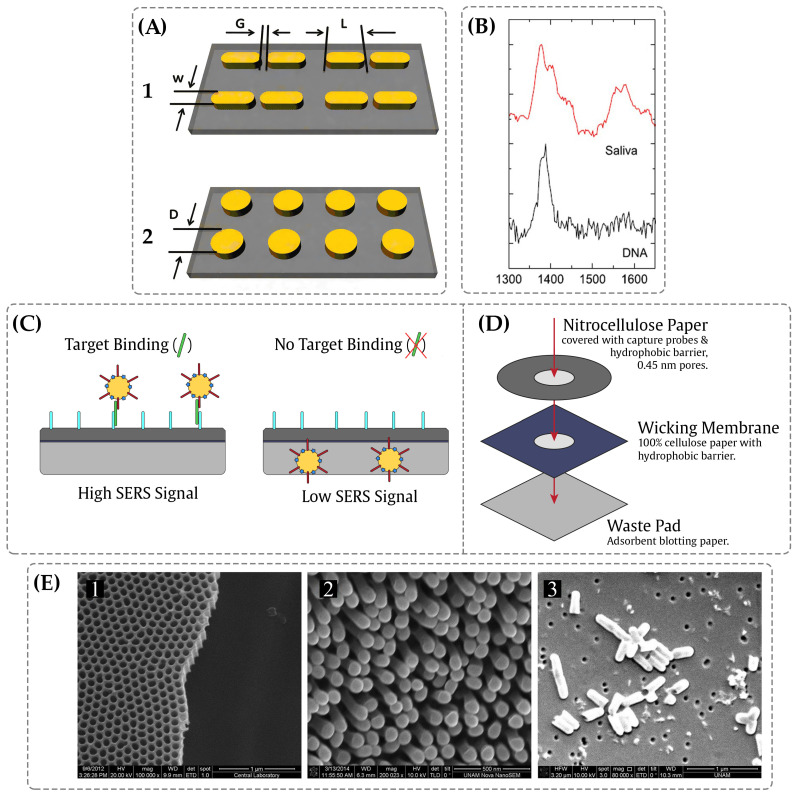
Schematic illustrations of two different plasmonic devices with gold nanorods (**A**) and gold nanocylinders (**B**). Adapted with permission from Cottat et al. [312]. Copyright © 2015 American Chemical Society. Schematic illustration of the VFA biosensor for S100P mRNA detection (**C**) and VFA composed of three paper layers (**D**). (**C**,**D**) are adapted with changes from Sungyub et al. [313], under © 2019 SPIE Creative Commons Attribution 4.0 Unported License. Scanning electron microscopy (SEM) micrographs of (1) the standard anodized alumina mold (AAM), (2) the resultant cylindrical nanopillar surface films, and (3) the sol–gel–synthesized silica nanotubes with straight tubular topography dictated by the cylindrical AAM pore templates (**E**). Adapted with permission from Altuntas et al. [314]. Copyright © 2018 Wiley-VCH Verlag GmbH & Co. KGaA.

Apart from being used in the detection of viruses and bacteria, the highly sensitive and versatile SERS technique has applications in the detection of different cancer biomarkers in saliva. One such application was demonstrated by Cottat et al. in 2015 [312]. They used SERS on gold nanocylinders and coupled nanorods for the detection of manganese superoxide dismutase (MnSOD). Increased levels of MnSOD correlate with cancerous cell lines, as well as severe chronic liver disease, which can be the cause of different types of carcinomas. Hence, a sensitive detection of MnSOD is highly important to monitor the stages of tumors and diagnose the disease early. Cottat et al. performed the detection of MnSOD in two body fluids, serum and saliva, with the LOD of 10 nM for both fluids. The detection of MnSOD in this study was not only highly sensitive but also showed high specificity, which is explained by the functionalization of the sensor surface with thiolated aptamers. This specificity is achieved due to the fact that aptamers are short DNA strands demonstrating higher affinity towards proteins in comparison with antibodies. MnSOD-specific aptamers were produced by automated solid-phase oligonucleotide synthesis and used a quartz microbalance (QCM) to evaluate the aptamers’ affinity to MnSOD. Regular SERS biosensors have a problem of nanoparticle agglomeration, which is why Cottat et al. used electron-beam lithography (EBL) to prepare gold nanostructures. The latter served as optical nanoantennas as they enhanced the Raman signal of MnSOD by several orders of magnitude. Figure 7A1 and Figure 7A2 show the schematic representations of two different plasmonic devices using gold nanorods and nanocylinders, respectively. Finally, Cottat et al. conducted the SERS measurements at 660 nm excitation wavelength for 140 nm diameter nanocylinders, and at 785 nm excitation wavelength for 200 nm diameter nanocylinders, as well as for the nanorod dimers. The comparison of the SERS spectra of MnSOD in saliva with that of the aptamer can be seen in Figure 7B [312].

Oral cancer is another quite prevalent and deadly type of cancer, with the main representative of this type of cancer being oral squamous cell carcinoma (OSCC), the sixth most common cancer in the world [315]. As was estimated by the World Health Organization, the number of new oral cancer patients increases by 529,000 each year, with more than 300,000 deaths [313]. OSCC shows a 5-year survival rate of 39–64% at stages III and IV, which is why it is very important to develop an analytical method with high sensitivity to detect this cancer at its early stages [313]. Salivary S100 calcium-binding protein P (S100) mRNA is proven to be a reliable biomarker for OSCC [316], which has the potential to decrease the number of biopsies and make the non-invasive early diagnosis possible. The levels of S100 mRNA are 2.5 times higher in the saliva of OSCC-diagnosed patients than in the saliva of healthy subjects [313]. Sungyub et al. in 2019 reported a detection of S100 mRNA in saliva based on a SERS assay of a free solution, as well as point-of-care (POC) detection using a vertical flow chip (VFC) [313]. They utilized gold nanoparticles (AuNPs) conjugated with oligonucleotides, and malachite green isothiocyanate served as the Raman reporter molecule. They combined the highly sensitive SERS technique with a rapid and rather simple paper fluidic assay for POC detection of S100 mRNA. Sungyub et al. applied PBS solution to the VFA biosensor (Figure 7C) in order to wash out DNA oligo-conjugated AuNPs that were not conjugated with the correct DNA oligo. Before proceeding to SERS measurements at 638 nm excitation laser, the VFA device, the schematic picture of which is shown in Figure 7D, was allowed to dry at room temperature [313]. As a result, Sungyub et al. achieved LOD values of 1.1 nM and 10 nM for the SERS assay in free solution and using VFC, respectively. Although the SERS assay with VFC demonstrated lower LOD than that of free solution, the entire analysis took less than 1 h (approximately 19 min for the VFC of saliva sample solution, the same amount of time for PBS washing solution, 10 min for drying of VFC, and 5 min for SERS measurements) [313].

There are several known biomarkers for Alzheimer’s disease, such as beta-amyloid 42 (Aβ-42), tau-441, and p-tau-181, with the former being the most common. Aβ-42 is a beta-amyloid peptide, which is a class of neurotoxic compounds that stimulate oxidative stress in the brain, eventually leading to neurodegeneration. Altuntas et al. performed the detection of Aβ-42 as the biomarker for Alzheimer’s disease using the SERS technique in 2018 [314]. Firstly, they used a two-step anodization method for the preparation of standard anodized aluminum molds (AAMs), in which high-purity aluminum foils underwent mechanical cleaning and electropolishing, followed by anodization in the solution of oxalic acid. Consequently, the oxide film was removed using chromic acid solution, and a second anodization was done by Altuntas et al. in order to obtain AAMs fabricated on aluminum foil with uniform pore distribution. Secondly, multibranched AAMs were obtained through another anodization using sulfuric acid. Altuntas et al. then used standard (Figure 7E) and multibranched AAMs to obtain cylindrical nanopillar surfaces (CNS), an SEM image of which is shown in Figure 7E, and multibranched nanopillar surfaces (MNS). They additionally modified the nanostructured surfaces with thioflavin-T (ThT), which is a fluorescent molecule capable of significant enhancement of the SERS signal. Due to the ability of ThT to covalently bind to the metal surfaces, it can produce a high-intensity characteristic Raman signal. Altuntas et al. managed to perform the detection of Aβ-42 in artificial saliva with the LOD value of 0.5 pg mL^−1^ and a rather good linear dynamic range from 0.5 pg mL^−1^ to 100 ng mL^−1^ [314].

A more recent detection of cancer biomarkers using the SERS technique was performed by Sunil et al. in 2023 for the pre-diagnosis of lung cancer [317]. Lung cancer is considered to be one of the deadliest cancer types, mostly due to its detection at advanced stages. The 5-year survival rate for lung cancer at advanced stages is 18%. Sunil et al. managed to detect a salivary imidazole compound, potential lung cancer biomarkers, with a detection limit of 10^−12^ M and a good enhancement factor of 4.2 × 10^7^. Bimetallic Nickel@Silver core–shell nanoparticles (Ni@Ag/CNFs) functionalized on carbon nanofibers were used as a highly sensitive and label-free SERS-based platform for salivary detection. The authors were able to enhance SERS spectra of imidazole compounds in the clinically relevant range (0.2–0.6 mM) in a real saliva sample, showing its potential in clinical diagnosis [317].

### 3.5. Colorimetric Detection

Colorimetric detection is one of the analytical methods that gives direct information about the analyte. The colorimetric method is based on a visual change in the color of the analyzed solution and test strip. This can happen because of a chemical reaction with reagents such as redox and pH indicators and nanoparticles. Some reactions result in a change in pH or redox properties instead of a direct color change [318].

This approach allows for visual results without the need for complex equipment, which is critical for rapid diagnostics. Colorimetric sensors have gained widespread popularity due to the method being quick, inexpensive, and portable [318,319]. The examples of colorimetric method application in the detection of analytes in saliva are presented in Table 6. Calculated geometric average and median of LOD for the reported in seven papers in the last decade were 1.3 × 10^−5^ M and 3.61 × 10^−5^ M, respectively.

Ming et al. presented a colorimetric biosensor based on a G-quadruplex DNAzyme with a low background signal for detecting the oral cancer overexpressed 1 gene (ORAOV1 gene), a tumor marker for oral squamous cell carcinoma (OSCC) in saliva. This approach relies on competitive binding, which releases a G-rich sequence and enables the formation of a catalytic complex. The visual detection threshold was 1 × 10^−8^ M, making the sensor suitable for non-invasive early detection of cancer [332]. This is the lowest LOD value in Table 6.

There are groups of publications that focus on implanted colorimetric methods [336,337]. For example, Mirzaei et al. developed an origami paper sensor with sericin-coated silver nanoparticles (NPs) for colorimetric detection of hydrogen peroxide and glucose. The NPs catalyze 3,3′,5,5′-tetramethylbenzidine (TMB) oxidation by H_2_O_2,_ producing a blue color visible to the naked eye. For glucose, glucose oxidase generates H_2_O_2_. Detection limits are 0.15 mg/dL for peroxide and 0.37 mg dL^−1^ for glucose, with analysis times of 4.5 and 7 min. The sensor was tested on spiked saliva (one donor) and serum, showing good correlation with clinical methods. It is simple, low-cost, stable for 42 days, and suitable for point-of-care diabetes monitoring [338]. However, we do not consider this group of applications since we focus only on the analysis of non-invasive saliva, which is spit by patients into the tube, and it is beyond the scope of this review.

Results showed that colorimetric detection have significantly lower sensitivity compared to EIS, optical immunoassays, voltametric methods and MS: both the geometric average and median value of the LOD for colorimetry (10^−5^ M) are several orders of magnitude higher than for EIS (10^−15^ M), optical (10^−13^ M), voltametric (10^−13^ M) and mass spectrometry (10^−9^ M), which indicates a lower sensitivity of colorimetric technique despite their simplicity and cost. Despite this, the method is in the stage of rapid development [339].

Hence, colorimetry-based sensors offer a relatively simple, cost-effective, and rapid alternative to some traditional detection methods, which are usually based on heavy instrumentation and highly trained staff, such as mass spectroscopy. Colorimetry has the great advantage of low-resource setting operation and on-site monitoring. A defining attribute of colorimetry is its readout mechanism, which enables point-of-care diagnostics, where analytes can be identified immediately with the naked eye or widely spread electronic accessories, such as smartphones or flatbed scanners. Furthermore, upon integration with platforms like the mentioned microfluidic papers, saliva detection with colorimetric methods minimizes the consumption of the reagents and enables autonomous, non-invasive detection of target molecules in very low sample volumes, down to dozens of µL, while still achieving acceptable analytical precision. Taken together, the presented studies demonstrate that colorimetry has evolved beyond simple color-change-based qualitative tests into a quantitative technique, sometimes allowing multiplexable, field-deployable sensing. While the calculated average value of the detection limit for this technique is very modest compared to other considered analytical methods, the colorimetry may at least partially compensate for it with the above-mentioned advantages. However, there is still a significant room for improvement in this method, particularly in terms of sensitivity.

### 3.6. Cortisol Detection

Cortisol, also known as a “stress hormone”, is a steroid hormone produced by the adrenal glands in response to stress. It plays an important role in regulating metabolism, immune function, blood pressure, and other bodily processes. The level of cortisol in bodily fluids such as saliva provides useful information about the development of different diseases. For example, excess cortisol levels in the 100–500 nM range may indicate a high chance of serious conditions such as Cushing’s or Addison’s disease [340,341].

Owing to its importance as a non-invasive biomarker of various health conditions and diseases, in this section of the review, we will try to compare several methods (e.g., mass spectroscopy, immunoassays, and electrochemical techniques) using cortisol as a model analyte. Table 7 summarizes all analytical studies that were used for the comparison. We compared different techniques based on their limit of detection (LOD). The limit of detection is defined as the lowest concentration of analyte at which its signal is 3 times greater than the background noise [342]. The low limit of detection (LOD) of cortisol below its physiological levels may be useful for the evaluation of different analytical techniques. The physiological levels of cortisol in healthy individuals vary by time of day. For example, at 8 AM, the physiological cortisol concentration in healthy individuals lies in the range 3.5–27 nmol L^−1^, while it decreases below 6 nmol L^−1^ in the evening at 10 PM [343].

In total, we analyzed 28 analytical papers with reported LOD, including eight papers on immunoassay-based detection, 13 electrochemical (five electrical impedance spectroscopy, five voltammetry, three chemiresistance and electrical chips, reported as “other” category in Table 7), and eight mass-spectroscopy-centered studies. Based on average LOD, electrochemical methods have up to two orders of magnitude lower average detection (LOD) compared to mass spectroscopy and immunoassays (MS and IA: 10^−11^–10^−10^ M vs. electrochemical: 10^−11^ to 10^−12^ M). The similar trend is observed in median LOD values: electrochemical methods have approximately 10 times lower median LOD: MS 8 × 10^−11^ M, IA 6 × 10^−11^ M vs. ~10^−11^–10^−12^ M. Regarding the electrochemical part, the classification was based on the detection method reported in original studies. The “other” category includes studies other than electrical impedance spectroscopy (EIS) and voltammetry. All electrochemical methods have a more or less similar average LOD of cortisol in the picomolar range.

In terms of reproducibility, electrochemical methods can potentially be better than mass spectroscopy. For example, from our calculations, the average relative standard deviation (RSD) value of electrochemical techniques is roughly four times smaller than that of mass spectroscopy: 9% and 2.5%, respectively [342,348,350,359,361]. It means that the precision of measurements using electrochemical methods can be better compared to mass spectroscopy. Besides the comparatively smaller size of most electrochemical devices, their portability, rapid and cost-effective detection procedure, they might be a valid alternative to more complex, expensive, and time-consuming methods of analysis, such as mass spectroscopy, which are highly dependent on laboratory instrumentation and highly trained personnel. Moreover, electrochemical devices can be operated in a real-time fashion to provide a true representation of cortisol levels in saliva in response to environmental changes that trigger cortisol generation or suppression. For example, Khan et al. fabricated a paper-based electrochemical biosensor chip for the real-time detection of salivary cortisol levels [362]. In their approach, they integrated the sensor chip with a hand-held mini electronics printed circuit board (PCB) and directly incubated the saliva sample with a sensor chip. The dynamic sensing of cortisol was possible due to the square-ring arrangement of Au electrodes, which produced a high electrical field within neighboring electrodes. A DC voltage of 5 V was applied to the microcontroller embedded in the PCB, and then signals were transferred to the Bluetooth device. The signals were monitored through MATLAB in real time, and the resistance readings were recorded. Unlike EIS and voltammetry methods that are generally used to detect bioanalytes on the sensor surface, in their approach, Khan et al. did not use any electrolyte, since the saliva was directly added to the surface of the chip, and the change in resistance was recorded once cortisol was added to the antibody-immobilized biosensor [362]. These types of electrical biosensors can potentially be assembled in hand-held devices, and the readout data can be shared wirelessly in real-time with clinicians through tablets, smartphones, or laptops.

Most electrochemical-based techniques covered in this section basically utilize a similar working principle as most immunoassays. Electrochemical immunosensing is based on the idea of measuring changes in the electrical properties of a conductive material upon absorption of the analyte on an antibody-immobilized surface. For example, Liu et al. fabricated an immunosensor based on a miniaturized differential pulse voltammetry (DPV) system in a smartphone to monitor non-invasive cortisol levels in saliva. They used AuNPs/MoS_2_/AuNPs as a transducer in their system, and cortisol antibody (CORT-Ab) was bound to the transducer through a covalent bond. Non-specific binding issues were avoided by passivating the surface of the transducer with ethanolamine. The limit of detection (LOD) was as low as 10^−10^ M, by two orders of magnitude lower than immunoassay-based detection [358].

There are several challenges that need to be addressed for electrochemical methods to be as widely used in daily clinical investigations as traditional techniques such as ELISA or radioimmunoassay. The matrix effect remains a common issue for all types of electrochemical immunoassays since saliva is a complex biological fluid rich in interfering analytes (proteins, enzymes, hormones, antimicrobial agents). This effect was well illustrated by Tilli et al., who fabricated a label-free immunosensor based on a carbon-nanotube chemiresistive transducer functionalized with cortisol analog and anti-cortisol antibody [361]. When a 10-fold diluted artificial saliva sample was used, the resistance showed no net change, suggesting that something bound to the sensor surface nullified the expected decrease. They revealed that non-specific binding of mucin, a group of glycoproteins abundant in saliva, on the gold electrodes likely caused displacement of anti-cortisol. This was addressed by treating the Au electrode with mercaptohexanol during fabrication, achieving a LOD of 1 pg mL^−1^, well below the therapeutic cortisol level of 1 ng mL^−1^, with results correlating well with ELISA (R^2^ = 0.995) [361]. Beyond such targeted blocking strategies, several broader approaches have been developed to mitigate biofouling and the matrix effect in salivary electrochemical detection. The most straightforward is sample dilution, which reduces protein load and viscosity [365]. Another widely used approach involves modifying electrodes with hydrophilic anti-fouling coatings such as polyethylene glycol, zwitterionic polymers, or other types of synthetic polymers such as poly(hydroxyfunctional acrylates), poly(2-oxazoline)s, poly(vinylpyrrolidone), poly(glycerol) [366,367]. Nanoporous electrode architectures offer an alternative by physically excluding large fouling biomolecules while allowing smaller target analytes to diffuse through for detection [368]. As a combined example of these principles, Nong et al. developed a disposable anti-biofouling sensor by modifying laser-induced graphene (LIG) electrodes, which themselves possess a nanoporous structure, with a composite film of BSA and Tween-20 (BSAT) via simple drop casting [365]. The resulting ultrathin hydrophilic coating resisted protein adsorption while still allowing uric acid to access the electrode surface. Combined with 5-fold sample dilution and the standard addition method, the sensor achieved reliable UA detection in human saliva with recoveries of 96.4% to 105.0%.

In addition to the matrix effect, most electrochemical methods of cortisol detection rely on antibody-target analyte interaction. The storage and transportation of not only antibodies, but also oral fluids require special storage and shipping conditions. For example, under ambient temperatures below 22 °C, the samples with oral fluid should be transported to the lab within 24 h (WHO Expanded Programme on Immunization, 2006). These criteria might not always be met by all countries and laboratories with limited access to reagents and transportation issues. As a result, it might affect the results of electrochemical detection from lab to lab. Finally, in addition to storage and transportation issues, non-specific binding and aggregation of nanoparticles remain a common issue for immunosensors [369,370]. There are several possible ways to solve these issues, such as the use of blocking solutions (e.g., bovine serum albumin (BSA)), optimizing conditions (incubation time, the choice of buffer), and the choice of nanoparticle synthesis strategies (e.g., PEGylation to prevent nanoparticle agglomeration) [371,372,373,374].

Overall, due to their compact size, rapid and sensitive detection, portability, and low cost, electrochemical methods of detection are worth substantial testing for daily clinical investigations and for the development of point-of-care (POC) devices.

## 4. Clinical Applications

Saliva has the potential to be a useful diagnostic tool in a variety of clinical settings due to its non-invasive nature and ease of collection. Owing to its complex composition, saliva can be used in disease diagnosis (cancer, periodontitis, tuberculosis) [375,376], genetic and hormone testing [377], in the detection of infectious agents (SARS-CoV-19) [163], gland disorders (Sjögren’s syndrome), and salivary gland tumors [378]. In this section, we focus on different clinical detection methods that include mass spectroscopy, enzyme-linked immunosorbent assay, PCR, Raman spectroscopy, and other methods such as Western blotting, lectin blotting analysis, etc. The major performance parameters include sensitivity and specificity. The sensitivity refers to the ability of the method to detect the analyte when it is present in a sample (rate of true positives: 100% minus the false-negative rate). The specificity is defined as the ability to detect a negative result when the analyte is absent in a sample (rate of true negatives: 100% minus the false-positive rate) [136]. Overall, for the evaluation of clinical performance, we analyzed 65 clinical articles: 10 on mass spectroscopy, 22 on PCR, 14 on ELISA and other immunoassays, eight on SERS and Raman spectroscopy, seven on immunoassay-based methods, and 11 research articles on other methods (e.g., Western blotting, electrochemical methods).

### 4.1. Polymerase Chain Reaction (PCR)

PCR has several advantages in clinical diagnostics. It is a simple, sensitive method with the potential to produce millions to billions of copies of a desired DNA fragment. Moreover, PCR can be useful for qualitative as well as quantitative analysis. For example, Min et al. used the PCR method not only for screening of deregulated miRNAs in saliva, but also for quantification for the early diagnosis of Hand, Foot and Mouth disease (HFMD) in pediatric patients [75]. The quantification ability of PCR enables researchers to study changes in the expression levels of genes in tumors and other disease states. The major limitations of the PCR technique are associated with the reaction conditions, product yield, and specificity. For example, the DNA polymerase enzyme, which is required for PCR, is susceptible to errors and can cause mutations in the DNA fragment generated. Primers can nonspecifically bind to similar sequences on the template DNA [74]. The major performance parameters for PCR-based clinical studies are summarized in Table 8 below.

Apparently, in clinical diagnostics, PCR has a wide range of applications from cancer diagnostics to gland disorders detection. For example, Sehovic et al. demonstrated the application of quantitative real-time PCR (qRT-PCR) in the early detection of salivary miRNAs for autism spectrum disorder (ASD) identification [23]. Autism spectrum disorder is a developmental disability caused by changes in the brain. Individuals with ASD have problems with communication and interaction as well as unusual social behavior. The estimated prevalence of ASD in the U.S. has increased by 1.2% from 2008 to 2018 [393]. The worldwide prevalence of ASD is estimated to be 1–2% of the population, but this value varies in each country depending on the availability and level of diagnostic and screening tools [23]. The early diagnosis of ASD is imperative from a long-term perspective and may improve the quality of life of children with ASD [394]. In their work, Sehovic et al. analyzed 80 saliva samples (55 positive for ASD, 25 control samples) and determined that five salivary miRNAs are potential biomarkers of ASD. The logistic regression prediction model had a 90.3% sensitivity (95% CI: 63.7–96.5%), 90% specificity (95% CI: 77.9–99.2%), and an area under the ROC curve of 0.952. This study has shown that a relatively cheap method, such as qRT-PCR, can be used for ASD detection and may identify ASD along with other standard screening tests.

In addition to neurodevelopmental disorders, there are numerous studies on the application of PCR in cancer detection. The clinical articles about PCR detection are summarized in Table 8. From Table 8, Oh et al. evaluated mRNA biomarkers for the early diagnosis of oral squamous cell carcinoma using saliva samples of 67 patients (33 OSCC, 34 non-tumor). The diagnostic sensitivity at 92%, specificity at 86%, and AUC equal to 0.91 were achieved in patients under 60 years of age. For comparison, for the detection of OSCC, the sensitivity value by the RT-PCR method in the work of Oh et al. [386] was significantly better (sensitivity: 92% vs. 70%, AUC 0.91 vs. 0.84) than that of the ELISA method [395], which is considered a gold standard of immunoassays and is used as widely as PCR in the detection of different disease biomarkers. In terms of cost, though RT-PCR testing is more expensive than ELISA testing (e.g., 13.2–15.4 $ vs. 6.59–10.31 $ for a COVID test, respectively) [396,397]. It is more sensitive and more automated, thus it requires less labor, which makes it suitable for point-of-care (POC) detection.

### 4.2. Mass Spectroscopy

Mass spectroscopy is a powerful analytical technique that is widely used in clinical detection to identify and quantify biomolecules such as proteins, peptides, metabolites, and drugs. In clinical applications, mass spectroscopy is used to diagnose diseases, monitor treatment efficacy, and evaluate disease progression [398]. The main advantage of mass spectroscopy over other analytical techniques is its ability to provide highly sensitive and specific measurements of analytes in complex biological matrices. This is achieved by ionizing the biomolecules of interest and separating them based on their mass-to-charge ratios (*m*/*z*) in a mass spectrometer. The resulting mass spectrum provides a unique fingerprint of the biomolecules present in the sample [399]. Moreover, mass spectroscopy can easily be coupled with other analytical techniques, such as liquid chromatography [27] and gas chromatography [400] to provide more complex information about disease progression and analysis.

In clinical detection, mass spectroscopy is used in a variety of applications, including proteomics, metabolomics, microbial infection, and other types of biomarkers [24,34,401]. Metabolomics is a large area of study of small molecules within cells, biofluids, tissues, and organisms. The study of these small molecules and products of metabolism is collectively known as “metabolome”. Transcriptomics is the study of RNA and mRNA expression [402].

Metabolomics has great potential for early detection of disease biomarkers. In their work, Liang et al. applied ultra-performance liquid chromatography (FUPLC) mass spectrometry (MS) method to find metabolomics changes in the saliva of patients with Alzheimer’s disease (AD) [24]. In total, they analyzed 474 saliva samples (256 with AD, 218 healthy controls). Three metabolites (AUC > 0.8), including sphinganine-1-phosphate, ornithine, and phenyllactic acid, were selected as candidate biomarkers, sphinganine-1-phosphate being a major contributor to the predictive model of AD. The proposed method had a clinical sensitivity of 99.4%, specificity of 98.2%, and an AUC equal to 0.998. The integrative approach of combining MS with liquid FUPLC demonstrated satisfactory performance in distinguishing AD patients from healthy individuals. Table 9 below demonstrates the major FOMs (Figures of merit), which have been reported in clinical studies based on an application of mass spectrometry.

In another study by Deutsch et al., label-free quantitative mass spectrometry analysis (qMS) was used for the early detection of pancreatic cancer (PC) biomarkers based on the analysis of the proteome of 15 PC patients and 16 healthy controls [401]. qMS analysis yielded 90% sensitivity, 90% specificity, and an area under the ROC curve (AUC) equal to 0.91. For comparison, a similar proteomic analysis of pancreatic cancer using the qPCR method [31] yielded relatively worse sensitivity (78.2% vs. 90%) and AUC (0.88 vs. 0.91) than MS analysis.

Along with numerous advantages that MS has in clinical analysis, it also has several limitations. The major limitations of MS-based detection include cost, lengthy sample preparation, matrix effect, and separation issues [406]. The mass spectrometers are expensive to purchase, operate, and maintain, which causes several challenges to their application in small clinical laboratories and routine clinical analysis. For example, the metal analyses by GC/MS or LC/MS cost between 100 and 200 $ per sample (University of Florida Health, 2023), which is 3–6 times more expensive than ELISA (~34 $ per sample) testing [407]. The sample preparation step for MS analysis is time-consuming and requires a certain skill set. The lengthy protocol with all steps can take up to 4 h. Sustainability is another issue in MS-analysis since the sample preparation includes the use of harsh organic solvents [406].

Recently, a more sustainable and greener technology named QuEChERS (quick, easy, cheap, effective, rugged, and safe) has been developed that uses cleaner extracts from complex media such as tissues or blood samples in less than 30–45 min. The QuEChERS technology is fast, and extracts can be easily analyzed by LC/MS. The following technology combines two basic methods (e.g., solid-phase extraction and liquid/liquid extraction) into one dispersive solid-phase extraction procedure (d-SPE), where the concentrations of required salts and solvents (e.g., acetonitrile solvent, inorganic salts MgSO_4_, NaCl) are optimized to improve analyte recovery [408]. The RapidFire system is another alternative to QuEChERS technology. It does not require additional centrifugation steps, can be automated and interfaced with a mass spectrometer via electrospray ionization (ESI), and finally, it can be used to decrease false positive rates in screenings. Moreover, the results from biological matrices can be obtained in less than 30 s [406].

### 4.3. ELISA, Immunoassays

Immunoassays are commonly used in clinical analysis and disease diagnostics. Since its discovery in the 1970s, the enzyme-linked immunosorbent assay (ELISA) has been considered a gold standard of immunoassays [44]. The following section focuses on the discussion of ELISA and other types of immunoassay-based detection methods and their application in clinical diagnostics. Table 10 summarizes all clinical studies for ELISA/Immunoassay-based detection.

In their work, Omran et al. used ELISA-based detection of C-reactive protein (CRP) as a biomarker of neonatal pneumonia that accounts for 10% of global child mortality worldwide [46]. 70 neonates were enrolled in the study (35 neonatal pneumonia cases, 35 healthy controls). Salivary CRP concentrations were measured by ELISA. At the cut-off value of 3.8 ng L^−1^, salivary CRP demonstrated 91.4% sensitivity and 80.9% specificity.

Compared to ELISA, electrochemical assays demonstrate relatively better sensitivity and specificity values in the detection of various disease biomarkers. For example, Cao et al. investigated the presence of exosomes in salivary samples of patients with Parkinson’s disease (PD) using an electrochemiluminescence assay (ECL) [25]. In the analysis of saliva samples from 74 PD and 60 health controls, the sensitivity reached 92%, specificity 86%, and AUC 0.941. In another study that utilizes an electrochemical immunoassay, the authors investigated the early detection of celiac disease using antibodies (anti-tTG IgA) as a potential biomarker [35]. The sensitivity 95%, the specificity 96%, and the AUC was equal to 1.00. Celiac disease is a gluten-induced autoimmune enteropathy that is usually found in genetically susceptible individuals. It is one of the common immune diseases in Europe and North America (1–1.2%) [418]. The system in the work of Adornetto et al. used magnetic beads (MBs) covered with anti-transglutaminase (tTG), which reacted with anti-tTG IgA antibodies in positive saliva samples for celiac disease [35]. The anti-human IgA, conjugated with alkaline phosphate (AP) enzyme, was used as the label, and a strip of eight magnetized screen-printed electrodes was used as the electrochemical transducer. To improve the sensitivity and reproducibility, they performed electrochemical pretreatment by applying anodic potential of 1.7 V for 180 s in 0.05 M phosphate buffer (pH 7.4). To reduce nonspecific binding of glycoproteins (present in saliva) on the magnetic bead surface, the authors optimized the type of blocking agent and buffer to dilute saliva and anti-human IgA. The best results were obtained from gelatin as a blocking agent for magnetic beads (MBs), and M-PBS with RAD buffer.

Compared to mass spectrometry, which was discussed in the previous section, the immunoassay-based approach is relatively more cost-effective (e.g., ELISA is 3–4 times less expensive than MS in metal analyses of water samples), which makes it accessible to laboratories with limited resources. Moreover, immunoassays require less amount of sample and preparation time compared to MS, and there is generally a uniform protocol for automated high-throughput [406]. Unlike MS, immunoassay preparation does not require highly trained personnel and is highly specific due to antigen–antibody interactions, which makes it suitable for the analysis of complex biological fluids such as saliva [419]. However, immunoassay-based methods also have their limitations, including non-specific binding issues, aggregation of nanoparticles, and antibody degradation during storage and transportation. Non-specific binding complicates the detection of target molecules by reducing the sensitivity and specificity of immunoassays. The possible reasons for non-specific binding in immunoassays might be a slow diffusion rate of nanoparticles and a high level of non-specific interactions with a surface, producing, in this way, less desired antibody–antigen interactions [420,421]. Several studies suggested an optimization of blocking solutions (e.g., bovine serum albumin (BSA)), conditions (incubation time, the choice of buffer), and the choice of nanoparticle synthesis strategies (e.g., PEGylation to prevent nanoparticle agglomeration) to reduce non-specific adsorption [371,372,373,374].

### 4.4. Other Methods (SERS, Raman Spectroscopy, Electrochemistry)

In this section, we are going to focus on the clinical studies that are limited in number but are essential in our opinion for the comprehensive understanding of various saliva-based detection methods in clinical diagnostics. Our discussion starts with the surface-enhanced Raman spectroscopy.

Raman scattering is a form of inelastic scattering, where the frequency of the scattered photon differs from the frequency of the incident photon. By measuring the difference in frequency and energy, information about the molecule can be obtained since these differences are molecule-specific [422]. Raman spectroscopy is a non-destructive chemical analysis technique, which is useful for molecular fingerprinting. However, it has low sensitivity due to very weak Raman scattering. The enhancement in Raman signal intensity can be achieved using surface-enhanced Raman scattering (SERS) [423,424,425]. The phenomenon of surface-enhanced Raman scattering was discovered in 1974 by Fleischmann, Hendra, and McQuillan from the University of Southampton, UK [426]. They observed surprisingly strong and potential-dependent Raman signals of pyridine adsorbed on electrochemically roughened silver electrodes. Fleischmann et al. explained the enhancement in Raman intensity due to an increase in the surface area of the Ag electrode; however, this was not a major reason for the Raman enhancement. Later, in 1977, Jeanmaire and Van Duyne from Northwestern University (USA) proposed the theory for Raman signal enhancement [426]. Based on this theory, electromagnetic enhancement (EM) and chemical enhancement (CE) are two major mechanisms contributing to the SERS effect. EM mechanism contributes dominantly to the SERS effect, owing to 4 to 11 orders of magnitude enhancement, while CE is only 10–100 times [427]. Up to now, the physics of EM is well understood and widely applied to the experimental design of SERS, while the CE mechanism is yet to be further explored [427]. Table 11 below summarizes the performance parameters reported for quantitative clinical studies based on Raman, SERS, and some other analytical techniques.

Despite the limited number of clinical articles on saliva-based SERS detection, among the studies that we reviewed, SERS demonstrated promising results in terms of sensitivity and specificity. For example, Hernández-Arteaga et al. achieved 94% sensitivity and 98% specificity for the detection of breast cancer using sialic acid (SA) as a potential cancer biomarker [429]. The cutoff SA concentration was >7 mg dL^−1^ for positive test results. The key idea behind their approach was to use silver nitrate with trisodium citrate to reduce silver colloids, which are widely used as SERS substrates due to the ease of preparation, long shelf life, and high Raman enhancement [445]. As a result, the surface of silver nanoparticles was covered by a layer of negative citrate ions. By controlling the colloid surface layer, the prepared Cit-AgNPs were found to be effective at the detection of both cationic and anionic species, such as sialic acid [429].

Despite excellent optical capacity, SERS has not yet been used for real practical applications. Several factors, such as non-specific binding, reproducibility, and high cost, hinder the promotion of SERS for real-life practical applications [446,447]. There are several possible ways to address these issues. For example, non-specific binding can be resolved by introducing a rotating substrate [448], nanoparticle coatings [429], decreasing assay time [431], etc. The cost of SERS-based measurements can potentially be solved by using portable Raman spectrometers that have been widely used for in situ SERS analysis [449]. Moreover, the introduction of relatively cheaper non-noble metal-based substrates, such as aluminum or silicon, may reduce the cost of substrate preparation [450,451]. In the future, prospective efforts have to be focused on the development of SERS-based platforms with a small size, reduced cost, low sample consumption, easy operation, and intuitive readout.

In most cases, a single biomarker alone is not an effective tool for accurate diagnosis of the disease, since the biological system is quite complex. Moreover, the detection based on a single biomarker has a high possibility of false positive and negative results. The usual accuracy of salivary biomarkers is in the range from 0.65 to 0.85, which is far from real clinical terms [452]. The combination of multiple biomarkers not only improves overall accuracy but is also essential for accurate diagnosis. Kozak et al. demonstrated that the combination of 3–5 protein biomarkers can improve the detection of early-stage ovarian cancer by 8% compared to the CA125 biomarker alone [453]. Therefore, it is important to develop technology that addresses multiplexed detection. Along with SERS, electrochemical (EC) sensors can be easily expanded into multiplex detection platforms that are simple to operate, sensitive, and specific [454]. Several studies reported that EC sensors have demonstrated good sensitivity and specificity for nucleic acid and protein biomarker detection in saliva [455,456]. For example, Wei et al. studied the application of an electrochemical sensor for the detection of oral cancer based on the simultaneous detection of interleukin (IL)-8 mRNA and IL-8 protein [290]. The receiver operating characteristic (ROC) analysis yielded 83% sensitivity and 87% specificity for IL-8 mRNA. For IL-8 protein, both sensitivity and specificity were 87%. The AUC value was around 0.9 for both analytes. These results are close to the results measured by ELISA (AUC 0.87) and PCR (AUC 0.91) of the same group of proteins, which means that the EC sensor can accurately measure salivary biomarkers similar to conventional ELISA and PCR methods.

There are also applications of machine learning algorithms in clinical studies for these other optical methods. For instance, the study by Guevara-Vega et al. focused on the detection of Chikungunya Virus in mice by means of a portable ATR FT-IR platform, in contrast to the gold-standard invasive and costly molecular biology procedure [444]. With the help of Support Vector Machine (SVM) and linear discriminant analysis (LDA) algorithms, they managed to differentiate between healthy and diseased samples with an accuracy of 86% by classifying complex spectral overlaps with these ML methods, which would be impossible in manual analysis. The researchers are planning to increase the sample size as well as validate the findings in human saliva samples to confirm the clinical relevance of the suggested virus detection method. In another study conducted by the research group of Dr. Sajan D. George, the researchers introduced the plasmonic SERS droplet assay platform combined with a custom-built portable Raman system for saliva-based oral cancer detection [433]. Their platform significantly reduces the volume of saliva required for an assay (initial aliquot only 20 µL). These 20 µL are further split into sub-µL drops, allowing the multiplex analysis via nu-SVC-based ML-classification model, resulting in effective differentiation between healthy, premalignant, and malignant saliva samples, with the overall accuracy exceeding 85%. The droplet splitting technique on a suggested wettability-engineered substrate allows for performing multiple trials of SERS measurement from the same saliva sample, improving both efficiency and reproducibility. The authors believe that the validation against a higher sample size and scaling of the platform fabrication are further required to transition the developed platform to clinical settings.

## 5. Challenges and Outlook

Regarding the clinical part, one of the challenges in any comparative review, including this one, is the variability of sample parameters such as average sensitivity and specificity values. In the calculation of these two clinical performance parameters, we did not take into account the size of the sample. The heterogeneity in the sample size may decrease the reliability of conclusions about the performance characteristics of different methods discussed in this review. Moreover, among the studies that we reviewed, different multivariate statistical algorithms such as principal component analysis (PCA), linear discriminant analysis (LDA), Kruskal–Wallis analysis, and Wilcoxon rank-sum test were used to distinguish healthy controls from positive cases [22,28,375]. The performance of each of these statistical methods may vary, which eventually may cause differences in sensitivity and specificity values. For example, in his work, Li et al. compared two types of multivariate statistical algorithms: genetic algorithm combined with linear discriminant analysis (GA-LDA) and principal component analysis (PCA) for the differentiation of bladder cancer patients from healthy individuals [457]. It turned out that the GA-LDA produced better sensitivity (90.9% vs. 74.6%) and specificity (100% vs. 97.2%) than PCA. A similar observation was also made in a group of Li et al. [458].

It is worth mentioning that the choice of biomarker also plays an important role in the comparison of different methods, since different biomarkers have different diagnostic accuracies and sensitivities. For example, by the same qPCR method, gene-based detection of pancreatic cancer yields better sensitivity (96% vs. 71%) than the mRNA-based detection [33,375]. For the uniformity of our results, we suggest considering the comparison not only by method, but also by the type of biomarker in the future.

Lastly, due to the restricted number of clinical articles with reported accuracy values, we did not take them into account in our comparisons. Though accuracy is another important analytical parameter that measures the rate of correct results and is reported in % [136]. Taking into account all of these factors, we should consider in our future work the uniformity of comparative analysis and possibly implement another approach for the evaluation of analytical and clinical performance of different methods.

To evaluate the analytical performance of different techniques, we selected cortisol as a model analyte, a clinically proven and “gold standard” biomarker of chronic stress and anxiety disorders [459]. In total, we analyzed 29 analytical papers, focused mainly on three techniques: mass spectroscopy, immunoassays, and electrochemical methods (e.g., electrical impedance spectroscopy, voltammetry, and chemiresistors). We compared the analytical performance of each method based on its average and median limit of detection (LOD). Out of three electrochemical-based techniques, the voltammetry and chemoresistance techniques demonstrated better average and median LOD, with 1–2 orders of magnitude lower results compared to mass spectroscopy and immunoassays (e.g., lateral flow, microfluidic, chemiluminescence, aptamer-based), with specific numbers available in Table 7. Based on average LOD values, the sensitivity of electrochemical techniques allows the detection of salivary cortisol in picomolar ranges, by three orders of magnitude lower than the physiological levels of cortisol in healthy individuals. In real-life clinical applications, it means that electrochemical methods can enhance early detection of various diseases. As it was mentioned previously, a 10-fold increase in LOD is likely to increase the rate of false negative results by 13%, which equals missing one patient in 8 diseased patients [54]. Therefore, the lower the LOD, the better the diagnostic accuracy and subsequent treatment. In terms of reproducibility, electrochemical methods also demonstrated better results than mass spectroscopy. The average of three RSD values for each method produced a 4 times lower average RSD for the electrochemical method than for mass spectroscopy, 9% and 2.5%, respectively. The multiplex detection, high sensitivity, and specificity of electrochemical methods (e.g., voltammetry, electrochemical impedance spectroscopy), low-cost, portability, and simplicity in operation make them attractive as future point-of-care (POC) devices.

Regarding the clinical part, in total, we analyzed 67 clinical articles to evaluate the clinical performance of different methods (e.g., mass spectroscopy, PCR, ELISA, Immunoassays, SERS-based assays, etc.). Clinical performance was characterized by three important parameters: sensitivity, specificity, and the area under the ROC curve (AUC) value. The sensitivity (%) refers to the ability of the method to detect the analyte when it is present in a sample. The specificity (%) is defined as the ability to detect a negative result when the analyte is absent in a sample [136]. The area under the ROC curve (AUC) defines the overall accuracy of the test in classifying individuals with and without disease or illness. The greater the AUC, the better the discriminatory power is. The AUC of 0.5 indicates no discrimination, 0.7–0.8 is considered acceptable, and above 0.9 is considered outstanding [460]. Comparison of all three parameters is important to evaluate the clinical performance of various methods. The summary of our calculations is presented in Table 12. Similarly to the cortisol discussion, the classification was based on the reported detection method and the type of analyte. Table 12 contains studies related to cancer and non-cancer (proteomics, metabolomics, etc.) only.

## 6. Conclusions

Analyzing Table 12 above, one can see that the direct SERS alongside ELISA demonstrated the best performance in terms of balanced accuracy, with 87.4% and 86.2% for SERS and ELISA, respectively. Mass spectrometry (MS) and PCR-based studies followed closely, with balanced accuracy of 83.2% and 84.3% for MS and PCR, respectively. The distribution of methods in terms of overall diagnostic performance, estimated by average AUC values, is almost identical to that by balanced accuracy, with ELISA and Raman-based techniques having exceptionally high discrimination power (AUCs of 0.94 and 0.87 for ELISA, and Raman, respectively), while mass-spectroscopy, PCR, and methods based on other uncategorized methods (IR, Western blotting, etc.) have acceptable discrimination power (AUC withing the range of 0.80–0.84). Based on the limited number of studies considered in our review, other immunoassay-based methods (excluding ELISA) and other non-immunoassay-based methods (electrochemical, IR, Western blotting, etc.) showed the lowest balanced accuracy among all techniques (78.4% and 78.1%, respectively). In terms of AUC, immunoassay-based methods excluding ELISA performed poorest (0.71), whereas other non-immunoassay-based techniques performed comparably to the remaining methods (0.80), with ELISA also showing a suboptimal AUC despite its higher balanced accuracy. This may be related to the fact that immunoassays are already considered the gold standard in clinical diagnostics; consequently, much of the recent research is focused on the development and validation of alternative platforms rather than on improving established immunoassay formats. Similar considerations may apply to studies employing other non-immunoassay-based techniques, many of which remain at earlier stages of clinical translation, with smaller and less standardized study designs. Consequently, newer or less conventional immunoassay-based approaches may currently lack the same degree of optimization, large-scale validation, and clinical standardization as ELISA. It must be noted that direct SERS offers a distinct advantage over ELISA and other immunoassay-based methods for clinical studies, as it does not require antibodies. There is some variability in the results due to the variable affinity of antibodies to antigen, which is often referred to as batch-to-batch variability, as well as the limited stability of immunoreagents, which must be stored at low temperatures and delivered rapidly to preserve their activity. Consequently, direct SERS eliminates the dependence on perishable materials and cold-chain storage. In this case, clinical performance does not rely on temperature-sensitive reagents with the requirement of fast delivery, and it is not exposed to degradation-related variability in binding affinity, which is likely to deteriorate the assay results. However, there is still a limited number of studies done on the clinical applications of surface-enhanced Raman spectroscopy, which we have been able to find (7). SERS still requires standardization, which can focus on the standardization of measurement parameters, including the number of recorded spots in the map, exposure time, laser wavelength, and laser power.

Considering several factors simultaneously (e.g., sensitivity, accuracy, reproducibility, cost, portability, and multiplex detection), electrochemical readout and direct SERS-based platforms may be developed into the next-generation devices owing to their high sensitivity, low cost, portability, and multiplex detection. Similarly to an assessment of other techniques discussed in this review, several factors must be improved for the widespread application of these devices in daily clinical settings. Namely, a decrease in matrix effect, and sometimes a decrease in uncontrolled nanoparticle aggregation for SERS; a decrease in matrix effect and non-specific binding, improvement in transportation and storage of antibodies, and sorting out stability issues for the electrochemical readout methods. To overcome these problems, we discussed several possible solutions in this review, such as optimizing the experimental procedure (e.g., incubation time, the choice of buffer, and blocking solution), introducing surface coatings to nanoparticles and the sensor surface to prevent aggregation, and, at the same time, addressing stability issues.

Overall, we believe that this work will be a valuable contribution to saliva-centered research and influence the development of non-invasive diagnostic strategies in the future.

## Figures and Tables

**Table 1:** The analytical detection of viruses and bacteria in saliva.

Year, First Author; [Ref.]	Technique	[Virus/Bacteria] Analyte	More About Detection	LOD	Other Parameters
**PCR**					
2009, Nadin-Davis; [77]	TaqMan-based quantitative RT-PCR	[V] Human rabies virus N gene	2 µL RNA template in a total volume of 25 µL using the Superscript III Platinum One-Step qRT-PCR kit.	0.01 TCID50 EU of RNA	LOQ is 0.05 TCID50 EU
2010, Chen; [78]	Microfluidic RT-PCR	[B] *B. cereus*	Reagents’ storage pouches, on-chip diaphragm valves, a sample mixing chamber, and a nucleic acid isolation chamber.	1000–10,000 cells mL^−1^	ND
2012, Govindarajan; [79]	Microfluidic origami PCR	[B] *E. coli*	DNA extraction; PCR; agarose gel electrophoresis with ethidium bromide staining.	33 CFU mL^−1^	A raw sample-to-result in 2 h
2012, Wang; [80]	PCR/ligase detection reaction/universal array assay	[B] *Mycobacterium tuberculosis*	Cell lysis, solid-phase DNA extraction, PCR, ligase detection reaction, and universal array hybridization.	50 cells	Point of care, results in 30 min
2013, Oblath; [81]	RT-PCR on a microfluidic chip	[B] *methicillin-susceptible Staphylococcus aureus* (MSSA)	An aluminum oxide membrane to extract and concentrate DNA, PCR, and fluorescence imaging.	8–12 copies (30 fg) of gDNA	Detection time of 2.5 h
2016, Krone; [82]	Quantitative PCR of dried saliva spots	[B] *Strepto coccus pneumoniae*	DNA was extracted with the DNeasy Blood & Tissue Kit; real-time quantitative PCR.	25 CFU/reaction	LOD: 6 cfu/reaction in PBS, stability at 20 to 37 °C, up to five weeks
2018, Singh; [83]	ELISA-PCR assay	[B] *M. tuberculosis*-specific purified early secreted antigenic target-6	Magnetic beads-coupled gold nanoparticle-based immuno (ELISA)-PCR assay.	10 fg mL^−1^	LOD in PBS and spiked saliva are the same; LOD is 5 orders of magnitude higher than for ELISA
2018, Wongphutorn; [84]	16S rRNA-based real-time PCR	[B] *Helicobacter pylori*	DNA extraction; PCR; agarose gel electrophoresis; the results were analyzed using CFX Manager Version 3.1 by staining with ethidium bromide.	10 cells mL^−1^	ND
	vacA-based semi-nested PCR			10 cells mL^−1^	ND
2020, Kim; [85]	Integrated microfluidic preconcentration and nucleic amplification system (µFPNAS) and PCR	[V] Influenza A Virus H1N1	µFPNAS, consisting of a trapezoidal preconcentration microchamber and an RT-PCR chamber.	100 TCID50 (50% tissue culture infective dose)	Detection within 2 h
2020, Li; [86]	PCR	[B] *Lactobacillus casei*	Genomic DNA extraction from bacterial cultures by bead-beating lysis or chemical lysis; automated purification; quantitative PCR.	40 CFU mL^−1^	ND
		[B] *Streptococcus mutans*		1000 CFU mL^−1^	ND
		[B] *Aggregatibacter actinomycetemcomitans*		700 CFU mL^−1^	ND
2020, Silva; [87]	10, 20-pool RT-PCR	[V] Human cytomegalovirus (HCMV)	Pools of 10 saliva samples (10-pool) and individual testing; agreement between qualitative and quantitative results.	150 IU mL^−1^	Cost reduction close to 85% (10-pool)
2020, Zhu; [88]	Microfluidic rt-PCR	[V] Zika Virus	(1) four chitosan-modified silicon dioxide capillaries integrated in the microfluidic chip for target ZIKV RNA enrichment and “in situ PCR” amplification.	50 transducing units (TU) mL^−1^	LOD of 2.5 × 103 genome equivalents mL^−1^
2021, Hernandez; [89]	RT-PCR/MALDI-TOF mass spectrometry (Agena MassARRAY^®^)	[V] SARS-CoV-2	RNA extraction; RT-PCR with iPLEX^®^ Pro chemistry; SpectroCHIP^®^ Array (silicon chip with prespotted matrix crystal; MassARRAY^®^ Analyzer (a MALDI-TOF mass spectrometer).	1562 copies mL^−1^	Sensitivity: 97.14% (n = 34), specificity: 100% (n = 26)
2021, Nishibata; [90]	Real-time one-step PCR	[V] SARS-CoV-2 RNA	Real-time one-step PCR was performed using Applied Biosystems^®^ StepOnePlus.	5000 copies mL^−1^	LOD in PBS was 5000 copies mL^−1^
2021, Sun; [91]	QuantiVirus™ RT-quantitative PCR	[V] SARS-CoV-2 RNA (N, Orf1ab, E genes)	The Bio-Rad CFX 384 instrument was used.	100 copies/reaction	Sensitivity: 94.8%, specificity: 100%
2022, Neopane; [92]	TaqMan RT-PCR multiplex assay	[V] SARS-CoV-2	RNA extraction through the Omega Bio-Tek Mag-Bind Viral RNA Xpress Extraction Kit.	500 copies/reaction	ND
		[V] Respiratory syncytial virus (RSV)		100 copies/reaction	ND
		[V] Influenza		500 copies/reaction	
**LAMP**					
2016, Song; [93]	RT-LAM	[V] Zika Virus	RNA was extracted with Qiagen Viral RNA mini kit; amplification with PeltierThermal Cycler PTC-200 at 63 °C.	50–100 PFU mL^−1^	The cost of the test was $2. Detection in 40 min
2017, Du; [94]	LAMP with programmable toehold-mediated strand-exchange signal transduction, and standard pregnancy test strips	[V] Ebola Virus	(1) LAMP; (2) capture of a hCG (human chorionic gonadotropin)–DNA conjugate by the LAMP amplicon; and (3) readout using a pregnancy test strip.	20 copies	The storage is for at least 90 days without losing activity
2017, Loo; [95]	Microfluidic platform lab on a disk with RT-LAMP	[B] *Mycobacterium tuberculosis* (TB)	DNA extraction by sample heating with chemical lysis buffer and silica microbeads. The amplification with SYTO-9 as the signal reporter.	1000 CFU mL^−1^	Diagnosis in 2 h
		[B] *Acinetobacter baumanii* (Ab)		100 CFU mL^−1^	
2017, Priye; [96]	QUASR RT-LAMP box with smartphone	[V] Zika Virus	Quenching of unincorporated amplification signal reporters (QUAS) technique; Incubation at 67 °C for 40 min.	100 pfu mL^−1^	LOD in the Tris buffer = 22PFU mL^−1^; reaction run for 40 min
2018, Castro; [97]	LAMP	[V] Zika Virus	RNA detection with capsid-specific primers, real-time amplification at 65 °C for 30 min.	6.6 RNA copies/reaction	Test in less than 30 min; time to positivity: 13.5 min
2018, Jiang; [98]	RT-LAMP	[V] Zika Virus	Valve-enabled lysis, paper-based RNA enrichment, and RNA amplification device; colorimetric detection.	0.5 PFU	Detection within 50 min
2018, Sabalza; [99]	Microfluidic RT-LAMP and reverse dot-blot for detection	[V] Zika Virus	A novel reverse dot-blot hybridization technique to monitor the amplicons from the RT-LAMP assay.	2200 RNA copies mL^−1^ (6.6 RNA/reaction)	Simultaneous detection of 24 samples
2021, Bangpanwimon; [100]	Colorimetric magnetic LAMP	[B] *Helicobacter pylori*	Carboxylated magnetic nanoparticles were immobilized with the amino-modified primer; Amplification; mixing with NeutrAvidin-horseradish peroxidase conjugate, and an ABTS (colorless) substrate.	40 CFU mL^−1^ (0.2 CFU/reaction)	100 times more sensitive than PCR, LAMP
2021, Janíková; [101]	RT-LAMP	[V] SARS-CoV-2 RNA	No RNA extraction, sample pretreatment by incubation, and adding RNAse inhibitor, Chelex100 solution.	6 copies/reaction	100% in accordance with RT-PCR for 10 samples
2021, Lalli; [102]	Colorimetric assay using RT-LAMP	[V] SARS-CoV-2 RNA	Pre-treatment by heating (no RNA extraction). Reaction on a QuantStudio 3 or 6 RT-PCR system.	59 copies/reaction	90% in accordance with RT-PCR for 30 samples
2021, Sohrabi; [103]	LAMP	[V] Helicobacter pylori	The optimal temperature and time were determined to be 65 °C for 60 min.	0.25 fg µL^−1^	N = 50 (21+; 29−). Sensitivity/specificity/accuracy of 58.1%/84.2%/68%
2021, Yamazaki; [104]	Point-of-care RT-LAMP	[V] SARS-CoV-2 RNA	Extracting RNA from saliva samples using semi-alkaline proteinase, triplex RT-LAMP, and colorimetric readout.	250 copies/reaction; 25,000 cp mL^−1^	N = 44 (23+; 21-). Sensitivity/specificity 82.6/100%. Detection in 45 min
2024, Kim; [105]	Sweetened displaced probes (DP)-LAMP in mosquito saliva	[V] West Nile (WNV) and Dengue (DENV-I) viruses RNA	Inducing mosquito feeding and salvation using aqueous sucrose (up to 48 h, stabilized viral RNA), followed by DP-LAMP and colorimetric visualization, results in 45 min.	10^−2^ PFU of WNV and 10^−3^ PFU of DENV-I,	Specificity of 92% for WNV and 85% for DEN-I in agreement with RT-qPCR
2024, Naranbat; [106]	Point-of-care RT-LAMP lateral flow biosensor	[V] SARS-CoV-2 RNA, N2 gene	Without RNA isolation, a syringe-based point-of-care automatic system, duplex RT-LAMP followed by a lateral flow strip.	500 copies mL^−1^	Uses 200 µL sample
**Other nucleic acid amplification-based**					
2009, Nugent; [107]	APTIMA HIV-1 RNA qualitative assay (FDA approved)	[V] HIV-1	Target capture-based sample preparation; transcription-mediated amplification; chemiluminescent detection via a hybridization protection assay.	25.7 copies mL^−1^	ND
2015, Du; [108]	Coupling isothermal amplification and strand exchange circuits to a glucometer	[V] MERS-CoV (Middle East respiratory syndrome coronavirus)	Loop-mediated isothermal amplification (LAMP) combined with nucleic acid circuitry to transduce virions into signals on a commercial glucometer.	20–100 copies µL^−1^	ND
2021, Biyani; [109]	RNA isothermal co-assisted and coupled amplification	[B] *E. Coli*	Adding RNA stabilizing reagent and heating the saliva sample. One pot RICCA reaction at 37 °C for 15 min, and then lateral flow detection.	105 copies of RNA templates	8.6 CFU µL^−1^; cost for RNA reagents: USD 2.6 per reaction; for DNA-specific: USD 1.3 per reaction
**Electrochemical**					
2013, Zhang; [110]	Magnetic electrochemical biosensor; chronoamperometry	[V] Single-nucleotide polymorphisms of trace HIV-related salivary DNA	Electrically controllable magnetic gold electrode.	0.37 fM	LR: 1–100 fM, R^2^= 0.9951, recovery: 96–106%
2016, Hoyos-Nogués; [111]	Peptide-based electrochemical impedance spectroscopy.	[B] *Strepto coccus sanguinis*	Sensors biofunctionalized with this antimicrobial peptide) sense the presence of pathogenic S.sanguinis, translating this interaction into measurable impedimetric signals.	860 CFU mL^−1^	LR: 10–10,000 CFU mL^−1^; detection in 1 h; LOD of 35 CFU mL^−1^ in KCl
2017, Nidzworski; [112]	Electrochemical impedance spectroscopy at nano-scale boron-doped diamond biosensors	[V] Influenza virus M1 protein	A diamond electrode functionalized with polyclonal anti-M1 antibodies, to identify the M1 protein influenza biomarker.	1 fg mL^−1^	Incubation time less than 5 min at room temperature
2017, Yang; [113]	Cyclic voltammetry using whole-cell redox reactivation/cycling	[B] *Pseudo-monas aeruginosa biomarker pyocyanin*	The electroactive bacteria-mediated redox reactivation module was constructed using Shewanella oneidensis MR-1 cells as the bioelectro-catalyst and lactate as the electron donor.	47 pM	Over 85% signal retained after 1 week of storage at 4 °C
2018, Chekin; [114]	Aptamer-based differential pulse voltammetry	[V] Human papillomavirus L1 protein	NH_2_-functionalized RNA aptamer, Sc5-c3, was immobilized onto the electrode surface and [Fe(CN)_6_]_4_− as a redox mediator.	0.1 ng mL^−1^	Linear range: 0.2–2 ng mL^−1^
2019, Bhardwaj; [115]	Electrochemical impedance spectroscopy-based immunosensor	[V] Influenza H1N1	Sandwich immunoreactions of HRP-Ab-H1N1-Ab were performed on the gold paper electrode of the immune strip.	4.7 PFU mL^−1^	ND
2020, Joshi; [116]	Electrochemical impedance spectroscopy	[V] Influenza H1N1	Graphene oxide electrode flakes were drop-cast onto indium tin oxide/glass electrodes to fabricate a label-free immunosensor.	33 plaque-forming unit	ND
2021, Fabiani; [117]	Differential pulse voltammetry immunassay	[V] SARS-CoV-2 nucleocapsid protein	The electrochemical assay uses magnetic beads as support for the immunological chain and a secondary antibody with alkaline phosphatase as an immunological label.	8 ng mL^−1^	Analysis time of 30 min; 91.7% in accordance with PCR
		[V] SARS-CoV-2 spike protein		19 ng mL^−1^	
2021, Rashid; [118]	Electrochemical sensor	[B] *Pseudomonas aeruginosa biomarker pyocyanin*	Electrode-graphene oxide decorated with gold nanoparticles.	1.34 µM	LR: 1–100 µM; LOD in PBS: 0.27 µM; in urine: 2.3 µM
2022, Maddocks; [119]	Square wave voltammetry aptasensor.	[V] Influenza A H1N1 hemagglutinin	The aptamer was derivatized with thiol modification at the 5′-terminus and methylene blue modification at the 3′-terminus to enable conjugation to the electrode.	1.5 pM	linear detection of up to 1.2 nM
2025, Uddin; [120]	Polymyxin-B functionalized chitosan-coated magnetic nanobeads	*E. coli*	PolyB-CS-MNBs demonstrated a remarkable capacity to pre-concentrate E. coli in saliva, effectively lowering the limit of detection (LOD) up to 10 cfu mL^−1^ when coupled with polymerase chain reaction (PCR).	10 cfu mL^−1^	30 min incubation time, cap efficiency > 95%
2025, Xing; [121]	Differential pulse voltammetry electrochemical immunosensor	H7N9 virus hemagglutinin protein	Platform fabricated from graphene oxide, chitosan, and nanogold assembly, utilizing anti-H7N9 antibody Fab fragments	0.28 pg mL^−1^	5 min response time, range: 1.5–25 pg mL^−1^, R^2^ = 0.99, RSD < 5%, recovery: 94–112%
**Chemiluminescent**					
2009, Nugent; [107]	APTIMA HIV-1 RNA qualitative assay (FDA approved)	[V] HIV-1	Target capture-based sample preparation; transcription-mediated amplification; chemiluminescent detection via a hybridization protection assay.	25.7 copies mL^−1^	ND
2022, Hunt; [122]	Paper-based cell-free toehold switch biosensor	[V] SARS-CoV-2 RNA	Lyophilized cell-free protein synthesis and toehold switch riboregulators are employed; bioluminescent reporter protein NanoLuc is used.	10 nM	The estimated cost is less than 0.5 USD; point-of-care device
2022, Prainito; [123]	Colorimetric paper-based polydiacetylene (PDA) biosensor	[V] SARS-CoV-2 Spike protein	PDA sensor platform contains the N-Hydroxysuccinimide ester of 10, 12-pentacosadiynoic acid (PCDA-NHS); colorimetric output.	1 ng mL^−1^	Detection range of 1 to 100 ng mL^−1^
**SERS**					
2017, Žukovskaja; [124]	Microfluidic surface-enhanced Raman spectroscopy	[B] *Pseudomonas aeruginosa biomarker pyocyanin*	The saliva samples were filtered using a cellulose acetate membrane, with Ag nanoparticles as a substrate.	10 µM	LOD in aqueous solution: 0.5 uM; LR: 0.5–15 uM
2019, Eryılmaz; [125]	SERS lateral flow immunoassay	[B] Group *Streptococcus pyogenes*	Magnetic Au nanoparticles, Au nanorods with SERS tag (5,5′-dithiobis (2-nitrobenzoic acid)), 785 nm laser source.	0.2 CFU mL^−1^	LOD is one cell
2021, Zhang; [126]	Surface-enhanced Raman scattering-based immunoassay	[V] SARS-CoV-2 spike protein	SERS-immune substrate was fabricated by a novel oil/water/oil (O/W/O) three-phase liquid–liquidliquid-liquid interface self-assembly method.	6.07 fg mL^−1^	Range: 10 fg mL^−1^ to 10 ng mL^−1^; LOD for serum: 7.60 fg mL^−1^; blood: 0.10 pg mL^−1^; PBS: 0.77 fg mL^−1^
2023, Atta; [127]	SERS	[B] *Pseudomonas aeruginosa biomarker pyocyanin*	Surfactant-free gold nanostars as substrates.	0.4 nM	LOD in water: 0.05 nM
2024, Zhang; [128]	SERS	[B] *Porphyromonas gingivalis*	Substrate: Ag nanoparticles; Reducing agent: sodium borohydride; aggregating agent: Na+.	10 × 10^5^ CFU mL^−1^	Linear discriminant analysis applied for specificity
**Other**					
2012, Bañuls; [129]	Sandwich microimmunoassay on a standard DVD reader for quantification	[V] Influenza A (H1N1 and H3N2)	The polycarbonate surface chemical modification of commercial DVDs to covalently attach influenza capture antibodies: monoclonal capture antibody, monoclonal detection antibody, and GAR antibody 1 in carbonate buffer as an internal negative control.	29 ng mL^−1^	Cost of detector 1 k€; 30 min test time; portable, naked-eye detection
2012, Mannoor; [130]	Peptide–graphene nanosensors	[B] *Helicobacter pylori*	Graphene nanosensors are comprehensively printed onto water-soluble silk thin-film substrates.	100 cells µL^−1^	Assay time 15 min
2019, Li; [131]	Fluorescent SiC@BSA nanoprobe biosensor	[V] *Streptococcus salivarius*	SiC@BSA NPs conjugated with antimicrobial peptide GH12; fluorescence quenching at 320/410 nm upon bacteria binding.	25 CFU mL^−1^	Signal retained after 12 weeks, LR 1.0 × 10^2^ to 1.0 × 10^7^ CFU mL^−1^

Abbreviations: LOD—limit of detection; LOQ—limit of quantification; PCR—polymerase chain reaction; RT—reverse transcription; EU—equivalent units; LAMP—loop-mediated, isothermal amplification; LR—linear range; HIV—human immunodeficiency virus.

**Table 2:** The summary of clinical studies on the detection of viruses and bacteria in saliva using electrochemical/electrochemiluminescence, ELISA, PCR, LAMP, and Raman/fluorescence methods. Studies with a sample size of fewer than 20 were excluded from calculations of average and median sensitivity, specificity, and accuracy, and are marked with an asterisk (*).

Year, First Author; [Ref.]	Method	Analyte	Sensitivity	Specificity	Accuracy	Other FOM
**Electrochemical/Electrochemiluminescence**		**Average**	**92.1%**	**95.6%**	**94.4%**	
		**Median**	**93.3%**	**100.0%**	**92.7%**	
2015, Griffin; [141]	Meso Scale Discovery (MSD) electrochemiluminescence immunoassays	Norwalk virus IgG	100%	100%	100%	N = 20
2015, Ishii; [142] *	Antigen–antibody reaction-assisted dielectrophoretic impedance measurement	*Tannerella forsythia*	76.50%	100%	79.00%	N = 19 (17+; 2−)
2021, Fabiani; [117]	Electrochemical immunoassay	SARS-CoV-2 S (spike) protein	100%	88.23%	91.67%	N = 24 (7+; 17−)
2021, Liv; [143]	Cyclic voltammetry sensor	SARS-CoV-2	93.30%	92.50%	92.70%	N = 110 (30+; 80−)
2021, Zhang; [144]	Aptamer-based electrochemical impedance sensor	SARS-CoV-2 B.1.1.7 variant	80.50%	100%	90.40%	N = 73 (36+; 37−)
2022, Liv; [145]	Square wave voltammetry sensor	SARS-CoV-2	85%	90%	87.50%	N = 40 (20+; 20−); LOD = 9.3 ag mL^−1^; detection in 20 min
2022, Najjar; [137]	Microfluidic LAMP, CRISPR, and electrochemical sensor	SARS-CoV-2 Orf1a RNA	100%	100%	100%	N = 30 (19+; 11−)
2022, Victorious; [146] *	Cyclic voltammetry electrochemical	Porcine epidemic diarrhea viruses	83.30%	100%	91.70%	N = 12 (6+; 6−); LOD = 9 nM; in 1 h
2024, Sen; [147]	Electrochemical (impedance) sensor with trimeric aptamer	SARS-CoV-2 S protein	100%	100%	100%	N = 37 (10+; 27−)
2025, Garcia-Junior; [148]	Cyclic voltammetry electrochemical sensor with a neural network for analysis	SARS-CoV-2	90%	90%	90%	N = 20 (10+; 10−); COVID-19 symptomatic patients
2025, da Silva [149]	Impedimetric electrochemical biosensor	H. pylori CagA oncogenic protein	80%	100%	97%	N = 20 (15+, 5–), LOD: 109.9 fg mL^−1^, detection range: 0.5 pg mL^−1^ to 3.3 ng mL^−1^, 15 min response time
**ELISA**		**Average**	**94.8%**	**95.6%**	**94.7%**	
		**Median**	**98.2%**	**100.0%**	**97.9%**	
2015, Lazutka; [150]	ELISA IgA	Schmallenberg virus	98.20%	ND	ND	N = 55 (54+; 1−)
2015, Sattler; [151]	Cotton gauze swabs ELISA	Reproductive and respiratory syndrome Virus	100%	97.40%	97.70%	N = 350 (311+; 39−)
2016, Bjustrom-Kraft; [152]	Oral fluid IgA whole virus ELISA	Porcine epidemic diarrhea virus (PEDV)	100%	100%	100%	N = 88 (52+; 36−)
2017, Senthilkumaran; [153]	Isotype-specific indirect ELISA	Swine vesicular disease virus (SVDV)	100%	100%	100%	N = 211 (12+; 199−)
2018, Gadekar; [154]	ELISA	*Aggregatibacter actinomycetemcomitans*	84%	76%	81%	N = 100 (50+, 50−); AUC = 0.95
2020, MacMullan; [155]	ELISA	SARS-CoV-2	84.20%	100%	91.30%	N = 149 (82+; 67−); AUC = 0.98
2020, de Oliveira; [156]	ELISA	*Trypanosoma cruzi*	97%	100%	98%	N = 150 (100+; 50−); AUC = 0.96
**Polymerase Chain Reaction (PCR)**		**Average**	**90.1%**	**98.9%**	**95.8%**	
		**Median**	**90.8%**	**100.0%**	**96.7%**	
2015, González Mediero; [157]	PCR (Mycobacterium tuberculosis direct test)	*Mycobacterium tuberculosis*	71.80%	95%	80.80%	N = 52 (32+; 20−)
2016, Sueki; [158]	RNA extraction and droplet RT-PCR	Influenza	87.50%	98.20%	95.80%	N = 144 (32+; 112−)
2018, Lee; [159]	Direct PCR with an immunochromatographic strip	*Streptococcus sanguinis*	90%	ND	ND	N = 10+
		*Streptococcus salivarius*	100%	ND	ND	N = 10+
2018, Liu; [160]	PCR and DNAzyme-catalyzed reaction	*Helicobacter pylori*	100%	100%	100%	N = 20 (11+,9−)
2018, Park; [161]	DNA extraction PCR	Varicella-zoster virus	88.50%	100%	92.30%	N = 82 (52+; 30−)
2019, Pillman; [162]	DNA extraction and real-time PCR	*Mycoplasma hyorhinis*	97.30%	ND	ND	N = 37+
2019, To; [163]	Xpress Flu/RSV assay, monoplex RT-PCR	Influenza (A/B), Respiratory syncytial virus	90.80%	100%	96.70%	N = 212 (76+; 136−)
2020, DeMuri; [164]	Real-time PCR	Streptococcus	95%	ND	ND	N = 20+
2020, Silva; [87] *	10, 20-pool RT-PCR	Human cytomegalovirus (HCMV)	100%	ND	ND	N = 12 (12+)
2020, Vaz; [165]	RNA isolation, real-time RT-PCR	SARS-CoV-2	94.40%	97.60%	96.10%	N = 155 (71+; 84−)
2021, Al Suwaidi; [166]	RNA extraction RT-PCR	SARS-CoV-2	87.70%	98.50%	96.70%	
2021, Dollard; [167]	PCR	Congenital cytomegalovirus	92.90%	99.90%	99.90%	N = 12,554 (56+; 12,498−)
2021, Galar; [168]	RT-PCR (Xpert Xpress Flu/respiratory syncytial virus test)	Influenza (A/B)	89.50%	100%	97.60%	N = 82 (19+; 63−)
2021, Hernandez; [89]	RT-PCR/MALDI-TOF mass spectrometry (Agena MassARRAY^®^)	SARS-CoV-2	97.10%	96.00%	96.70%	N = 60 (34+; 26−)
2021, Marx; [169]	Real-time RT-PCR	SARS-CoV-2	85.20%	98.60%	97.20%	N = 496; (54+, 442−)
2021, Sun; [91]	QuantiVirus™ (RT-qPCR) SARS-CoV-2 test	SARS-CoV-2 RNA (N, Orf1ab, E genes)	94.80%	100%	97.00%	N = 131 (77+; 54−)
2021, Tutuncu; [170]	RT-PCR	SARS-CoV-2	90.56%	ND	ND	N = 53+
2021, Yee; [171]	Quantitative RT-PCR	SARS-CoV-2	81.40%	100%	91.00%	N = 300
2022, Idrissi Janati; [172]	DNA extraction qPCR	*Fusobacterium nucleatum*	95.30%	ND	ND	N = 43+
2022, Kiryanov; [173]	Direct RT-qPCR (without RNA extraction)	SARS-CoV-2	94.60%	100%	96.70%	N = 61 (37+; 24−)
2022, Neopane; [92]	TaqMan SARS-CoV-2, Flu A/B, RSV RT-PCR multiplex assay	SARS-CoV-2	100%	100%	100%	N = 75 (35+; 40−)
2024, Kim; [174]	Proteinase K method/RT-qPCR	SARS-CoV-2	94%	100%	99%	N = 251 (51+, 200–), nasopharyngeal aspirate samples comparison
2025, Ramirez; [175]	RT-PCR	Influenza	68%	ND	ND	N = 60 (60+)
		Respiratory syncytial virus (RSV)	75%	ND	ND	
2025, Varughese; [176]	PCR	Influenza A Virus	90%	98%	95%	N = 149 (58+, 91–), agreement percentage with nasopharyngeal swab
**Loop Mediated Isothermal Amplification (LAMP)**		**Average**	**90.7%**	**99.3%**	**94.3%**	
		**Median**	**90.7%**	**100.0%**	**94.6%**	
2021, Janíková; [101] *	RT-LAMP	SARS-CoV-2 RNA	100%	100%	100%	N = 10 (3+; 7−)
2021, Lalli; [102]	Colorimetric RT-LAMP	SARS-CoV-2 RNA	85%	100%	90%	N = 30 (20+; 10−)
2021, Toppings; [177]	Saliva-Dry RT-LAMP	SARS-CoV-2	100%	96.70%	98.30%	N = 60 (30+; 30−)
2021, Yamazaki; [104]	Point-of-care RT-LAMP	SARS-CoV-2 RNA	82.60%	100%	90.90%	N = 44 (23+; 21−)
2022, Lim; [178] *	Microfluidic RT-LAMP	SARS-CoV-2 B.1.1.7 variant	90%	100%	ND	N = 26 (20+; 6−); AUC = 1
		SARS-CoV-2 early strains	90.90%	100%	ND	N = 18 (11+; 7−); AUC = 0.96
2022, Najjar; [137]	Microfluidic LAMP, CRISPR, and electrochemical sensor	SARS-CoV-2 Orf1a RNA	100%	100%	100%	N = 30 (19+; 11−); AUC = 1
2024, Xiao; [179]	RT-LAMP CRISPR-Cas12 mediated DNAzyme actuator	SARS-CoV-2	96.30%	100%	98.60%	N = 207 (81+; 126−)
2025, Šušnjar; [180]	Colorimetric RT-LAMP	SARS-CoV-2	80.00%	99%	88.00%	N = 577 (340+, 237–), clinical LOD 58 genome copies per µL, 30 min assessment time
**Raman/Fluorescence**		**Average**	**99.4%**	**98.5%**	**99.0%**	
		**Median**	**100.0%**	**100.0%**	**100.0%**	
2015, Griffin; [141]	Luminex fluorometric immunoassay	Norwalk virus IgG	100%	100%	100%	N = 20
2017, Pisanic; [181]	Luminex-based immunoassay with magnetic beads	Hepatitis E virus IgG	98.70%	98.40%	98.60%	N = 141 (76+; 65−)
2020, Desai; [182]	Raman spectroscopy	RNA virions	92.50%	88.80%	91.60%	N = 185 (54+; 131−)
2022, Yang; [183]	SERS (using Support Vector Machines), Ag nanorod @ SiO_2_ substrate. Both training and testing set spectra are included, with a 7:3 ratio.	Human coronavirus NL63 (CoV NL63)	100%	100%	100%	N = 15,600 (1134+; 14,466−)
		Human coronavirus 229E (CoV 229E)	100%	100%	100%	N = 15,600 (749+; 14,851−)
		Human coronavirus OC43 (CoV OC43)	99.70%	100%	99.97%	N = 15,600 (1218+; 14,382−)
		Influenza A H1N1	100%	99.97%	99.97%	N = 15,600 (1315+; 14,285−)
		Influenza A H3N2	99.60%	100%	99.97%	N = 15,600 (939+; 14,661−)
		Influenza B (IBV)	100%	100%	100%	N = 15,600 (1174+; 14,426−)
		Respiratory syncytial virus strain A2 (RSV-A2)	100%	100%	100%	N = 15,600 (1304+; 14,296−)
		Respiratory syncytial virus strain B1 (RSV-B1)	100%	100%	100%	N = 15,600 (1417+; 14,183−)
		Human metapneumovirus strain A (HMPV-A)	100%	100%	100%	N = 15,600 (1579+; 14,021−)
		Human metapneumovirus strain B (HMPV-B),	100%	100%	100%	N = 15,600 (1531+; 14,069−)
		Adenovirus type 5 (Ad5)	100%	99.97%	99.97%	N = 15,600 (1740+; 13,860−)
2023, Karunakaran; [184]	SERS with Support Vector Machine (SVM)	SARS-CoV-2	100%	90%	95%	N = 60 (30+; 30−)

**Table 3:** Detection of biomarkers in saliva using mass spectrometry.

Year, First Author; [Ref.]	Method	Analyte	Reported LOD	LOD, M	Other (Statistical Method, AUC, ROC)
**Mass Spectrometry**			**Geometric Average**	**7.05 × 10^−10^**	
			**Median**	**1.18 × 10^−9^**	
2010, Gherardi; [224]	GC/MS	toluene	0.22 ng mL^−1^	2.39 × 10^−9^	Accuracy: 99–105%, RSD: 1.7–13.8%
2014, Wang; [225]	UPLC–ESI–MS	l-phenylalanine	3.9 ng mL^−1^	2.36 × 10^−8^	L-phenylalanine: R^2^ = 0.995, LOQ: 13 ng mL^−1^, precision range 3–4.3%
2015, Cheng; [27]	UPLC-MS	17 kinds of SFAAs (Thr, Ser, Glu)	0.94 ng mL^−1^	6.30 × 10^−9^	LOD: 0.94–237.62 ng mL^−1^, LOQ: 3.14–792.07 ng mL^−1^; R^2^ = 0.99; recoveries: 95.4–106.2%
			237.62 ng mL^−1^	1.79 × 10^−6^	
2016, Kawahara; [226]	Selected reaction monitoring MS (SRM/MS)	14 proteins, VSEADSSNADWVTK, YGLVTYATYPK	0.90 fmol µL^−1^	9.00 × 10^−10^	LOQ: VSEADSSNADWVTK: 2.7 fmol/µL; YGLVTYATYPK: 5.5 fmol/µL; R2 = 0.99; CVs: 7.5–25%, (C1R, TINAG, SLPI, SERPINE1, LRG1, LCN2, TAGLN2, ANXA1, FAM49B, CFB, C4B, C3, SERPINA1 and SAA1)
			1.8 fmol µL^−1^	1.80 × 10^−9^	
2016, Hirtz; [222]	MRM-MS	35 plasma proteins, Coagulation factor XII, Clusterin, Kininogen	0.9 pg mL^−1^	1.00 × 10^−14^	LOD: 0.9–481.3 pg mL^−1^, LOQ: 1.0–2301.9 pg mL^−1^, average CV—0.47%, average analytical variability—4.6%, and total variability—8.5%
			481.3 pg mL^−1^	7.29 × 10^−12^	
2017, Hsiao; [227]	Stable isotope standards with capture by anti-peptide antibodies coupled with multiple reaction monitoring MS (SISCAPA-MRM-MS)	24 biomarkers (MMP1, MMP2, TNC)	0.002 fmol mg^−1^	ND	Accuracy: 31.56–171.40%, R^2^ = 0.622–1.000, LOD range: 0.004–0.348 fmole mg^−1^, LLOQ: 0.006–1.044 fmole mg^−1^
			0.348 fmol mg^−1^	ND	
2017, Chen; [228]	Liquid Chromatography Multiple-Reaction-MonitoringMS (LC-MRM/MS)	56 proteins (kininogen 1 (KNG1), A1AT (alpha-1-antitrypsin), AACT (alpha-1-antichymotrypsin)	0.1 amol µg^−1^	1.32 × 10^−10^	LOD: 0.1–5628.0 amol µg^−1^ for 56 proteins; LLOQ: 2.6 amol µg^−1^−74 fmol µg^−1^; accuracy 80–120%; CV
			5628 amol µg^−1^	7.04 × 10^−6^	
2020, Hsiao; [37]	immuno-MALDI-MS	MMP1	0.26 fmol	2.60 × 10^−16^	Precision (intra-day and inter-day variation < 10%), accuracy (80–100%), and sensitivity (LOQ at 3.07 ng mL^−1^)
2020, Lin; [38]	MALDI-TOF-MS	MMP1	6.75 ng mL^−1^	1.25 × 10^−10^	Average recovery of 71%; R^2^= 0.9998
2021, Grau; [229]	LC-MS/MS	cortisone,	0.018 ng mL^−1^	4.99 × 10^−11^	Linear range: 0.3–20 ng mL^−1^, RSD: 4.2–10%, recovery: 86–111%
		cortisol	0.029 ng mL^−1^	8.00 × 10^−11^	
2021, Zhang; [230]	HILIC-UPLC-HRMS	L-valine (Val)	0.17 ng mL^−1^	1.45 × 10^−9^	Precision, linearity (R^2^ > 0.99), recovery (92.2–110.3%), intra- and inter-day precision (RSD < 7% and RSD < 9%, respectively); sensitivity: 91.2%; specificity: 85.2%; LOQ: 0.57–27.16 ng mL^−1^
		L-glycine (Gly)	8.15 ng mL^−1^	1.09 × 10^−7^	
2025, Adelaars; [231]	LC-MS/MS	creatinine	0.42 µmol L^−1^	4.20 × 10^−7^	LLOQ: 1.26 µmol L^−1^, accuracy: 93.9–97.8%, imprecision: 3.4–8.1%, linearity: 2–100 µmol L^−1^
2025, Pelcová; [232]	LC-MS	lamotrigine	0.02 µg mL^−1^	7.81 × 10^−8^	LOQ: 0.06 µg mL^−1^, precision: 2.1–6.1%, accuracy: 96–111%, recovery: 90–93%
2025, Peris-Pastor; [233]	LC-MS/MS	8OHdA	0.03 ng mL^−1^	1.12 × 10^−10^	LOQ: 0.10 ng mL^−1^, RSD: 3–13%, recovery: 90–119%, range up to 20 ng mL^−1^, R^2^ = 0.996
		8OHdG	0.05 ng mL^−1^	1.77 × 10^−10^	LOQ: 0.10 ng mL^−1^, RSD: 4–13%, recovery: 83–111%, range up to 20 ng mL^−1^, R^2^ = 0.997

**Table 4:** Analytical studies on saliva detection using optical-based immunoassay/assay methods.

Year, First Author; [Ref.]	Method	Analyte	Biomarker for	LOD, M	Other FoM
**Optical-Based Immunoassay/Assay Methods**			**Geometric Average**	1.06 × 10^−12^	
			**Median**	7.50 × 10^−13^	
2014, Jenko; [249]	ELISA-based protein antibody microarray	BoNT/A	Toxin	2.20 × 10^−14^	Assay time: 12 h (can be lowered to 2 h with the increase in LOD)
		BoNT/B		8.50 × 10^−15^	
		BoNT/C		1.40 × 10^−13^	
		BoNT/D		8.64 × 10^−13^	
		BoNT/E		2.28 × 10^−14^	
		BoNT/F		2.16 × 10^−12^	
		Ricin		4.92 × 10^−12^	
		Stx-1, Stx-2		1.17 × 10^−13^	
2016, Zhang; [251]	ELISA	Galectin 3	Heart failure	8.00 × 10^−11^	Rec.: 109–110%
2018, Apilux; [252]	Lateral flow IA	Cortisol	Stress level	1.38 × 10^−9^	LR 0.5–150 ng mL^−1^, assay time: 15–20 min
2018, Chakraborty; [41]	ELISA	OPN	Oral cancer	4.54 × 10^−13^	Range of detection: 0.31–20 ng mL^−1^
2018, Oh; [253]	Trap lateral flow assay	Cortisol	Stress level	2.73 × 10^−11^	LR: 0.01–100 ng mL^−1^
2019, Abdulsattar; [254]	Chemiluminescence IA	Cortisol	Stress level	1.30 × 10^−12^	LR: 0–50 ng mL^−1^, Rec.: 91%
2019, Olczak; [255]	ELISA	MAPT	Traumatic brain injury	2.57 × 10^−13^	ND
2021, Bellagambi; [256]	ELISA	NT-proBNP	Heart failure	1.18 × 10^−13^	LR: 1–200 pg mL^−1^; Rec.: 95–110%
2021, Salarić; [39]	ELISA	MLT	Oral cancer	1.29 × 10^−12^	AUC: 0.84
2023, Kim; [257]	Multicolorimetric ELISA	IL-1β	Periodontal disease	3.77 × 10^−15^	LR: 0–250 pg mL^−1^; Rec.: 100.9%
2024, Moulahoum; [258]	Laser printed-paper-based ELISA	Alpha-fetoprotein	Hepatocellular Carcinoma, Down syndrome	1.43 × 10^−11^	LR: 1.0–800 ng mL^−1^
2025, Kim; [259]	Lateral flow IA	Cortisol	Stress level	1.05 × 10^−11^	LR: 0.01–10 ng mL^−1^
2025, Kim; [260]	Competitive Lateral Flow Assay with colorimetric sensing based on AuNPs	Cortisol	Stress	4.06 × 10^−9^	Rec.: 90%, R^2^ = 0.99, 2 min. assay time, no external equipment
2026, Xiang; [261]	Digital microfluidic CRISPR-Cas IA (fluorescence)	CEA	Tumor	5.56 × 10^−14^	Rec.: 97.0–102.0%, R^2^ = 0.95
		MMP-1		~6.35 × 10^−13^	Rec.: 89.0–103.0%, R^2^ = 0.94

Abbreviations: BoNT/A–F, botulinum neurotoxins; Stx-1 and Stx-2, Shiga toxins 1 and 2; IL-1β, interleukin-1 beta; NT-proBNP, N-terminal pro-brain natriuretic peptide; MAPT, microtubule-associated protein tau; MLT, melatonin; OPN, osteopontin; LR, linear range; Rec., recovery.

**Table 5:** Analytical studies on saliva cancer and non-cancer biomarkers detection using electrochemical methods. Abbreviations: PSA—prostate-specific antigen, IL-interleukin, CYFRA-21-1-Cytokeratin Fragment-21-1, mRNA—messenger ribonucleic acid, LOD-limit of detection, LOQ-limit of quantification, RSD-relative standard deviation, CV—coefficient of variation, N/A—not applicable.

Year, First Author; [Ref.]	Technique	Analyte	Biomarker Type	LOD	LOD (M)	Analytical Range; RSD; Recovery; Other FoM
**Voltammetric Methods (Cyclic Voltammetry, Differential Pulse Voltammetry)**				**Geometric Average**	**1.68 × 10^−12^**	
				**Median**	**5.53 × 10^−13^**	
2016, Kumar; [268]	Immunoelectrode (BSA/anti-CYFRA-21-1/serine/nZrO2/ITO), DPV	CYFRA-21-1	Cancer biomarker	0.0122 ng mL^−1^	3.05 × 10^−13^	0.01–29 ng mL^−1^; 4.4%; N/A; sensitivity at 0.295 mA mL ng^−1^; response time of 6 min
2017, Verma; [269]	Anti-IL8/AuNPs-rGO/ITO Immunosensing Platform; differential pulse voltammetry	IL-8	Cancer biomarker	72.73 pg mL^−1^	8.61 × 10^−12^	500 fg mL^−1^–4 ng mL^−1^; 2.7%; 94.15%; time for detection of 9 min
2018, Chekin; [270]	Label-free electrochemical biosensor; differential pulse voltammetry (DPV)	cTnI	Heart failure biomarker	1 pg mL^−1^	4.17 × 10^−14^	1 pg mL^−1^–100 ng mL^−1^; N/A; N/A; N/A
2018, Pachauri; [271]	BSA/anti-Cyfra-21-1/ncCeO_2_–RGO/ITO immunoelectrode; differential pulse voltammetry	CYFRA-21-1	Cancer biomarker	0.625 pg mL^−1^	1.56 × 10^−14^	0.625 pg mL^−1^–15 ng mL^−1^; N/A; N/A; sensitivity 14.54 µA mL ng^−1^ cm^−2^; R^2^ = 0.98
2019, Farzin; [47]	Immunosensing device, cyclic voltammetry	PSA	Cancer biomarker	2.8 fg mL^−1^	8.36 × 10^−17^	N/A; for single-electrode repeatability = 2.9%, electrode–to–electrode reproducibility = 5.7% (n = 5); N/A; LOQ 9.3 fg mL^−1^; R^2^ = 0.996
2019, Verma; [272]	ZnO–rGO electrode; cyclic voltammetry	IL-8	Cancer biomarker	51.53 pg mL^−1^	6.10 × 10^−12^	N/A; 4%; N/A; sensitivity (12.46 ± 0.82 µA mL ng^−1^)
2020, Chen; [273]	NiCo_2_S_4_-Ms/GCE/DPV; cyclic voltammetry	4-nitroquinoline n-oxide (4-NQO)	Cancer biomarker	2.29 nM	2.29 × 10^−9^	0.005–596.64 µM; 2.56%, 2.76%; 98.24%, 94.29% for 50 nM and 100 nM added
2020, Pachauri; [274]	Silver molybdate nanoparticles-based immunosensor; differential pulse voltammetry (DPV)	IL-8	Cancer biomarker	90 pg mL^−1^	1.06 × 10^−11^	0.001–40 × 10^4^ pg mL^−1^; N/A; N/A; sensitivity: 7.03 µA ng^−1^ mL cm^−2^; response time of 10 min
2020, Zhang; [43]	Electrochemical enzyme biosensor; cathodic stripping voltammetry	Carnitine	Cancer biomarker	0.025 µM	2.50 × 10^−8^	0.025–25 µM; 5.58%; 97.17 and 96.55%; R^2^ = 0.9484
2021, Jalil; [275]	MoS2@rGO nanohybrid sensor; differential pulse voltammetry	EpCAM	Cancer biomarker	44.22 fg mL^−1^	1.11 × 10^−15^	0.001–20 ng mL^−1^; 8.7%; 91.2
2021, Kumar; [276]	APTES/nYZR/ITO biosensing platform; differential pulse voltammetry	CYFRA-21-1	Cancer biomarker	7.2 pg mL^−1^	1.80 × 10^−13^	0.01–50 ng mL^−1^; N/A; N/A; sensitivity: 0.2 mA ng^−1^ mL mm^−2^
2022, Saeed; [277]	Au@TiO_2_/MWCNTs/GCE; differential pulse voltammetry	H_2_O_2_	Cancer biomarker	1.4 µM	1.40 × 10^−6^	200 µM–6 mM; 4.63%.; 91.62–117.9%; R^2^ = 0.9994
2023, Zhang; [278]	Dual-channel electrochemical immunosensor; differential pulse voltammetry (DPV)	IL-1β	Non-cancer biomarker	0.014 ng mL^−1^	8.00 × 10^−13^	0.1–100 ng mL^−1^; N/A; N/A
2024, Gulati; [279]	SPE, CV	CYFRA 21-1	Cancer biomarker	829.5 pg mL^−1^	2.07 × 10^−11^	Detection range: 0–20 ng mL^−1^; sensitivity: 0.935 µA mL pg^−1^ cm^−1^
		IL-8	Cancer biomarker	0.542 pg mL^−1^	6.40 × 10^−14^	Detection range: 0–2000 pg mL^−1^; sensitivity: 0.039 µA mL pg^−1^ cm^−1^
		TP-53	Cancer biomarker	1.165 pg/mL	2.65 × 10^−14^	Detection range: 0–5000 ng mL^−1^; sensitivity: 0.08 µA mL pg^−1^ cm^−1^
**Electrochemical Impedance Spectroscopy**				**Geometric Average**	**1.96 × 10^−14^**	
				**Median**	**2.35 × 10^−15^**	
2016, Choudhary; [40]	Electrochemical immunosensor; electrochemical impedance spectroscopy	CD-59	Cancer biomarker	1.46 fg mL^−1^	1.04 × 10^−16^	1 fg mL^−1^–1000 fg mL^−1^; N/A; 94.6%; sensitivity: 94% up to 5 weeks, then decreased about 11%; detection time: 10 min;
2017, Bellagambi; [280]	Electrochemical impedance spectroscopy	TNF-α	Non-cancer biomarker	3.1 pg mL^−1^	1.79 × 10^−13^	1–100 pg mL^−1^; N/A; N/A; N/A
2018, Aydin; [281]	modified electrochemical immunosensing system by using polymer P3-TMA as an interface material; electrochemical impedance spectroscopy technique (EIS)	IL-1β	Cancer biomarker	3 fg mL^−1^	1.71 × 10^−16^	0.01–3 pg mL^−1^; N/A; 97.4–104.5%; R^2^ = 0.9991%; LOQ: 10 fg mL^−1^
2018, Aydın; [42]	ITO-based electrochemical immunosensor; EIS and cyclic voltammetry	IL-1β	Cancer biomarker	7.5 fg mL^−1^	4.29 × 10^−16^	N/A; 4.56%; 99.70–105.39%; LOQ: 25 fg mL^−1^
2018, Aydın; [282]	Impedimetric immunosensor (electrode); electrochemical impedance spectroscopy (EIS), and single frequency impedance (SFI) techniques	IL-8	Cancer biomarker	6 fg mL^−1^	7.10 × 10^−16^	0.02 pg mL^−1^–3 pg mL^−1^; N/A; N/A; LOQ: 19 fg mL^−1^; R^2^ = 0.999
2018, Khan; [48]	Hybrid nanocomposite of graphene nanoplatelets with diblock co-polymers and Au electrodes (GRP-PS67-b-PAA27-Au); EIS	PSA	Cancer biomarker	40 fg mL^−1^	1.19 × 10^−15^	1 pg mL^−1^–100 ng mL^−1^; N/A; 95–114%, 94–110%, and 95–106% for concentration of 0.1, 1, and 10 ng mL^−1^, respectively; R^2^ = 0.963; accuracy: 88.89%
2021, Ben Halima; [283]	Antibody functionalization of an ion-sensitive field effect transistor; electrochemical impedance spectroscopy	TNF-α	Non-cancer biomarker	1 pg mL^−1^	5.78 × 10^−14^	1–50 pg mL^−1^; N/A; 88–100%
2022, Sri; [284]	MoS_2_ nanoflower-based electrochemical biosensor, EIS	TNF-α	Cancer biomarker	0.202 pg mL^−1^	1.17 × 10^−14^	0.01–200 pg mL^−1^; 3.3%; N/A; sensitivity: 23.156 Ω pg^−1^ mL cm^−1^, incubation time of 30 min, LOQ: 0.01 pg mL^−1^
2023, Ben Halima; [285]	Electrochemical impedance spectroscopy	NT-proBNP	Heart failure biomarker	0.02 pg mL^−1^	2.35 × 10^−15^	0.02–1 pg mL^−1^; N/A; 99%
2023, Ruankham; [286]	Electrochemical impedance spectroscopy	NT-proBNP	Heart failure biomarker	5 fg mL^−1^	5.88 × 10^−16^	0.005–1 pg mL^−1^; N/A; N/A
2024, Zea; [287]	Paper-based electrochemical sensor	cortisol	Stress biomarker	0.81 ng mL^−1^	2.23 × 10^−9^	A sensitivity of 19 Ω ng mL^−1^ with a correlation coefficient of 0.99
2025, Nagdeve; [288]	Electrochemical impedance spectroscopy	microRNA-31	Cancer biomarker	70 pg mL^−1^	1.00 × 10^−11^	Linear range 10^−11^–10^−6^ M in buffer and 10^−11^–10^−7^ M in diluted serum
				700 pg mL^−1^	1.00 × 10^−10^	
2025, Saeed; [289]	Label-free impedimetric immunosensor	fibulin-2	Cardiac biomarker	1.64 pg mL^−1^	1.24 × 10^−14^	Linear range: 5–25 pg mL^−1^; NA; 98%; R^2^ = 0.97, LOQ = 4.96 pg mL^−1^, 30 min incubation time
2025, da Silva; [149]	Impedimetric electrochemical biosensor	CagA oncogenic protein	Gastric disease, cancer	109.9 fg mL^−1^	8.33 × 10^−16^	Detection range: 0.5 pg mL^−1^ to 3.3 ng mL^−1^, 15 min response time, R^2^ = 0.996, recovery = 105.09 % and 112.41 %, RSD = 10.75% and 10.74%
**Amperometry**				**Geometric Average**	**1.35 × 10^−11^**	
				**Median**	**8.76 × 10^−13^**	
2009, Wei; [290]	Electrochemical sensor, amperometry	IL-8 mRNA	Cancer biomarker	3.9 fM	3.90 × 10^−15^	5 fM to 50 pmol L^−1^; N/A; N/A; sensitivity: 90%; R^2^ = 0.98
		IL-8 protein	Cancer biomarker	7.4 pg mL^−1^	8.76 × 10^−13^	10–12, 500 pg mL^−1^; N/A; N/A; linearity: R^2^ = 1
2016, Torrente-Rodríguez; [291]	Electrochemical magnetobiosensors, amperometric measurements	IL-8	Cancer biomarker	72.4 pg mL^−1^	8.57 × 10^−12^	N/A; 5.0%; 102 ± 5%; R^2^ = 0.9992, LOQ: 241.3 pg mL^−1^
		IL-8mRNA	Cancer biomarker	0.21 nM	2.10 × 10^−10^	N/A; 7.7%; 94 ± 7%; R^2^ = 0.9955, LOQ: 0.69 nM
2018, Barhoumi; [292]	chronoamperometric immunosensor	TNF-α	Cancer biomarker	0.001 ng mL^−1^	5.78 × 10^−14^	0.001–0.03 ng mL^−1^; N/A; N/A; R2 = 0.993
2019, Barhoumi; [293]	Amperometry using a screen-printed gold electrode	TNF-α	Non-cancer biomarker	0.3 pg mL^−1^	1.73 × 10^−14^	1–15 pg mL^−1^; N/A; N/A; CV% = 22% for 2D–SPEAu; 8% for 3D–SPEAu (device precision); R^2^ = 0.992
2020, Muñoz-San Martín; [294]	Magnetic beads-based electrochemical amperometric immunosensor	HIF-1α	Cancer biomarker	76 pg mL^−1^	8.17 × 10^−13^	Range: 0.25–10.0 ng mL^−1^; 4.7%; N/A; LOQ: 254 pg mL^−1^; R^2^ = 0.996
2020, Selvarasu; [295]	Amperometric (i–t) technique	3-NT	Oxidative stress biomarker	9 nM	9.00 × 10^−9^	0.025–855.2 M; 2.45–3.12%; 94.61–96.24%
2025, Zhao; [296]	AuNP/Magnetic Bead-Enhanced Electrochemical Sensor	lactoferrin	Alzheimer biomarker	2 µg mL^−1^	2.50 × 10^−8^	Linear range: 2–32 µg mL^−1^; 3.85%; NA; R^2^ = 0.991, one minute response time
		amyloid β-protein (Aβ1-42)	Alzheimer biomarker	0.1 pg mL^−1^	2.22 × 10^−14^	Linear range: 0.1 pg mL^−1^ to 1 ng mL^−1^; 3.29%; N/A; R^2^ = 0.9618; one minute response time
2026, Deng; [297]	Electrochemical non-enzymatic glucose sensor based on a three-dimensional (3D) porous CS-MMT/MWCNTs-COOH	glucose	Diabetes biomarker	1.09 µM	1.09 × 10^−6^	Linear detection range: 0.001 mM–5.78 mM, sensitivity: 3.89 µA mM^−1^ cm^−2^, RSD: 1.09%, recovery values of 98.0–102.9% with relative standard deviations below 5.0% (n = 3).
**Other Techniques**				**Geometric Average**	**7.38 × 10^−12^**	
				**Median**	**8.43 × 10^−12^**	
2018, Song; [298]	3DN-CNTs sensor; electrochemiluminescence (ECL) detection	CYFRA-21-1	Cancer biomarker	0.5 ng mL^−1^	1.25 × 10^−11^	1–1000 ng mL^−1^; N/A; N/A; CVs = 2.38–8.60%; R^2^ = 0.993
2022, Sadrjavadi; [299]	Ab/AuNPs/MES; photolithography and electrodeposition techniques	neuron-specific enolase	Cancer biomarker	0.34 ng mL^−1^	4.36 × 10^−12^	1–750 ng mL^−1^; 3.1%; N/A

**Table 6:** The summary of analytical studies on colorimetric detection. Abbreviations: LOD—limit of detection, FoM—figures of merit, RSD—relative standard deviation, LOQ—limit of quantification, CKD—chronic kidney disease, OSCC—oral squamous cell carcinoma, TMB—3,3′,5,5′-tetramethylbenzidine.

Year, First Author [Ref.]	Method	Analyte	Biomarker for	LOD, M	Other FoM
**Colorimetric detection**			**Geometric Average**	9.15 × 10^−4^	
			**Median**	1.88 × 10^−5^	
2016, Pena-Pereira [320]	Paper-based analytical device	Thiocyanate	Tobacco smoke exposure	6.00 × 10^−5^	ND
2017, Dominguez [321]	Colorimetric enzymatic assay	Glucose	Diabetes mellitus	9.44 × 10^−6^	R^2^ = 0.9903, CV = 5%, working range: 0–18 mg/dL
2018, Santana-Jiménez [322]	Bienzymatic Paper-Based Sensor	Glucose	Diabetes mellitus	4.66 × 10^−5^	LOQ: 2.47 mg/dL, RSD: 4.33, sensitivity: 1.81 A.U./mg
2018, Soni [323]	Smartphone-based optical biosensor	Urea	CKD	1.73 × 10^−3^	A linear detection range of 10–260 mg/dL
2020, Pedone [324]	Colorimetric assay based on the peroxidase-mimetic activity of Pt nanozymes	Catalase (among many in total antioxidant capacity)	Oxidative stress-related conditions	1.20 × 10^−5^	5 min assay time, RSD = 6%, R^2^ = 0.96
		Uric acid (among many in total antioxidant capacity)		3.20 × 10^−5^	
2021, Moulahoum [325]	Paper-based sandwich lateral flow assay	Synthetic cannabinoid	Forensic substance abuse	1.6 × 10^−9^	Assay time: 5–10 min, range 1.78–1000 ng/mL, CV < 6%
	Paper-based Competitive Lateral Flow Assay			9.5 × 10^−10^	
2021, Pungjunun [326]	Dual-mode paper-based analytical device (µpumpPAD)	Thiocyanate	Tobacco consumption/smoker differentiation	2.0 × 10^−4^	RSD < 5%, width of linear range 5 ord. of magn., 20 µL sample
2022, Fabiani [327]	96-well wax-printed paper-based IA using magnetic beads and smartphone-assisted colorimetric readout	SARS-CoV-2 spike (S) protein	COVID-19 infection	(0.1 µg mL^−1^)	Dynamic range up to 10 µg mL^−1^, 100% agreement with RT-PCR
2022, Mollaie [328]	Colorimetric detection through a modified Griess reaction on a laser-cut µPAD	Nitrite	Various diseases	5.7 × 10^−6^	Detection range from 5 to 1000 µM, 70% mixing efficiency improvement, <5% deviation from clinical tests
2023, Chi [329]	Colorimetric biosensor	Glucose	Diabetes	2.55 × 10^−5^	ND
		Creatinine	Kidney diseases	5.22 × 10^−6^	
2023, Tarara [330]	Wax-patterned µPAD colorimetric assay using methylthymol blue	Calcium	Dental erosion, active carious lesions	7.24 × 10^−5^	Linear range: 5 to 40 mg L^−1^, R^2^ = 0.983, LOQ = 8.9 mg L^−1^, intra-day precision < 5.1%, interday precision < 6.4%
2025, Franco [331]	Paper-based colorimetric sensor using an epoxy-Ag nanocomposite	Chloride anions	Cystic fibrosis	1.4 × 10^−2^	Working range: 20–400 mM, R^2^ = 0.9754, response time: 10–12 s, down to 4 µL sample
2025, Ming [332]	Colorimetric sensing	Oral cancer overexpresses the 1 gene (ORAOV1 gene)	OSCC	1.00 × 10^−8^	ND
2025, Wang [333]	Direct in situ colorimetric and red-fluorescent dual-readout biosensor	Glucose	Diabetes Mellitus	4.60 × 10^−7^	R^2^ = 0.995
2026, Bayoumy [334]	Colorimetric lactate biosensor using lactase oxidase, Fe^3+^, and TMB for blue detection.	L-lactate	Tissue hypoperfusion, metabolic stress	1.278 × 10^−6^	Linear range: 5–20 µM, LOQ = 3.871 µM, R^2^ = 0.9895, recovery 96.53% to 104.85%
2026, Kumar [335]	Microfluidic paper-based analytical device (µPAD)	Phosphate	Hyperphosphatemia, CKD	2.77 × 10^−4^	Sensitivity: 0.262 ± 0.022, mean bias of 0.42%, RSD 15.5%

**Table 7:** The summary of analytical studies on cortisol detection. For the calculation of “converted LOD”, we used the molecular weight of cortisol, 362.46 Da, as reported in the literature [344]. Abbreviations: SPR—surface plasmon resonance, LC-MS/MS—liquid chromatography with tandem mass spectrometry, HPLC-MS/MS—high performance liquid chromatography coupled with tandem mass spectrometry, EIS—electrical impedance spectroscopy, rGO—reduced graphene oxide, LOD—limit of detection, FoM—figures of merit, RSD—relative standard deviation, LOQ—limit of quantification, LLOQ—lower limit of quantification.

Year, First Author; [Ref]	Method	Reported LOD	LOD (M)	Other FoM
**Immunoassays–Optical**		**Geometric Average**	**3.91 × 10^−11^**	
		**Median**	**6.37 × 10^−11^**	
2014, Tahara; [345]	SPR-based immunoassay	38 ppt	1.05 × 10^−10^	Linear range: 10 ppt–100 ppb
2018, Apilux; [252]	Lateral flow immunoassay	0.5 ng mL^−1^	1.38 × 10^−9^	Linear range: 0.5–150 ng mL^−1^, RSD: 1.8–4.2%, total assay time: 15–20 min
2018, Oh; [253]	Trap lateral flow assay	9.9 pg mL^−1^	2.73 × 10^−11^	Linear range: 0.01–100 ng mL^−1^
2019, Abdulsattar; [254]	Chemiluminescence-based immunoassay	0.47 pg mL^−1^	1.30 × 10^−12^	Linear range: 0–50 ng mL^−1^, recovery: 91%, RSD: 1.25%
2020, Jo; [346]	SPR-based aptasensor	0.1 nM	1.00 × 10^−10^	Linear range: 0.1–1000 nM
2021, Huang; [347]	Aptamer-antibody sandwich cortisol sensor	0.09 pg mL^−1^	2.48 × 10^−13^	Linear range: 0.1 pg mL^−1^–10 ng mL^−1^, RSD: 2.42–3.89%, recovery: 96.9–104.04%
2024, Kim; [259]	Automatic signal-enhanced lateral-flow immunoassay (asLFI)	3.8 pg mL^−1^	1.05 × 10^−11^	Detection range: 0.01–10 ng mL^−1^, R^2^ = 0.9159
2025, Kim; [260]	Competitive Lateral Flow Assay with colorimetric sensing based on AuNPs	1.473 ng mL^−1^	4.06 × 10^−9^	Recovery: 90%, R^2^ = 0.99, 2 min assay time, no external equipment
**Mass-Spectroscopy**		**Geometric Average**	**1.71 × 10^−10^**	
		**Median**	**8.28 × 10^−11^**	
2013, Kataoka; [348]	LC-MS/MS	0.03 ng mL^−1^	8.28 × 10^−11^	RSD < 8.5%, recovery: 94%
2015, Antonelli; [342]	LC-MS/MS	55.4 nmol L^−1^	5.54 × 10^−8^	RSD < 10%, LLOQ of 0.51 nmol L^−1^
2019, Bakusic; [349]	LC-MS/MS	5 pg mL^−1^	1.38 × 10^−11^	Intra−assay accuracy 95–97%, RSD: 2–3%
2020, Chen; [350]	HPLC-MS/MS	100 pg mL^−1^	2.76 × 10^−10^	RSD < 9.68%; lower limit of quantification (LLOQ): 0.1 ng mL^−1^
2020, Ertuğrul; [351]	LC-MS/MS	0.02 nM	2.00 × 10^−11^	LOQ: 0.05, linearity: 0.05–300 nM, recovery: 98%
2021, Grau; [229]	LC-MS/MS	0.029 ng mL^−1^	8.00 × 10^−11^	Linear range: 0.3–20 ng mL^−1^, RSD: 4.2–10%, recovery: 86–111%
2024, Lanfermeijer; [352]	LC-MS/MS	0.15 nmol L^−1^	1.50 × 10^−10^	Imprecision: 14%, R^2^ = 0.964
**Electrical Impedance Spectroscopy (EIS)**		**Geometric Average**	**2.10 × 10^−11^**	
		**Median**	**4.41 × 10^−11^**	
2010, Arya; [353]	EIS	1 pM	1.00 × 10^−12^	Linearity: 1 pM–100 nM
2011, Liu; [341]	EIS	16 pg mL^−1^	4.41 × 10^−11^	Linearity: up to ~2500 pg mL^−1^ (~10^−9^ M)
2020, Zhou; [354]	EIS	0.023 pM	2.30 × 10^−14^	Linearity: 0.1–1500 pM; recovery: 93.96–96.65%
2022, Ben Halima; [355]	EIS	0.66 ng mL^−1^	1.82 × 10^−9^	Linearity: 2–50 ng mL^−1^
2024, Zea; [287]	Paper-based electrochemical sensor	0.81 ng mL^−1^	2.23 × 10^−9^	A sensitivity of 19 Ω ng mL^−1^ with a correlation coefficient of 0.99
**Voltametry**		**Geometric Average**	**6.25 × 10^−12^**	
		**Median**	**2.86 × 10^−12^**	
2008, Sun; [356]	Cyclic voltammetry	0.76 nM	7.60 × 10^−10^	Incubation time: 10 min
2019, Dhull; [357]	Cyclic voltammetry, differential pulse voltammetry	0.32 pg mL^−1^	8.83 × 10^−13^	Linearity: 1 pg mL^−1^–10 g mL^−1^
2020, Liu; [358]	Smartphone-based differential pulse voltammetry	0.11 nM	1.10 × 10^−10^	Linearity: 0.5–200 nM; recovery: 96.3–105.64%
2022, Rezapoor-Fashtali; [359]	Differential pulse voltammetry	0.045 pM	4.50 × 10^−14^	Linearity: 0.05–15 pM; RSD < 3.7%
2025, Nampeng; [360]	Cyclic voltammetry	1.035 pg mL^−1^	2.86 × 10^−12^	Detection range: 0.1–1000 pg mL^−1^
**Other: Chemiresistors, Electrical Chips, Colorimetric, etc.**		**Geometric Average**	**2.63 × 10^−11^**	
		**Median**	**1.79 × 10^−11^**	
2011, Tlili; [361]	Label-free chemiresistive immunosensor	1 pg mL^−1^	2.76 × 10^−12^	Linear range: 1 pg mL^−1^–10 ng mL^−1^, RSD: 1.3–3.9%
2017, Khan; [362]	Label-free paper-based electrical biosensor chip	3 pg mL^−1^	8.28 × 10^−12^	Linearity: 3 pg mL^−1^–10 g mL^−1^
2017, Kim; [363]	rGO chemisresistor sensor	10 pg mL^−1^	2.76 × 10^−11^	Linearity: 1–10 ng mL^−1^
2023, Badi’ah; [364]	Colorimetric sensor	0.76 nM	7.60 × 10^−10^	LOQ: 2.54 nM, R^2^ = 0.9998; accuracy: 91–103%; precision < 2.2%; recovery: 91–95%

**Table 8:** The summary of polymerase chain reaction (PCR) based clinical studies. Studies reporting sample sizes of fewer than 40 patients or presenting sensitivity and/or specificity as ranges rather than discrete values were excluded from the calculation of average sensitivity and specificity. Such studies are marked with an asterisk (*).

Year, First Author; [Ref.]	Method	Disease	Biomarker Type	Sample Size	Sensitivity	Specificity	Other FoM
**Polymerase Chain Reaction (PCR) Based Clinical Studies**			**Average**	**114**	**84.90%**	**83.77%**	
			**Median**	**67**	**85.70%**	**85.55%**	
2015, Humeau *; [375]	qRT-PCR	pancreatic cancer	microRNA	21 (17+, 4−)	71.40%	100%	ND
	qPCR		miRNA (hsa-miR-21)	17 (13+, 4−)	100%	71%	*p* = 0.012
2015, Yan *; [29]	qPCR	lung cancer squamous cell carcinoma (SCC) and adenocarcinoma (AC)	microbes (Capnocytophaga and Veillonella)	30 (20+, 10−)	84.60%	86.70%	ROC: 0.86 in distinguishing patients with SCC from control subjects
2015, Yan; [29]	qPCR	lung cancer	microbiome	56 (41+, 15−)	84.60%	86.70%	ROC: 0.86
2015, Xie; [379]	qPCR	pancreatic cancer (PC)	miR-3679-5p	100 (40 PC, 20 benign PC, 40 healthy)	85%	45%	*p* = 0.003, AUC: 0.688
2016, Xie; [31]	qPCR	pancreatic cancer	IncRNAs (non-coding RNAs)	130 (75+, 55−)	78.20%	90.90%	AUC: 0.880; *p* < 0.001
2017, Liu *; [33]	qPCR	pancreatic cancer (PC)	PC-related genes	114 (± no data)	93.5–96.7%	71.4–100%	Analysis of 29 novel biomarkers based on the expression of 516 genes in saliva
2018, Li; [380]	RT-qPCR	gastric cancer	transcriptome	294 (163+, 131−)	75%	83%	ROC curve (AUC) of 0.81; (95% CI, 0.72–0.89).
2018, Min; [75]	PCR	hand, foot, and mouth disease (HFMD)	miRNAs	58 (35+, 24−)	100%	88.90%	Non-parametric Mann–Whitney test using Prism
2019, Lee; [381]	qRT-PCR	Sjogren’s syndrome (oral disease)	lectins (soluble siglec-5)	90 (45+, 45−)	64.40%	77.80%	AUC: 0.711 (95% CI 0.602–0.820); cut-off ≥ 200 pg mL^−1^
2019, Márton; [382]	qPCR	OSCC	mRNAs (IL-6 mRNA)	175 (90+, 85−)	94.50%	81.90%	AUC: 0.9379 (*p* = 0.001; 95% confidence interval: 0.8973–0.9795)
2020, Al-Rawi; [383]	qPCR	periodontitis (oral disease)	miRNA-155	53 (24+29−)	86.70%	78.60%	AUC: 0.861 CT value: 8.975 (cutoff value)
2020, He; [384]	RT-qPCR	OSCC	miRNAs (miR-24-3p)	55 (45+, 10−)	64.40%	80%	AUC: 0.738 (95% CI: 0.589–0.886, *p* = 0.02)
2020, Kim; [385]	qPCR	periodontitis	pathogens	692 (548+, 144−)	71%	84%	AUC: 0.82 diagnostic odds ratio: 12.85
2020, Oh; [386]	RT-PCR	OSCC	transcriptome	67 (34+, 33−)	92%	86%	AUC: 0.91
2020, Sehovic; [23]	qRT-PCR	autism spectrum disorder (ASD)	miRNAs	80 (55+, 25−)	90.32%	90%	AUC: 0.952
2021, Mehterov; [387]	TaqMan assay	OSCC (Survival rates of oral squamous cell carcinoma)	miRNAs	45 (33+, 12−)	86%	74%	AUC: 0.82; 95% CI: 0.71–0.89
2021, Romani; [388]	RT-qPCR	OSCC (Survival rates of oral squamous cell carcinoma)	miRNAs	147 (89+, 58−)	85.40%	85.10%	AUC: 0.923
2021, Ueda; [389]	RT-PCR	OSCC	Pathogens (CCL20)	133 (83+, 50−)	100%	98%	ND
2021, Zhang; [376]	qPCR	periodontitis	Proteome (IL-1β)	80 (55+, 25−)	90%	76%	AUC: 0.88; cutoff: 91.1 pg mL^−1^
2024, Ayalew; [390]	qPCR	pulmonary tuberculosis	IS1081 gene	62 (32+, 30−)	65.60%	96.70%	AUC: 82.5% (95% CI: 71.7–93.3%)
2024, Katsuno; [391]	RT-PCR	SARS-CoV-2	RNA	52 (25+, 27−)	100.00%	89.10%	Cohen’s kappa value: 0.89
2025, Wityk; [392]	RT-qPCR	SARS-CoV-2	Viral RNA	60 (12+, 48−)	100%	100%	*p* < 0.05

**Table 9:** The summary of mass spectroscopy-based clinical studies. Studies reporting sample size of fewer than 40 patients, or presenting sensitivity and/or specificity as ranges rather than discrete values, were excluded from the calculation of average sensitivity and specificity. Such studies are marked with an asterisk (*).

Year, First Author; [Ref.]	Method	Disease	Biomarker Type	Sample Size	Sensitivity	Specificity	Other (Statistical Method, AUC, ROC)
**Mass Spectroscopy-Based Clinical Studies**			**Average**	**137**	**81.31%**	**88.54%**	
			**Median**	**58**	**80.00%**	**87.70%**	
2015, Cheng; [27]	ultra-performance liquid chromatography–mass spectrometry (UPLC–MS)	breast cancer	salivary free amino acids	55 (27+, 28−)	ND	88.20%	AUC—0.916; R^2^ > 0.99; LOD: 0.94–237.62 ng mL^−1^; LOQ: 3.14–792.07 ng mL^−1^; recoveries: 95.4–106.2%; intra-day CVs < 5%; inter-day CVs < 7%
2015, Liang; [24]	FUPLC-MS	Alzheimer’s disease	metabolites (sphinganine-1-phosphate, etc.)	474 (256+, 218−)	99.40%	98.20%	AUC: 0.998
2016, Takayama; [403]	UPLC-MS/MS	breast cancer	polyamines	172 (111+, 61−)	80%	80%	UPLC–MS/MS analysis; R^2^ > 0.99; 95% < (freeze/thaw stability); 8.3% > (intra-day and inter-day precision); 98% < recovery; AUC: 0.5–0.7 for different polyamines
2016, Zhong; [49]	UPLC-MS	breast cancer	metabolome (LysoPC 18:1)	55 (30+, 25−)	77.80%	100%	AUC: 0.93; standard error = 0.0042; 95% CI: 0.836–1.000
2018, Chen; [34]	HPLC-MS	Gastric cancer	aminoacids	220 (20 EGC, 84 AGC, 116 controls)	80%	87.70%	AUC: 0.690–0.900
2020, Deutsch *; [401]	quantitative-mass-spectrometry analysis (qMS)	pancreatic cancer	proteome	31 (15+, 16−)	90%	90%	AUC: 0.910
2020, Xavier Assad; [50]	LC-quadrupole TOF MS	breast cancer	metabolome (glycerophospholipids and oligopeptides)	58 (23+, 35−)	65.20%	77.10%	AUC: 0.732
2021, Zhang; [230]	HILIC-UPLC-HRMS	thyroid cancer	metabolome (L-alanine (Ala))	122 (61+, 61−)	91.20%	85.20%	R^2^ > 0.99; intra-assay RSD < 7%; AUC 0.936
2025, Blanco-Pintos; [404] *	sequential window acquisition of all theoretical mass spectra (SWATH-MS)	periodontitis	salivary proteins	85 (41+, 44−)	90.2–98.60%	93.6–97.2%	Accuracy: 98.2%
2025, Serdar; [405]	Liquid chromatography-mass spectrometry	periodontitis	trimethylamine N-oxide (TMAO)	48 (24+, 24−)	58.3%	83.3%	AUC: 0.747

**Table 10:** The summary of ELISA, an immunoassay-based clinical study.

Year, First Author; [Ref.]	Method	Disease	Biomarker Type	Sample Size	Sensitivity	Specificity	Other FoM
**ELISA & Immunoassay-Based Clinical Studies**			**Average**	101	81.8%	83.7%	
			**Median**	88	85.0%	84.7%	
2015, Adornetto; [35]	ELIME assay (enzyme-linked immunomagnetic electrochemical assay	celiac disease	antibodies (anti-tTG IgA)	66 (± not given) blind sample analysis approach II By approach I, 72(17+, 55−)	95%	96%	Ab index: 0.022; AUC: 1.00
2015, Ebersole; [409]	Luminescent assay (MILLIPLEX MAP Kit)	periodontitis	IL-1b	209 (144+, 65−)	75.20%	75.90%	Cut-off value of 24 pg mL^−1^
2016, Jacobs; [410]	Luminex multiplex immunoassay	tuberkulosis	proteins	104 (32+, 72−)	78.10%	83.30%	AUC: 0.70–0.85
2016, Xiao; [30]	ELISA	gastric cancer	proteins	40 (20+, 20−)	85%	80%	ROC: 0.81–0.92; *p* < 0.05
2017, Namuganga; [411]	luminex immunoassay	tuberkulosis	proteins (G-CSF, TNF-α, VEGF)	78 (39+, 39−)	63%	63%	AUC: 0.63–0.85
2018, Koizumi; [412]	multiplex bead array assay	lung cancer	cytokines	70 (35+, 35−)	60.60%	80.80%	AUC: 0.701, *p* = 0.000; 95% CI: 0.602–0.801; cutoff values: 0.702–0.708
2018, Omran; [46]	ELISA	neonatal pneumonia	proteins (CRP)	70 (35+, 35−)	91.40%	80.90%	cutoff value of salivary CRP—3.8 ng L^−1^
2018, Wu; [413]	ELISA	periodontitis	IL-1β, IL-1ra, MMP-9	57 (30+, 27−)	73.30%	89.90%	AUC: 0.853
2019, Cao; [25]	Electrochemiluminescence (ECL) assays	Parkinson’s disease	Exosomes (α-Synuclein)	134 (74+, 60−)	92%	86%	AUC: 0.941
2019, Feng; [395]	ELISA	OSCC	kallikrein 5	80 (60+, 20−)	70%	86.70%	AUC: 0.843; 95% CI: 0.743–0.942
2020, Kim; [414]	ELISA	peridontitis	proteome (MMP-9, S100A8)	149 (99+, 50−)	85%	76%	AUC: 0.860
2020, Riis; [415]	immunoassay	human cytomegalovirus (HCMV)	HCMV IgG	96 (35+, 61−)	51%	97%	Detection limit: 0.01 U mL^−1^; intra-assay precision: 5%
2020, Sivadasan; [416]	ELISA	oral leukoplakia	proteome CD44	60 (45+, 15−)	91.60%	72.70%	AUC: 0.712
2024, Raheem; [417]	Enzyme-linked immunosorbent assay	periodontitis	Salivary IL-1β	135	98.00%	95.00%	Unstable from stable periodontitis (AUCs = 0.98, 0.99, and 1; sensitivity = 0.94, 1, and 1; specificity = 0.94, 0.97, and 1, respectively).
			IL-10		100.00%	95.00%	
			IL-1β/IL-10		100.00%	100.00%	

**Table 11:** The summary of Raman spectroscopy- and other techniques-based clinical studies. Studies reporting a sample size of fewer than 40 patients or presenting sensitivity and/or specificity as ranges rather than discrete values were excluded from the calculation of average sensitivity and specificity. Such studies are marked with an asterisk (*).

Year, First Author; [Ref.]	Method	Disease	Biomarker Type	Sample Size	Sensitivity	Specificity	Other (Statistical Method, AUC, ROC)
**Raman Spectroscopy-Based Clinical Studies**			**Average**	110	83.6%	89.6%	
			**Median**	106	82.7%	90.5%	
2015, Cao; [428]	Raman spectroscopy	Acute myocardial infarction (AMI)	Aminoacids	89 (46+, 43−)	80.40%	81.40%	AUC = 0.855
2015, Feng; [28]	SERS	Benign and malignant breast tumors	Proteins	97 (33 HC, 33 BBT, 31 MBT)	72.70%	81.25%	AUC: benign breast tumor—0.972, malignant—0.975
2017, Hernández-Arteaga; [429]	SERS	Breast cancer	Sialic acid	206 (100+, 106−)	94%	98%	Cutoff value > 7 mg dL^−1^
2018, Zermeño-Nava; [32]	SERS	Ovarian cancer	Sialic acid	52 (37 benign, 15 ovarian cancer)	80%	100%	Cutoff value: 15.5 mg dL^−1^, AUC = 0.941
2019, Hernández-Arteaga; [430]	SERS	Breast cancer	Sialic acid	164 (35+, 129−)	80%	93%	AUC: 0.95; cutoff: 12.5 mg dL^−1^
2019, Zamora-Mendoza; [431]	SERS	Asthma	Proteins	44 (26+, 18−)	85%	82%	ND
2025, Sunil; [432]	SERS	Oral cancer	Oral cancer markers (nitrate, nitrite, thiocyanate, proteins, and amino acids)	116 (56+, 60−)	88%	92%	Accuracy: 87.5%
2026, Puravankara; [433]	SERS	Oral cancer	Oral cancer-related salivary spectro-molecular signatures	114 (76+, 38−)	89%	89%	ROC-AUC = 0.96; healthy vs. premalignant: sensitivity: 66.7%, specificity: 100%, accuracy: 82.4%
**Other Techniques-Based Clinical Studies**			**Average**	**208**	**73.61%**	**82.57%**	
			**Median**	**80**	**83.0%**	**86.6%**	
2009, Wei; [290]	Electrochemical sensor	Oral cancer	IL-8 mRNA	56 (28+, 28−)	83%	87%	AUC: 0.90
2016, Dy; [434]	pH-multichannel intraluminal impedance testing	Gastroesophageal reflux disease (GERD)	Pepsin	50 (21+, 29−)	42%	58%	Median salivary pepsin concentration of 10 ng mL^−1^
2017, Shu; [435]	Lectin blotting analysis	Gastric cancer	Lectins	124 (94+, 30−)	96%	80%	AUC: 0.89; 95% CI: 0.80–0.99; cutoff value: 0.91; st. error: 0.046
2018, Kaplan; [436]	Western blotting	Multiple sclerosis	Immunoglobulin free light chains (FLC)	113 (85+, 28−)	88.50%	100%	ND
2018, Liang *; [378]	Proteomic analysis	Sjögren syndrome	Proteins (e.g., RAC2)	26 (13+, 13−)	92.30%	92.30%	AUC: 0.947; *p*-value < 0.001
-2018, Liu; [437]	Lectin blotting analysis	Breast cancer	Lectins	447 (337+, 110−)	73.30%	74.20%	AUC: 0.755
2019, Rodrigues *; [438]	ATR-FTIR spectroscopy	Chronic kidney disease	Thiocyanate, Phospholipids/Carbohydrates	28 (14+, 14−)	92.80%	85.70%	AUC: 0.88
2020, Bel’skaya; [439]	Biochemical analysis	Lung cancer	Metabolome	1143 (593+, 550−)	69.50%	87.50%	AUC: 0.830
2021, Davidson [440]	Paper-based colorimetric RT-LAMP (pH-sensitive dye phenol red)	SARS-CoV-2	SARS-CoV-2 gene, RNA	60 (30+, 30−)	97.00%	100.00%	LOD: 200 genomic copies/µL
2021, Deng; [441]	aMMP-8 index test	Periodontitis	Metalloproteinase-8 (MMP-8)-enzyme	408 (102+, 306−)	33.20%	93%	Threshold level > 10 ng mL^−1^
2021, López-Jornet; [442]	Biochemical analysis	Breast cancer	Proteome	151 (91+, 60−)	62.50%	59.40%	Cutoff value: 165.4 pg mL^−1^
2023, Bordbar [443]	Colorimetric-based multiple sensor	Diabetes	All saliva metabolites	80 (41+, 39-)	91.10%	86.60%	Accuracy: 88.9%
2024, Guevara-Vega *; [444]	ATR-FTIR	CHIKV infection	Chikungunya virus	13 (6+, 7−)	83.00%	86.00%	Accuracy: 85%

**Table 12:** Summary of clinical performance of different methods in terms of sensitivity and specificity.

Method	Average		Median		Average AUC (#)	Balanced Accuracy	Total #: References
	Sens.	Spec.	Sens.	Spec.			
Mass spectroscopy	78.8%	87.5%	80.0%	87.7%	0.84 (9)	83.2%	Total 9: [24,27,34,49,50,230,401,403,405]
PCR	84.9%	83.8%	85.7%	85.6%	0.84 (16)	84.3%	Total 22: [23,29,31,33,75,375,376,379,380,381,382,383,384,385,386,388,389,390,391,392]
ELISA	88.3%	84.1%	91.4%	86.7%	0.89 (6)	86.2%	Total 7: [30,46,395,413,414,416,417]
Immunoassays	73.6%	83.1%	75.2%	83.3%	0.71 (6)	78.4%	Total 7: [25,35,409,410,411,412,415]
SERS & Raman	83.6%	89.6%	82.7%	90.5%	0.94 (5)	86.6%	Total 8: [28,32,428,429,430,431,432,433]
Direct SERS	84.1%	90.7%	85.0%	92.0%	0.96 (4)	87.4%	Total 7: [28,32,429,430,431,432,433]
Other (electrochemical methods, IR, Western blotting, biochemical analysis, colorimetric)	73.6%	82.6%	83.0%	86.6%	0.80 (9)	78.1%	Total 13: [290,378,434,435,436,437,438,439,440,441,442,443,444]

Note: the table summarizes the studies with cancer/non-cancer related biomarkers and analytes. For the summary of studies with viruses/bacteria, please refer to Table 2 in Section 2. For the papers with reported range or multiple values of AUC, the midpoint of the range was taken for the calculations. The “#” sign indicates the number of publications. Studies reporting a sample size of fewer than 40 patients or presenting sensitivity and/or specificity as ranges rather than discrete values were excluded from the calculation of average sensitivity and specificity.

## Data Availability

No new data were created or analyzed in this study. Data sharing is not applicable to this article.
